# Design, synthesis and biological evaluation of edaravone derivatives bearing the *N*-benzyl pyridinium moiety as multifunctional anti-Alzheimer’s agents

**DOI:** 10.1080/14756366.2020.1801673

**Published:** 2020-08-11

**Authors:** Luke S. Zondagh, Sarel F. Malan, Jacques Joubert

**Affiliations:** Pharmaceutical Chemistry, School of Pharmacy, University of the Western Cape, Bellville, South Africa

**Keywords:** Alzheimer’s disease, edaravone, *N*-benzyl pyridinium, cholinesterase, oxidative stress

## Abstract

A series of multi-target directed edaravone derivatives bearing *N*-benzyl pyridinium moieties were designed and synthesised. Edaravone is a potent antioxidant with significant neuroprotective effects and *N*-benzyl pyridinium has previously exhibited positive results as part of a dual-site binding, peripheral anionic site (PAS) and catalytic anionic site (CAS), acetylcholinesterase (AChE) inhibitor. The designed edaravone-*N*-benzyl pyridinium hybrid compounds were docked within the AChE active site. The results indicated interactions with conserved amino acids (Trp279 in PAS and Trp84 in CAS), suggesting good dual-site inhibitory activity. Significant *in vitro* AChE inhibitory activities were observed for selected compounds (IC_50_: 1.2–4.6 µM) with limited butyrylcholinesterase inhibitory activity (IC_50_’s >160 µM), indicating excellent selectivity towards AChE (SI: 46 – >278). The compounds also showed considerable antioxidant ability, similar to edaravone. *In silico* studies indicated that these compounds should cross the blood–brain barrier, making them promising lead molecules in the development of anti-Alzheimer’s agents.

## 1. Introduction

Alzheimer’s disease (AD) is a progressive neurodegenerative disorder that is mainly prevalent in the older population (>65 years of age)^1–3^. Approximately fifty million people are diagnosed with dementia, with AD accounting for 60–70% of these cases^4^. The disease can be characterised by an array of symptoms which include; memory loss, cognitive impairment, behavioural and psychiatric abnormalities^3^. Due to the complex and multifactorial nature of AD, the exact aetiology of the disease is unknown. Multiple pathways and hypotheses have been indicated in the pathology of the disease such as the Aβ cascade-, cholinergic- and the oxidative stress hypotheses^1^^,^^3^^,^^5^.

Senile plaques are considered a pathological hallmark of AD. The primary constituent of these senile plaques is Aβ and are believed to play a central role in the pathogenesis of the disease^6^^,^^7^. The amyloid cascade hypothesis suggests that one of the main driving forces behind AD development is the buildup and deposition of Aβ peptide aggregation within the brain^8^^,^^9^. Recently, it was discovered that the amyloid precursor protein (APP) gene undergoes mutations that induce an increase in Aβ formation. The APP mutations are situated near the sites where proteases, β and γ-secretase, cleave the APP. These mutations result in the favouring of the Aβ_1–40_ and Aβ_1–42_ peptide fragment formation^7^^,^^8^^,^^10^. Aβ_1–40_ and Aβ_1–42_ are more inclined to self-aggregate to form amyloid beta fibrils. With the persistent imbalance of the production and clearance of the Aβ fragments, the consequential result is the genesis of insoluble senile plaques. These senile plaques result in the blockage of parenchymal spaces between neurons in the brain leading to eventual neuronal cell death^8^^,^^9^.

The cholinergic hypothesis describes that the hydrolysis of the neurotransmitter acetylcholine (ACh) by cholinesterases, acetylcholinesterase (AChE) and butyrylcholinestrase (BuChE), leads to a drastic decrease in ACh levels. The loss of cholinergic transmission due to the decreased levels of ACh has been correlated with loss of memory and cognitive ability^11^. AChE is the predominant enzyme that hydrolyse ACh in the healthy brain^12^. The AChE enzyme contains a pocket with two binding sites, the catalytic anionic site (CAS) and peripheral anionic site (PAS). Interactions with both these sites are crucial for the inhibition of AChE activity and potential neuroprotective effects^13^. The PAS possesses a non-cholinergic role that through protein–protein interactions, bind to and promotes the formation and deposition of insoluble Aβ fibrils leading to neurotoxicity. Recent studies have shown that the inhibition in the PAS did not only improve the memory in a transgenic APP/PS1 murine model, but also significantly stemmed the amount of Aβ plaques in the brain^14–16^.

Reactive oxygen species (ROS) are known to play a significant role in the progression of neurodegenerative disorders such as AD^17–19^. The most significant ROS include hydroxyl radicals, superoxide anions and peroxyl radicals^19^. As ROS begins to accumulate and antioxidant levels begin to reduce, detrimental effects in the brain begin to occur. This process is further increased with aging^17^^,^^19^. The brain is especially susceptive to the neurotoxic effects of ROS due to its high demand for oxygen as well as the large amounts of peroxide susceptible lipid cells^18^. ROS have also been observed to cause disruptions in neuronal cell integrity and to modify and inactivate several proteins that are important for glucose metabolism and ATP synthesis resulting in mitochondrial dysfunction^10^^,^^18^. The occurrence of the neurotoxic effects of ROS in the development of AD, coupled with the presence of Aβ, supports the role of oxidative stress in the pathogenesis of AD^18^^,^^20^^,^^21^. Several lines of evidence have revealed a connection between oxidative stress and Aβ formation^10^^,^^19^^,^^22^. Aβ exhibits the ability to enhance the formation of ROS and *vice versa*^22^^,^^23^. Aβ produces ROS through the promotion of oxidative modification and inhibition of important transmembrane transports systems within the neuronal and glial cells, Aβ-induced lipid peroxidation and protein oxidation^22^^,^^24^^,^^25^. In addition, ROS also stimulate the enhanced activity of proteases, β and γ-secretase, which increases the formation of Aβ_1–40_ and Aβ_1–42_^23^.

At present, there are no therapeutic agents that are able to reverse, halt or slow the progression of the disease and the current options are only able to treat AD symptomatically^2^^,^^11^^,^^26–28^. All of these treatments follow the much researched “one-molecule–one-target” drug discovery approach with minimal success. Therefore, more researchers are exploring the development of multi-target directed ligands (MTDL)^26^. MTDLs are conceived from the molecular hybridisation of various pharmacophoric moieties from recognised bioactive compounds. The MTDLs are designed to interact with multiple targets involved in the multifactorial pathogenesis of AD. The rational decision to combine these pharmacophores results in greater selectivity for the targets of AD, leads to fewer side effects and potentially improves the compounds’ neuroprotective abilities^29^^,^^30^.

**Figure 1.**
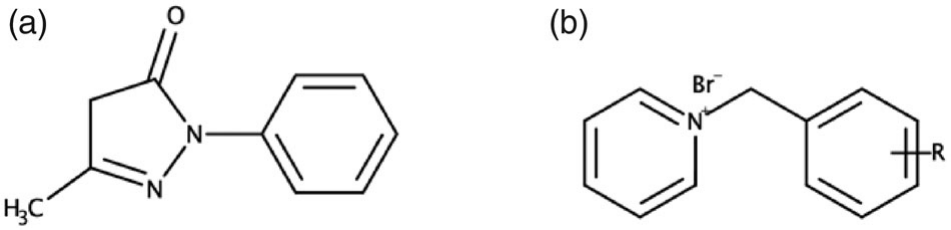
The two moieties combined to synthesise the novel MTDLs in this study. (a) Edaravone. (b) R-substituted *N*-benzyl pyridinium.

Edaravone (Figure 1(a)) is a potent free radical scavenger used to treat acute cerebral infarction in Japan^31^. In addition, edaravone has also exhibited beneficial neuroprotective effects in amyotrophic lateral sclerosis (ALS) and Parkinson’s disease animal models^32^. Edaravone’s neuroprotective effects are believed to be caused by its ability to scavenge ROS. The decrease in ROS levels in turn reduces oxidative stress and oxidative damage to neuronal cells^33^^,^^34^. In previous studies, edaravone has demonstrated the ability to attenuate Aβ-induced oxidative stress and neurotoxicity, inhibit Aβ aggregation, disaggregate preformed Aβ fibrils and attenuate downstream pathologies including tau-hyperphosphorylation, neuroinflammation and neuronal cell loss^31^^,^^35^.

In search for potentially potent and selective AChE inhibitors, benzyl pyridinium salts have been extensively researched (Figure 1(b))^36–39^ and *N*-benzyl pyridinium moieties have demonstrated excellent activity against AChE. Previous research has found that the best AChE inhibitory activity is reached when the *N*-benzyl pyridinium moiety is bound to another privileged molecule, using the MTDL strategy, to form a dual-site (PAS and CAS) binding compound^37^^,^^38^. Substitutions, e.g. halogens and methyl groups, at various positions on the benzyl group of the moiety has demonstrated improved AChE inhibitory activity compared to an unsubstituted benzyl ring^36^^,^^40^.

**Figure 2.**
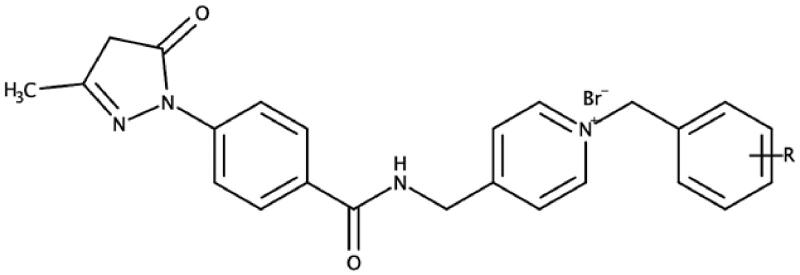
Edaravone-*N-*benzyl pyridinium hybrid compounds designed and evaluated in this study. R = H, Br, F, Cl, or CH_3_.

Thus, we describe here the docking, synthesis and biological evaluation of new edaravone-*N*-benzyl pyridinium hybrid compounds (Figure 2). These compounds are expected to exhibit strong dual-site AChE inhibitory activities and significant antioxidant capacity, which could lead to promising MTDL neuroprotective effects.

## 2. Materials and methods

### 2.1. Chemistry

All the reagents used to synthesise the desired compounds were acquired from Sigma-Aldrich® or Industrial Analytical (Pty) Ltd. All the reagents were used without further purification. Solvents used in the synthesis and purification of the compounds were obtained from a variety of commercial sources. Thin-layer chromatography (TLC) was used to monitor all reactions and was carried out on 0.20 mm thick aluminium silica gel sheets (TLC silica gel 60 F245 Merck KGaA). Visualisation of the samples was achieved using UV light (254 nm and 366 nm) and iodine vapours. Mobile phases were prepared on a volume-to-volume basis. Infra-red spectra were acquired using a Perkin Elmer Spectrum 400 spectrometer. The IR spectrometer was equipped with a diamond attenuated total reflectance (ATR) attachment. The spectra were then acquired from PerkinElmer, Inc. Spectrum version 10.5.4 IR software. The MS spectra of the compounds were acquired from a Waters SYNAPT G2 high resolution mass spectrometer. The melting points of the samples were acquired using a Lasec Melting Point SMP 10 apparatus and capillary tubes. Proton (^1^H) and carbon (^13 ^C) spectra were acquired using a Bruker Avance IIIHD Nanobay 400 MHz instrument that is equipped with a 5 mm BBO probe. Tetramethylsilane (TMS) was used as the internal standard and deuterated dimethyl sulfoxide (DMSO-d6) as the deuterated solvent. Chemical shifts (δ) and coupling constants (J) were reported in parts per million (ppm) and hertz (Hz) respectively. The internal standard (δ = 0 ppm) and DMSO-d6 (δ = 2.5 ppm) were used as the reference peaks. The multiplicities of the respective signals were indicated using the following abbreviations: s – singlet, d – doublet, t – triplet, m – multiplet. The atom numbering of the target compounds used for ^1^H NMR data are depicted on each respective compound found in the supplementary data.

#### 2.1.1. *Synthesis of 4–(3-Methyl-5-oxo-4*H*-pyrazol-1-yl)-*N*-(pyridine-4-ylmethyl)benzamide (3)*

The 4-(aminomethyl)pyridine moiety was conjugated to the carboxylic group of **1** via HATU activational chemistry. One equiv. edaravone-COOH (**1**) and four equiv. of *N,N-*diisopopylethylamine (DIPEA) was stirred at room temperature for 20 min. Thereafter, the carboxylic acid of **1** was activated using the HATU activational agent in a 1 equiv.:1.2 equiv. ratio in an appropriate quantity of dimethylformamide (DMF). The mixture was stirred at room temperature for 1 h and monitored using TLC (3 ethanol: 2 ethyl acetate: 4 diethyl ether). Once the reaction was complete, 4-(aminomethyl) pyridine (**2**) was added to the mixture and stirred under reflux at 40–50 °C for 1 h and monitored using TLC. Once the reaction was complete, toluene was added to the mixture in a ratio of 3 equiv. toluene: 1 equiv. DMF. The reaction was then rotary evaporated until just off dry. The mixture was left to precipitate out overnight in a refrigerator. Finally, the precipitate was filtered and washed with distilled water. The precipitate was placed in a vacuum oven and allowed to dry rendering the desired compound **3**.

Physical data: Yield: 72.52%; light pink solid; mp: 234 °C; Rf: 0.45; ^1^H NMR: (400 MHz, DMSO-d6), δ_H_: 9.10–9.13 (t, 1H, *J* = 5.60, 5.83, 11.43 Hz, H – 12), 8.50–8.51 (d, 2H, *J* = 5.88 Hz, H—16, 17), 7.95–7.98 (d, 2H, *J* = 8.68 Hz, H—6, 10), 7.85–7.87 (d, 2H, *J* = 8.64, H—7, 9). 7.30–7.32 (d, 2H, *J* = 5.76 Hz, H—15, 18), 4–49-4.51 (s, 2H, H—13), 2,13 (s, 3H, H—1); ^13 ^C NMR: (400 MHz, DMSO-d6): 166.36, 149.98, 149.15, 128.66, 122.62, 120.10, 42.21, 14.70; IR: (FT-IR, cm^−1^): 3217, 3035, 1713, 1637; MS: (HR-ESI^+^), [M + H^+^], *m/z*: calcd.: 309.1273, found: 309.1347.

#### 2.1.2. General procedure for the synthesis of compounds 5a–l

Compound **3** (1 equiv.) and 1.3 equiv. of the respective substituted benzyl bromide derivative (**4**) were dissolved and stirred under reflux, at 40–50 °C, in 5–6 ml of DMF. The compounds were monitored using TLC (3 ethanol: 2 ethyl acetate: 4 diethyl ether) for 4–6 h. Once the reaction was complete, 3 equiv. toluene: 1 equiv. DMF was added. The mixture was then rotary evaporated to dryness. Thereafter, 20 ml of diethyl ether was added to the dried mixture. The mixture was then left to precipitate out overnight in a refrigerator. Thereafter, if solid, the precipitate was filtered off and washed with diethyl ether. If the precipitate exhibited a waxy/oily appearance the mixture was diluted in a minimal amount of ethanol and transferred into a polytop. Finally, the precipitate or waxy/oily substance was dried, rendering the desired compounds **5a–l**.

##### 2.1.2.1. *1-Benzyl-4-({[4–(3-methyl-5-oxo-4*H*-pyrazol-1-yl)phenyl]foramido}methyl)pyridin-1-ium bromide (5a)*

Physical data: Yield: 95.29%; light grey solid; mp: 225 °C; ^1^H NMR: (400 MHz, DMSO-d6), δ_H_ 9.33–9.36 (t, 1H, *J* = 5.60, 5.72, 11.32 Hz H—12), 9.11–9.12 (d, 2H, *J* = 6.32 Hz, H—16, 17), 8.06–8.07 (d, 2H, *J* = 6.24 Hz, H—15, 18), 7.97–7.99 (d, 2H, *J* = 8.60 Hz, H—6, 10), 7.87–7.89 (d, 2H, *J* = 8.64 Hz, H—7, 9), 7.43–7.53 (m, 5H, H—21, 22, 23, 24, 25), 5.83 (s, 2H, H—19), 4.72–4.74 (d, 2H, *J* = 5.28 Hz, H—13), 2.13 (s, 3H, H—1); ^13 ^C NMR: (400 MHz, DMSO-d6): 166.68, 162.79,160.74, 159.84, 144.80, 134.87, 129.83, 129.72, 129.59, 129.23, 128.96, 128.84, 126.55, 119.36, 117.36, 63.14, 42.80, 17.19; IR: (FT-IR, cm^−1^): 3184, 3034, 1717,1639; MS: (HR-ESI^+^), [M-Br]^+^, *m/z*: calcd.: 399.1815, found: 399.1820.

##### 2.1.2.2. *1-[(2-Fluorophenyl)methyl]-4-({[4–(3-methyl-5-oxo-4*H*-pyrazol-1-yl)phenyl]foramido} methyl)pyridin-1-ium bromide (5b)*

Physical data: Yield: 97.17%; black solid; mp: 191 °C; ^1^H NMR: (400 MHz, DMSO-d6), δ_H_: 9.33–9.36 (t, 1H, *J* = 5.72, 5.83, 11.55 Hz, H—12), 9.03–9.04 (d, 2H, *J* = 6.44 Hz, H—16, 17), 8.06–8.08 (d, 2H, *J* = 6.52 Hz, H—15, 18), 7.87–8.00 (m, 4H, H—6, 7, 9, 10), 7.58–7.62 (t, 1H, *J* = 7.68, 7.56, 15.24, Hz, H—22), 7.51–7.55 (m, 1H, H—23), 7.34–7.35 (d, 1H, *J* = 1.88 Hz, H—24), 7.30–7.32 (d, 1H, *J* = 7.96 Hz, H—25), 5.93 (s, 2H, H—19), 4.74–4.75 (d, 2H, *J* = 5.40 Hz, H—13), 2.14 (s, 3H, H—1); ^13 ^C NMR: (400 MHz, DMSO-d6): 166.69, 162.78, 162.20, 161.08, 145.01, 132.65, 132.57, 131.96, 128.83, 126.53, 125.81, 125.78, 121.87, 121.72, 117.37, 116.62, 116.41, 57.83, 42.84; IR: (FT-IR, cm^−1^): 3201, 3034, 1717, 1638; MS: (HR-ESI^+^), [M-Br]^+^, *m/z*: calcd.: 417.1721found: 417.1719.

##### 2.1.2.3. *1-[(3-Fluorophenyl)methyl]-4-({[4–(3-methyl-5-oxo-4*H*-pyrazol-1-yl)phenyl]foramido} methyl)pyridin-1-ium bromide (5c)*

Physical data: Yield: 98.48%; brown solid; mp: 220 °C; ^1^H NMR: (400 MHz, DMSO-d6), δ_H_: 9.33–9.36 (t, 1H, *J* = 5.60, 5.83, 11.43 Hz, H—12), 9.11–9.13 (d, 2H, *J* = 6.64 Hz, H—16, 17), 8.07–8.08 (d, 2H, *J* = 6.56 Hz, H—15, 18), 7.87–8.00 (m, 4H, H—6, 7, 9, 10), 7.48–7.54 (m, 1H, H—23), 7.45–7.48 (d, 1H. *J* = 10.2, H—21), 7.37–7.39 (d, 1H, *J* = 7.72 Hz, H—25), 7.26–7.31 (m, 1H, H—24), 5.85 (s, 2H, H—19), 4.73–4.75 (d, 2H, *J* = 5.32, H—13), 2.14 (s,3H, H—1); ^13 ^C NMR: (400 MHz, DMSO-d6): 166.68, 163.88, 161.44, 160.89, 144.89, 137.26, 137.18, 131.91, 131.82, 128.84, 126.58, 125.48, 125.45, 119.39, 117.36, 116.87, 116.47, 62.34, 42.82, 17.18; IR: (FT-IR, cm^−1^): 3201, 3036, 1716, 1638; MS: (HR-ESI^+^), [M-Br]^+^, *m/z*: calcd.: 417.1721, found: 417.1732.

##### 2.1.2.4. *1-[(4-Fluorophenyl)methyl]-4-({[4–(3-methyl-5-oxo-4*H*-pyrazol-1-yl)phenyl]foramido} methyl)pyridin-1-ium bromide (5d)*

Physical data: Yield: 87.13%; light grey solid; mp: 215 °C; ^1^H NMR: (400 MHz, DMSO-d6), δ_H_: 9.32–9.35 (t, 1H, *J* = 5.60, 5.80, 11.43 HZ, H—12), 9.09–9.10 (d, 2H, *J* = 6.64 Hz, H—16, 17), 8.05–8.07 (d, 2H, *J* = 6.48 Hz, H—15, 18), 7.87–7.99 (m, 4H, H—6, 7, 9, 10), 7.61–7.64 (m, 2H, H—22, 25), 7.28–7.32 (t, 2H, *J* = 8.80, 17.60 Hz, H—21, 25), 5.81 (s, 2H, H—19), 4.72–4.73 (d, 2H, *J* = 5.44 Hz, H—13), 2.13 (s, 3H, H—1); ^13 ^C NMR: (400 MHz, DMSO-d6): 166.67, 164.27, 161.82, 160.76, 144.72, 131.93, 131.85, 131.09, 131.06, 128.94, 128.83, 126.55, 117.36, 116.74, 116.53, 62.29, 42.81, 17.19; IR: (FT-IR, cm^−1^): 3201, 3036, 1716, 1638; MS: (HR-ESI^+^), [M-Br]^+^, *m/z*: calcd.: 417.1721, found: 417.1729.

##### 2.1.2.5. *1-[(2-Chlorophenyl)methyl]-4-({[4–(3-methyl-5-oxo-4*H*-pyrazol-1-yl)phenyl]foramido} methyl)pyridin-1-ium bromide (5e)*

Physical data: Yield: 96.53%; black solid; mp: 211 °C; ^1^H NMR: (400 MHz, DMSO-d6), δ_H_: 9.36–9.38 (t, 1H, *J* = 5.60, 5.72, 11.32 Hz, H—12), 9.00–9.02 (d, 2H, *J* = 6.32 Hz, H—16, 17), 8.07–8.09 (d, 2H, *J* = 6.28 Hz, H—15, 18), 7.98–8.01 (d, 2H, *J* = 8.64 Hz, H—6, 10), 7.88–7.90 (d, 2H, *J* = 8.68 Hz, H—7, 9), 7.59–7.61 (d, 1H, *J* = 7.44 Hz, H—22), 7.47–7.54 (m, 3H, H—23, 24, 25), 5.97 (s, 2H, H—19), 4.76–4.77 (d, 2H, *J* = 5.32 Hz, H—13), 2.13 (s, 3H, H—1); ^13 ^C NMR: (400 MHz, DMSO-d6): 166.71, 162.79, 161.17, 145.19, 133.77, 132.09, 131.98, 130.61, 129.58, 128.96, 128.84, 128.67, 126.42, 119.38, 117.37, 61.11, 42.83, 17.81; IR: (FT-IR, cm^−1^): 3212, 3034, 1711, 1638; MS: (HR-ESI^+^), [M-Br]^+^, *m/z*: calcd.: 433.1425, found: 433.1441.

##### 2.1.2.6. *1-[(3-Chlorophenyl)methyl]-4-({[4–(3-methyl-5-oxo-4*H*-pyrazol-1-yl)phenyl]foramido} methyl)pyridin-1-ium bromide (5f)*

Physical data: Yield: 21.31%; black solid mp: 228 °C; ^1^H NMR: (400 MHz, DMSO-d6), δ_H_: 9.30–9.33 (t, 1H, *J* = 5.60, 5.83, 11.43 Hz, H—12), 9.09–9.11 (d, 2H, *J* = 6.6 Hz, H—16, 17), 8.05–8.07 (d, 2H, *J* = 6.44 Hz, H—14, 18), 7.96–7.98 (d, 2H, *J* = 8.72 Hz, H—6, 10), 7.87–7.89 (d, 2H, *J* = 8.76 Hz, H—7,9), 7.69 (s, 1H, H—21), 7.47–7.52 (t, 3H, *J* = 13.62, 6.22, 19,84 Hz, H—23, 24, 25), 5.81 (s, 2H, H—19), 4.73–4.74 (d, 2H, *J* = 5.40 Hz, H—13), 2.13 (s, 3H, H—1); ^13 ^C NMR: (400 MHz, DMSO-d6): 166.69, 160.90, 144. 89, 137.00, 134.16, 131.62, 129.83, 129.31, 128.83, 128.05, 126.60, 62.31, 42.84, 14.62; IR: (FT-IR, cm^−1^): 3213, 3035, 1710, 1639; MS: (HR-ESI^+^), [M-Br]^+^, *m/z*: calcd.:433.1425, found: 433.1430.

##### 2.1.2.7. *1-[(2-Bromophenyl)methyl]-4-({[4–(3-methyl-5-oxo-4*H*-pyrazol-1-yl)phenyl]foramido} methyl)pyridin-1-ium bromide (5g)*

Physical data: Yield: 94.2%; black solid; mp: 204 °C; ^1^H NMR: (400 MHz, DMSO-d6), δ_H_: 9.53–9.37 (t, 1H, *J* = 5.60, 5.83, 11.43 Hz, H—12), 8.98–9.00 (d, 2H, *J* = 6.52 Hz, H—16, 17), 8.07–8.09 (d, 2H, *J* = 6.40 Hz, H—15, 18), 7.87–8.00 (m, 4H, H—6, 7, 9, 10), 7.76–7.78 (d, 1H, *J* = 7.60 Hz, H—25), 7.49–7.53 (t, 1H, *J* = 7.06, 7.40, 14.46 Hz, H—22), 7.36–7.44 (m, 2H, H—23, 24), 5.94 (s, 2H, H—19), 4.76–4.78 (d, 2H, *J* = 5.44, H—13), 2.13 (s, 3H, H—1); ^13 ^C NMR: (400 MHz, DMSO-d6): 166.71, 162.77, 161.21, 145.24, 133.91, 133.56, 132.04, 131.98, 129.18, 128. 96, 128.84, 126.41, 123.98, 63.17, 42.84, 14.62; IR: (FT-IR, cm^−1^): 3217, 3034, 1711, 1638; MS: (HR-ESI^+^) [M-Br]^+^, *m/z*: calcd.: 477.0920, found: 477.0928.

##### 2.1.2.8. *1-[(3-Bromophenyl)methyl]-4-({[4–(3-methyl-5-oxo-4*H*-pyrazol-1-yl)phenyl]foramido} methyl)pyridin-1-ium bromide (5h)*

Physical data: Yield: 17.73%; dark grey solid; mp: 230 °C; ^1^H NMR: (400 MHz, DMSO-d6), δ_H_: 9.30–9.33 (t, 1H, *J* = 5.60, 5.72, 11.32 Hz, H—12), 9.09–9.10 (d, 2H, *J* = 6.4 Hz, H—16, 17), 8.05–8.07 (d, 2H, *J* = 6.32 Hz, H—15, 18), 7.96–7.98 (d, 2H, *J* = 8.60 Hz, H—6, 10), 7.87–7.89 (d, 2H, *J* = 8.64 Hz, H—7, 9), 7.83 (s, 1H, H—21), 7.63–7.65 (d, 1H, *J* = 8.00 Hz, H—23), 7.52–7.54 (d, 1H, *J* = 7.76 Hz, H—25), 7.39–7.54 (t, 1H, *J* = 7.74, 7.90, 15.64 Hz, H—24), 5.80 (s, 2H, H—19), 4.73–4.74 (s, 2H, *J* = 5.36 Hz, H—13), 2.13 (s, 3H, H—1); ^13 ^C NMR: (400 MHz, DMSO-d6): 166.69, 160.89, 144.88, 137.24, 132.74, 132.15, 131.86, 128.83, 128.43, 126.60, 122.72, 62.26, 42.84; IR: (FT-IR, cm^−1^): 3216, 3035, 1711, 1638; MS: (HR-ESI^+^), [M-Br]^+^, *m/z*: calcd.:477.0920, found: 477.0940.

##### 2.1.2.9. *1-[(4-Bromophenyl)methyl]-4-({[4–(3-methyl-5-oxo-4*H*-pyrazol-1-yl)phenyl]foramido} methyl)pyridin-1-ium bromide (5i)*

Physical data: Yield: 97.42%; black solid; mp: 190 °C; ^1^H NMR: (400 MHz, DMSO-d6), δ_H_: 9.33–9.36 (t, 1H, *J* = 5.95, 11.90 Hz, H—12), 9.08–9.10 (d, 2H, *J* = 6.80 Hz, H—16, 17), 8.06–8.07 (d, 2H, *J* = 6.64 Hz, H—15, 18), 7.95–7.99 (m, 2H, H—6, 10), 7.89–7.87 (d, 2H, *J* = 8.88 Hz, H—7, 9), 7.65–7.68 (d, 2H, *J* = 8.40 Hz, H—22, 24), 7.48–7.50 (d, 2H, *J* = 8.44 Hz, H—21, 25), 5.81 (s, 2H, H—8), 4.72–4.74 (d, 2H, *J* = 5.52 Hz, H—13), 2.13 (s, 3H, H—1); ^13 ^C NMR: (400 MHz, DMSO-d6): 166.67, 162.77, 160.85, 144.84, 134.11, 132.64, 131.58, 128.96, 128.84, 126.56, 123.34, 62.32, 42.80; IR: (FT-IR, cm^−1^): 3217, 3034, 1715, 1638; MS: (HR-ESI^+^), [M-Br]^+^, *m/z*: calcd.: 477.0920, found: 477.0943.

##### 2.1.2.10 *4-({[4–(3-Methyl-5-oxo-4*H*-pyrazol-1-yl)phenyl]foramido}methyl)-1–(2-methylphenyl)-pyridin-1-ium bromide (5j)*

Physical data: Yield: 21.75%; light grey solid; mp: 194 °C; ^1^H NMR: (400 MHz, DMSO-d6), δ_H_: 9.34–9.37 (t, 1H, *J* = 5.60, 5.83, 11.43 Hz, H—12), 8.93–8.95 (d, 2H, *J* = 6.64 Hz, H—16, 17), 8.06–8.08 (d, 2H, *J* = 6.56 Hz, H—15, 18), 7.98–8.00 (d, 2H, *J* = 8.80 Hz, H—6, 10), 7.87–7.89 (d, 2H, *J* = 8.80 Hz, H—7, 9), 7.20–7.37 (m, 3H, H—23, 24, 25), 7.12–7.14 (d, 1H, *J* = 7.52 Hz, H—26), 5.88 (s, 2H, H—19), 4.75–4.76 (d, 2H, *J* = 5.40 Hz, H—13), 2.29 (s, 3H, H—22), 2.13 (s, 3H, H—1); ^13 ^C NMR: (400 MHz, DMSO-d6): 166.70, 162.77, 160.81, 144.98, 137.38, 132.82, 131.43, 129.87, 129.57, 128.95, 128.83, 127.20, 126.49, 117.37, 61.36, 42.81, 19.23, 17.19; IR: (FT-IR, cm^−1^): 3215, 3035, 1715, 1639; MS: (HR-ESI^+^), [M-Br]^+^, *m/z*: calcd.: 413.1972, found: 413.1968.

##### 2.1.2.11. *4-({[4–(3-Methyl-5-oxo-4*H*-pyrazol-1-yl)phenyl]foramido}methyl)-1–(3-methylphenyl)-pyridin-1-ium bromide (5k)*

Physical data: Yield: 98.3%; black powder; mp: 212 °C; ^1^H NMR: (400 MHz, DMSO-d6), δ_H_: 9.31–9.34 (t, 1H, *J* = 5.60, 5.83, 11.43 Hz, H—12), 9.08–9.09 (d, 2H, *J* = 6.64 Hz, H—16, 17), 8.04–8.06 (d, 2H, *J* = 6.52 Hz, H—15, 18), 7.96–7.98 (d, 2H, *J* = 8.88 Hz, H—6, 10), 7.87–7.89 (d, 2H, *J* = 8.80 Hz, H—7, 9), 7.33–7.35 (d, 1H, *J* = 5.24 Hz, H—21), 7.29–7.31 (t, 2H, *J* = 2.35, 7.40, 9.75 Hz, H—24, 25), 7.23–7.24 (d, 1H, *J* = 6.84 Hz, H—26), 5.77 (s, 2H, H—19), 4.72–4.73 (d, 2H, *J* = 5.44 Hz, H—13), 2.30 (s, 3H, H—23), 2.13 (s, 3H, H—1); ^13 ^C NMR: (400 MHz, DMSO-d6): 166.68, 160.68, 144,76, 139.10, 134. 76, 130.46, 129.75, 129.63, 128.83, 126.52, 126.32, 63.19, 42.81, 21.38, 17.1; IR: (FT-IR, cm^−1^): 3208, 3034,1716, 1638; MS: (HR-ESI^+^), [M-Br]^+^, *m/z*: calcd.:413.1972, found: 413.1973.

##### 2.1.2.12. *4-({[4–(3-Methyl-5-oxo-4*H*-pyrazol-1-yl)phenyl]foramido}methyl)-1–(4-methylphenyl)-pyridin-1-ium bromide (5l)*

Physical data: Yield: 60.13%; light grey solid; mp: 230 °C; ^1^H NMR: (400 MHz, DMSO-d6), δ_H_: 9.30–9.33 (t, 1H, *J* = 5.60, 5.83, 11.43 Hz, H—12), 9.06–9.08 (d, 2H, *J* = 6.6 Hz, H—167, 17), 8.03–8.05 (d, 2H, *J* = 6.52 Hz, H—15, 18), 7.96–7.98 (d, 2H, *J* = 8.68 Hz, H—6, 10), 7.86–7.89 (d, 2H, *J* = 8.76 Hz, H—7, 9), 7.41–7.43 (d, 2H, *J* = 7.96 Hz, H—21, 26) 7.24–7.26 (d, 2H, *J* = 7.88 Hz, H—22, 25), 5.76 (s, 2H, H—19), 4.71–4.72 (d, 2H, *J* = 5.44 Hz, H—13), 2.29 (s, 3H, H—24), 2.13 (s, 3H, H—1); ^13 ^C NMR: (400 MHz, DMSO-d6):166.67, 160.64, 144.67, 139.89, 131.89, 130.24, 129.29, 128.82, 126.50, 63.01, 42.81, 21.21, 17.1; IR: (FT-IR, cm^−1^): 3224, 3032, 1714, 1638; MS: (HR-ESI^+^), [M-Br]^+^, *m/z*: calcd.: 413.1972, found: 413.1974.

### 2.2. Ache molecular docking studies

Molecular Operating Environment (MOE) 2018.10 software package^41^ was employed to predict the interactions and binding modes of the novel compounds within the active site of *Tc*AChE. The co-crystallised structure of *Tc*AChE with donepezil (PDB accession code: 1EVE) was acquired from the Protein Data Bank (PDB)^38^. The following protocol was employed, as previously described^42^, to simulate the orientation and binding interactions of the test compounds. Firstly, the test compounds were drawn using ChemSketch v2019.2.1 and saved as a mol files. Secondly, the enzyme structures were inspected for missing atoms, bonds and contacts. Thirdly, partial charges and hydrogens were added using MOEs’ protonate 3 D application. Fourthly, the ligands were assembled employing the builder module in MOE and energy minimisation (MMFF94x) was performed. Thereafter, the ligands were docked, using the MOEdock application, within the AChE active site. Finally, the retained best poses, as per their binding affinity scores, were inspected visually and analysis of the interactions within the active aromatic gorge of AChE was conducted. To determine the accuracy of this docking protocol, the co-crystallised ligand, was re-docked into the AChE active site. This procedure was repeated three times and the best ranked solution exhibited an RMSD value of less than 2.0 Å from the position of the co-crystallised ligand. The RMSD value in this case is smaller than 2.0 Å indicating that the docking protocol is capable of accurately predicting the binding orientation of the co-crystallised ligand^43^. This protocol was thus deemed to be suitable for the docking of inhibitors into the active site model of AChE.

### 2.3. Pharmacological evaluation studies

#### 2.3.1. Cholinesterase inhibition assay

A modified Ellman’s method was employed to determine the ChE inhibitory activities of the synthesised compounds^44^. *ee*AChE, eqBuChE, 5,5′-dithiobis-(2-nitrobenzoic acid) (DTNB, commonly known as Ellman’s reagent), *S*-butrylthiocholine iodide (BTCI), acetylthiocholine iodide (ATCI) and donepezil were purchased from Sigma-Aldrich^®^. *ee*AChE, *eq*BuChE, DTNB, BTCI and ATCI were diluted with a buffer solution (tris hydrochloride (50 mM), pH 8). Each well of a 96 well plate, contained the following; 148 µL of 1.5 mM DTNB, 50 µL of either 0.22 U/ml *ee*AChE or 0.12 U/ml *eq*BuChE and 2 µL of either test compound, control (donepezil) or blank [dimethyl sulfoxide (DMSO)]. The test compounds and control were dissolved in DMSO and added to the well to yield various concentrations (1000 µM, 100 µM, 10 µM, 1 µM, 0.1 µM and 0.01 µM). The quantity of DMSO per well accumulated to below 0.01%^45^. Each concentration of test compound and control was conducted in triplicate to ensure consistent results. The well-plates were then incubated at 25 °C for 10 min. Thereafter, 30 µL of either the ATCI or BTCI substrate was added to each well. The plate was then placed inside a Rayto 6500 spectrometer and the absorbance was read at 405 nm every 45 s for 5 min. The percentage activity was calculated using the following equation: [(absorbance of blank − absorbance of test compounds)/absorbance of blank × 100)]. All data analysis was conducted on GraphPad Prism version 8.2.1 for Mac OS, GraphPad Software (San Diego, CA, USA)

#### 2.3.2. Antioxidant assay

Antioxidant activity was studied with the DPPH^+^ free radical scavenging assay^46^^,^^47^. DPPH^+^ and trolox were purchased from Sigma-Aldrich^®^. The following method was employed, as previously described^48^, to determine the antioxidant activity of the test compounds. Each well of a 96 well plate, contained the following: 180 µL of 0.12 mM DPPH^+^ dissolved in methanol and 20 µL of either test compound, control (trolox) or blank (DMSO). The test compounds and control were dissolved in DMSO and added to the well to yield various concentrations (1000 µM, 100 µM, 10 µM, 1 µM and 0.1 µM). Each concentration of test compound and control was conducted in triplicate to ensure consistent results. The well plate was incubated at 25 °C for 30 min within a dark space. A change of colour from dark purple to light yellow was observed. The quantity of DMSO per well accumulated to below 0.01%^45^. The plate was then placed inside a Rayto 6500 spectrometer and the absorbance was read at 517 nm three times to ensure statistical viability. The percentage activity was calculated using the following equation: [(absorbance of blank − absorbance of test compounds)/absorbance of blank × 100)]. All data analysis was conducted on GraphPad Prism version 8.2.1 for Mac OS, GraphPad Software (San Diego, California, USA).

### 2.4. In silico blood–brain permeability predictions

An *in silico* model was used to determine the blood–barrier permeability of the synthesised compounds. The BBB predictor used can be found on an intergrade cloud computing server called *AlzPlatform*^49^. The BBB predictor was designed to determine whether a ligand is permeable across the blood brain barrier (BBB+) or not (BBB−). The BBB predictor was developed by applying the LiCABEDS and support vector machine (SVM) algorithms on four types of fingerprints of 1593 reported compounds^50–52^. The BBB predictor software employed is available at http://www.cbligand.org/BBB/.

## 3. Results and discussion

Previous studies have shown that molecules that contain the *N*-benzyl pyridinium moiety interact with the CAS of the AChE enzyme^36^^,^^37^^,^^40^. Therefore, to determine if the edaravone portion of the hybrid molecules would exhibit the proposed interactions with the PAS as well as potential interactions exhibited by the *N-*benzyl pyridinium moiety with the CAS, molecular docking studies were performed. The molecular docking studies were conducted using the Molecular Operating Environment (MOE) 2018.10 software package^53^. The co-crystallised structure of *Tc*AChE with donepezil (PDB accession code: 1EVE)^38^ was utilised to establish the starting model for AChE active site docking^54^. The findings from these studies were then used to rationalise the synthesis and evaluation of these compounds as potential MTDLs.

**Figure 3.**
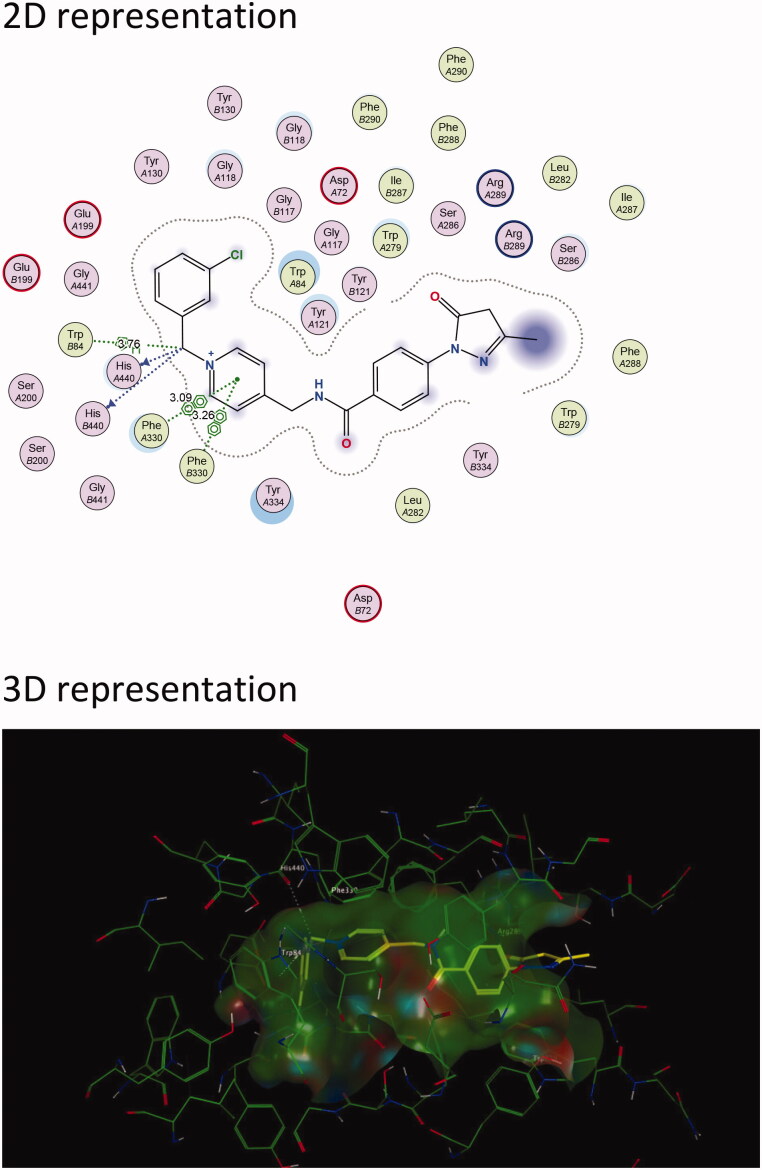
The active site cavity of AChE exhibiting the binding and interactions of representative compound **5f** (Scheme 1). (a) Two-dimensional (2D) representation of the docked compound **5f**. The close proximity of the Arg 289 residue to edaravone’s pyrazoline ring can be observed. (b) Three-dimensional (3D) representation of the docked compound **5f**, showing the orientation and positing of **5f** within the AChE active site cavity.

**Figure 4.**
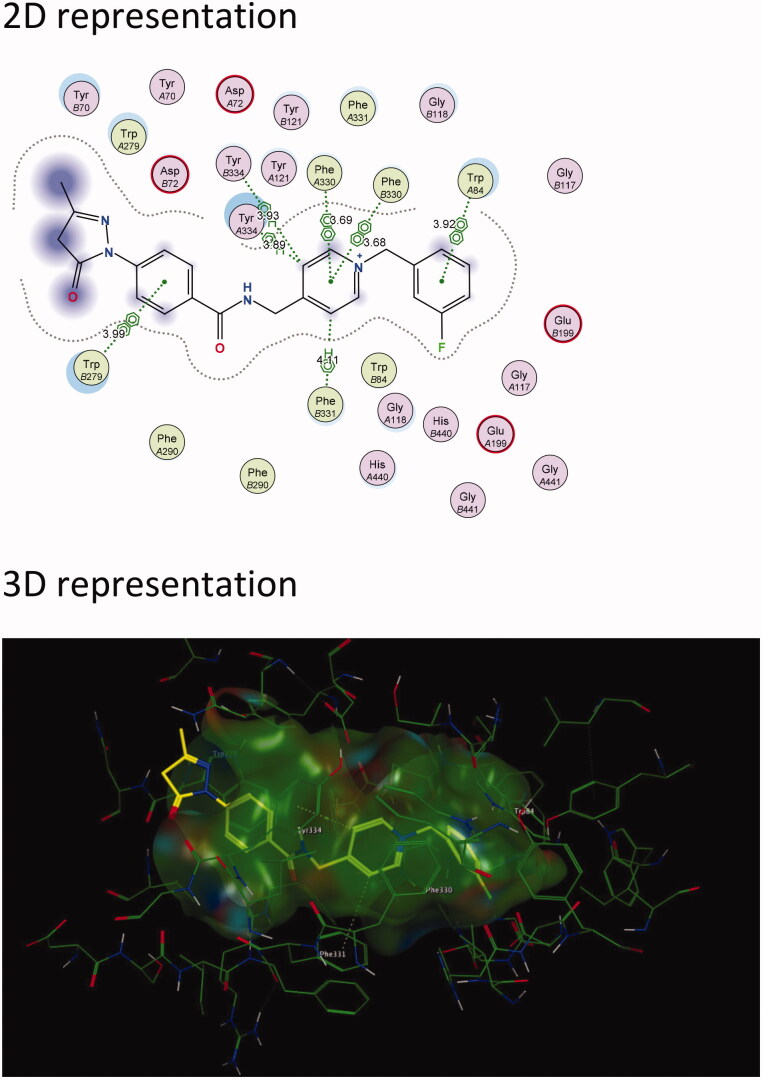
The active site cavity of AChE exhibiting the binding and interactions of representative compound **5c** (Scheme 1). (a) 2 D representation of the docked compound **5c**. Interactions with Trp 279 (PAS) and Trp 84 (CAS) can be observed. (b) 3D representation of the docked compound **5c**, showing the orientation and positing of **5c** within the AChE active site cavity.

Results show that the majority of the hybrid compounds exhibit potential interactions with important conserved residues within the PAS site (Figures 3 and 4, and Supplementary Material). In general, the compounds interacted with a combination of important residues within the PAS, which included Trp 279 and Tyr 334^40^^,^^55^. The compounds were also observed in close proximity to Arg 289. The Arg 289 residue is found in *site I* that is part of one of the four putative binding sites within the PAS that was shown to play an important role in Aβ formation^38^. It has also previously been reported that Trp 279 plays a role in Aβ formation^54^^,^^56^^,^^57^. These findings support the hypothesis that the edaravone-*N*-benzyl pyridinium hybrid compounds could inhibit AChE and significantly reduce the formation of AChE induced Aβ plaques.

**Scheme 1.**
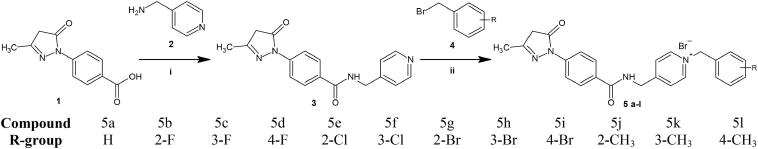
Synthesis pathway of the edaravone-*N*-benzyl pyridinium derivatives **5a–5l**. Reagents and conditions: (i) HATU, DMF, DIPEA, 2 h, stirring under reflux. (ii) DMF, stirring under reflux, 4–6 h at 40–50 °C. (1) Edaravone-COOH, (2) 4-(aminomethyl) pyridine, (4) R-benzyl bromide derivatives.

In general, the hybrid compounds exhibited interactions with the AChE mid-gorge recognition site residues Phe 330 and Phe 331 (Figures 3 and 4, and Supplementary Material)^55^. These interactions are π–π interactions with the pyridinium ring and the carbon linker between the pyridinium ring and amide. It has also previously been observed that the interaction between the pyridinium ring and Phe 330 leads to stabilisation in the orientation of a compound. This in turn, increases the probability for an interaction with Trp 279 within the PAS^37^. The benzyl moiety of the hybrid molecules was mainly found to interact with the residue Trp 84 within the CAS^55^. Trp 84 is a key residue found within the CAS and is responsible for the molecular recognition of cationic substrates in cholinesterases. Previous studies have shown that the replacement of residue Trp 84 with alanine caused a drastic decrease in human AChE’s ability to hydrolyse acetylthiocholine^57^. Interactions were also observed with His 440, a residue of the catalytic triad found in the CAS and with Glu119, found in the oxyanion site of the CAS. All these residues play a variable role in the inhibitory activity of AChE^55^. From these results, it is clear that the majority of the hybrid compounds exhibit similar orientational and positional conformations within the AChE active site. The only exception was found in compound **5i** (Scheme 1 and Supplementary Material) in that its most stable conformations were flipped within TcAChE active site. The pyrazoline of the edaravone moiety in **5i** interacted with Trp 84 through H–π stacking and the pyridinium ring interacted with Trp 279 through π–π stacking as well as H–π stacking. The intermediate **3**, lacking the benzyl moiety (Scheme 1 and Supplementary Material), interacts with residues Phe 288 and Trp 84 present in the mid-gorge and CAS respectively, and is not able to travers into the PAS of the AChE active site. This indicates the potential importance of the benzyl moiety for optimal dual site binding AChE inhibitory activity in these compounds. These results therefore indicate that the docked compounds could exhibited the potential to act as dual-site AChE inhibitors.

The synthesis of the novel MTDLs was carried out in two steps (Scheme 1). Firstly, an amide intermediate (**3**) was formed by reacting edaravone-COOH (**1**) with 4-(aminomethyl) pyridine (**2**) *via* 1-[bis(dimethylamino)methylene]-1*H*-1,2,3-triazolo[4,5-*b*]pyridinium-3-oxid hexafluoro-phosphate (HATU) activational chemistry. Thereafter, the final edaravone-*N*-benzyl pyridinium derivatives (**5a**–**5l**), containing the pyridinium moiety, were synthesised *via N*-benzylation of compound **3** with benzyl bromide containing various substitutions (H, Br, F, Cl or CH_3_) at different positions of the benzene ring.

Analytical instruments and techniques, described in the experimental section, were employed for structural elucidation of the synthesised compounds. All of the protons and carbons of the aromatic groups, CH_2_ groups and CH_3_ moiety of the pyrazoline ring, were observed for all the final compounds and exhibited similar peaks on the ^1^H- and ^13 ^C-NMR spectra (Supplementary Material). The only variance in NMR peaks were as a result of the different functional group substitutions on the benzyl moiety. The formation of the positively charged nitrogen of the pyridinium group in **5a**–**5l**, can be observed by a shift of the pyridine ^1^H NMR doublet peaks from ∼ 8.5 ppm and ∼7.3 ppm to ∼9.1 ppm and ∼8.0 ppm respectively when compared to the NMR of intermediate **3**. HRMS also confirmed the molecular masses and identity of the synthesised compounds. Refer to the supplementary material for all NMR spectra and a complete discussion on NMR and HRMS results.

**Figure 5.**
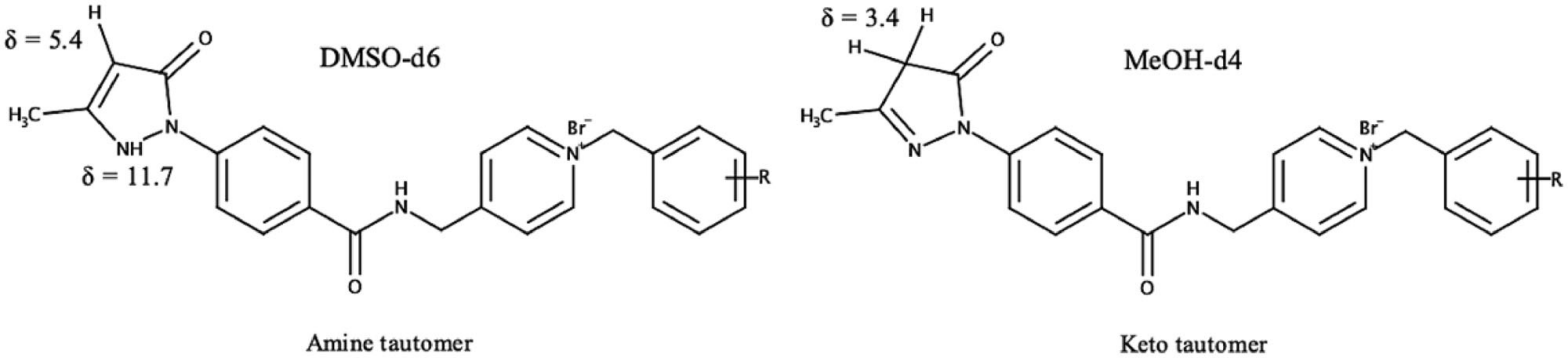
Two major tautomeric forms of the edaravone-*N-*benzyl pyridinium hybrid compounds and their respective ^1^H NMR chemical tautomeric shifts in deuterated solvents.

**Figure 6.**
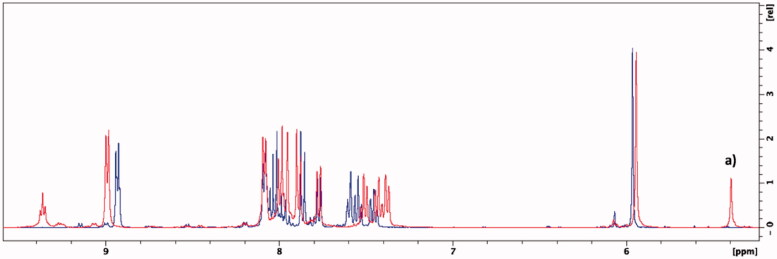
Overlaid ^1^H NMR spectra of compound **5g** in methanol-d4 (blue) and DMSO-d6 (red). (a) Singlet that represents the CH-group of the amine tautomer.

Edaravone is known to exist in different neutral tautomeric forms^58^. In all ^1^H NMR spectra where DMSO-d6 was used additional peaks, visible at δ = 5.4 ppm and 11.7 ppm, were observed suggesting that the novel synthesised compounds may also exist in different tautomeric states (Figures 5 and 6, and Supplementary Material). The peaks, at δ = 5.4 ppm and δ = 11.7 ppm, were found to belong to the respective CH-group and NH-group of the amine tautomer portion of the edaravone of the synthesised compounds^59^. In previous research, it has been found that the amine tautomer of edaravone may be more stable in aprotic polar solvents such as DMSO compared to the keto tautomer^59^^,^^60^. The NMR experiment was repeated using the protic polar solvent, methanol-d4. Using this solvent system, the keto tautomer was found to be more stable as no peaks were present at δ = 5.4 ppm and δ = 11.7 ppm (Figure 6). This finding is in accordance to previous research as it has been found that the enol and keto tautomeric forms are both found within this solvent system. In addition, the keto tautomer was found to be the predominant of the two tautomeric forms in methanol^61^. The enol tautomer ^1^H NMR peak for the CH-group at δ = ∼6.2 ppm^62^ was not present and further confirms the presence of the keto tautomer within the methanol-d4 solvent system. The formation of the edaravone tautomers are dependent on certain solvation effects and electrostatic interactions between the solvent and molecule and further studies are to be conducted to explore the tautomeric nature of these hybrid molecules. Based on the data in this study, the keto tautomer of these hybrid molecules seems to be more stable in polar protic solvents whereas its amine tautomeric form is more stable in polar aprotic solvents. As the biological evaluations on these compounds were carried out in protic environments it is expected that the keto tautomer will be the predominant form present. The effects of the different tautomers on the biological profile of these compounds should therefore be taken into consideration for future pharmaceutical development.

The inhibitory activities of the synthesised compounds were evaluated using eeAChE (*electric eel*) and eqBuChE (*equine serum*) according to a modified method of Ellman^44^. Donepezil, a known potent ChE inhibitor, was used as the reference compound for both assays. This reference compound was chosen as the structure of donepezil contains similarities to the *N*-benzyl pyridinium moiety of the synthesised compounds. Compounds **5b–5g** exhibited some of the best inhibitory activities (Table 1). These compounds have either a fluorine or chlorine substituted at various positions of the benzyl moiety and exhibited IC_50_ values between 1.2 and 4.6 µM. It can be deduced that smaller and more electronegative substitutions, such as fluorine or chlorine, in comparison to larger substitutions, such as bromine (**5h**–**5j**) or methyl (**5g**–**5i**), is preferred as it results in superior inhibitory activity (Table 1). The majority of the final compounds also exhibited improved activity compared to the unsubstituted benzyl moiety of **5a**.

**Table 1.** *In silico* BBB predictions and IC_50_ values (µM) of the test compounds and controls for *ee*AChE, *eq*BuChE, and DPPH^+^.

Compound	R….	AChE	BuChE	SI AChE^a^	DPPH^+^	BBB prediction^b^
**3**	–	>100	>1000	n.d.	12.5	0.108
**5a**	H	30	>1000	>33	22.9	0.081
**5b**	2-F	1.2	>1000	>83	28.8	0.068
**5c**	3-F	4.6	926	201	25	0.064
**5d**	4-F	3.6	>1000	>278	11.5	0.064
**5e**	2-Cl	1.9	890	468	13.8	0.060
**5f**	3-Cl	3.3	891	270	13.1	0.055
**5g**	2-Br	3.5	160	46	19	0.055
**5h**	3-Br	>100 µM	>1000	n.d.	13.3	0.062
**5i**	4-Br	11.5	>1000	>87	18.1	0.056
**5j**	2-CH_3_	19.9	630	32	9.5	0.056
**5k**	3-CH_3_	69.1	>1000	>15	27.5	0.080
**5l**	4-CH_3_	95.5	>1000	>11	14.5	0.077
**1**	–	n.d.	n.d.	n.d.	45.7	–
Donepezil^c^	–	0.006	7.14	1252	–	–
Trolox	–	n.d.	n.d.	n.d.	13.1	–
Edaravone^d^	–	–	–	–	–	–

^a^AChE selectivity index = IC_50_(*eq*BuChE)/IC_50_(*ee*AChE).

^b^A value higher than 0.02 is predicted to cross the BBB using *AlzPlatform’s* intergrade cloud computing server^49^^,^^63^.

^c^IC_50_ values of donepezil reported by reference [64].

^d^IC_50_ values of edaravone reported by reference [65].

n.d.: not determined.

In addition, all the compounds with substituents in the ortho position of the benzyl ring (**5b**, **5e**, **5g**, **5j**) exhibited the highest inhibitory activities when compared to their meta and para counterparts. This finding is similar to that reported in previous studies^37^^,^^66^. The superior activity observed for **5a–5l** when compared to intermediate **3**, could be due to the increase in the length of the molecule. Molecular modelling corresponds with this observation in that **3** is too short to interact with both the PAS and CAS of the AChE active site. This also confirms the importance of the *N*-benzyl pyridinium group for optimal AChE inhibitory activity within these compounds.

Compounds **5a–5l** also exhibited highly selective AChE inhibitory activity over BuChE (Table 1, SI:11 – >278). It can be speculated that the poor BuChE inhibitory results exhibited by all the compounds are due to the phenyl ring of edaravone and the pyridinium moiety exhibiting π–π interactions with the aromatic residues of AChE but are not able to exhibit similar interactions with BuChE’s aliphatic and/or aromatic residues^67^^,^^68^. The *N*-benzyl pyridinium moiety presents a similar structure to donepezil’s benzyl-pyridine moiety^69^. Therefore, the high selectivity of the hybrid compounds for AChE compared to BuChE was expected. Selectivity to AChE is advantageous as it has been shown to result in lower incidences of cholinergic side effects and wider therapeutic indices compared to non-selective cholinesterase inhibitors^70^. Most of the hybrid compounds exhibited proficient AChE inhibitory activity when compared to previously designed, AChE acting MTDLs^30^.

The 2,2-diphenyl-1-picrylhydrazyl (DPPH^+^) assay was employed to determine the antioxidant ability of the synthesised compounds. Trolox, a known potent antioxidant, was used as the reference compounds for this assay^71^. Compounds **3**, **5d–j** and **5l** (IC_50_: 9.5–19 µM) exhibited similar or greater antioxidant activity when compared to the control, Trolox (IC_50_ = 13.1 µM). In addition, these compounds retained the antioxidant activity of edaravone (IC_50_ = 4.7 µM)^65^. Compound **5j** had the best IC_50_ of 9.5 µM. The rest of the compounds within the series exhibited greater antioxidant activity compared to **1**. These results correspond with previous literature in that a large lipophilic substitution on the 4-position of the phenyl ring of the edarvone moiety improves its antioxidant activity when compared to a smaller carboxylic group on the 4-position of the phenyl ring of the edaravone structure^58^^,^^72^^,^^73^.

Potential AD therapeutic agents are required to cross the blood–brain barrier (BBB) to act in the CNS. Therefore, an *in silico* model^49^^,^^63^ was used to determine the blood–barrier permeability of the synthesised compounds. The BBB permeability prediction score represents the compounds ability to cross the BBB. A threshold score of over 0.02 is considered that the compounds are BBB permeable (BBB+) and a score below 0.02 is considered that the compounds are BBB impermeable (BBB−). The results are shown in Table 1. All the compounds exhibit scores of above 0.02 and are therefore predicted to effectively cross the BBB.

## 4. Conclusions

The main goal of this study was to design and synthesise a novel series of multi-target directed edaravone-*N*-benzyl pyridinium hybrid compounds that exhibit cholinesterase inhibitory activity and significant antioxidant ability. The molecular modelling results showed that these compounds should be able to form significant interactions within the PAS and CAS of the AChE active site, which in turn should lead to notable inhibitory activities. The *in vitro* cholinesterase results indicated excellent selective AChE inhibitory activity. Compounds **5b–g** demonstrated the best AChE inhibitory activity showing that smaller substitutions, e.g. fluorine and chlorine, especially in the ortho benzyl position is important for AChE inhibitory activity. Compounds **5d–j** and **5l** exhibited potent antioxidant activity that was comparable to trolox and edaravone. *In silico* blood–brain barrier evaluations predicted that all these hybrid compounds should cross the BBB. Compound **5d–g** presented as the most promising MTDL candidates against AD. These compounds exhibited excellent selective AChE inhibitory activities (IC_50_: 1.9–3.6 µM), promising antioxidant abilities (IC_50_: 11.5–19 µM) and are predicted to cross the BBB. Further exploration of these compounds’ abilities to exhibit Aβ inhibitory activity, neuroprotection and their pharmacokinetic- and toxicity profiles are recommended.

## Supplementary Material

Supplemental Material
