# Supplementary material for: Design, synthesis and biological evaluation of edaravone derivatives bearing the N-benzyl pyridinium moiety as multifunctional anti-Alzheimer’s agents
Source: J Enzyme Inhib Med Chem. 2020 Aug 11;35(1):1596–605. doi: 10.1080/14756366.2020.1801673 (PMC7470113; doi:10.1080/14756366.2020.1801673)

Supplementary Material

Design, synthesis and biological evaluation of edaravone derivatives bearing the *N*-benzyl pyridinium moiety as multifunctional anti-Alzheimer's agents

Luke S. Zondagh[1], Sarel F. Malan[1], Jacques Joubert[1]

^{1}H and ^{13}C NMR discussion

The NMR peaks of compound **3** and **5a** – **5l** were observed on all ^{1}H NMR spectra except for the CH_2 within the pyrazoline ring of edaravone. Previous research has shown that this CH_2 peak is found at δ = ~ 3.4 ppm. Therefore, the peak is overlapped by the DMSO-d6 water peak that is found at the same chemical shift. The rest of the ^{1}H NMR peaks correlate to the proposed final compounds and HRMS confirmed their molecular masses.

Edaravone has three neutral tautomeric forms. In all ^{1}H NMR spectra a peak is visible at δ = ~ 5.4 ppm. This peak belongs to the amine tautomer of the edaravone derivative when dissolved in DMSO-d6. The NH peak of the amine tautomer at δ = ~ 11.7 ppm, was observed as a broad peak with an integration around 0.1. A further test was to determine which tautomeric form was most stable in MeOH-d4. It was discovered that the keto tautomer was more stable in methanol-d4 and no peak was present at δ = 5.4 ppm or δ = 11.7 ppm. The CH_2 group of the pyrazoline ring of edaravone is overlapped by the methanol-d4 solvent peak on the ^{1}H NMR spectra (δ = 3.3 ppm) and was therefore not observed. The experiment was also attempted in $CDCl_3$; however, these compounds are not soluble in this solvent system.

The NH peak of the amide linker is present on all DMSO-d6 ^{1}H NMR spectra at δ = ~9.3 ppm. The integration of the NH peak did not account for one proton. The reason is that the proton of NH is a labile proton. The integration is affected by possible hydrogen bonding taking place between the amide and water found in the DMSO-d6. To confirm that the NH of the amide was a labile, an ^{1}H NMR experiment was conducted in methanol-d4. Methanol-d4 is a protic solvent and therefore MeOD/H exchanges with the deuterium atom of MeOD and becomes ND. Deuterium resonates at 61 MHz on a 400 MHz instrument where as 1H resonates at 400MHz. Therefore, the ND is in a different window and cannot be observed on the 400 MHz spectra. As DMSO-d6 is an aprotic solvent this H exchange does not take place. A disappearance of the NH peak, of the amide linker, was observed when the ^{1}H NMR was conducted in methanol-d4. It was further confirmed

with HSQC NMR as no carbon correlated with this peak. Therefore, the presence of the secondary amine of the amide was confirmed.

^{13}C NMR was also conducted and it was observed that the methyl peak at around δ = 17.1 ppm was not present on all ^{13}C NMR spectra. It was found that when examining the HSQC NMR spectra two ^{13}C peaks at δ = 17.1 ppm and δ = 14.6 ppm correlated with the methyl peak on the ^{1}H NMR at a δ = 2.1 ppm. The two carbons correlating to the one proton could be caused by the spinning of the methyl group. Thus, this shows that a methyl group is present. ^{1}H NMR as well as MS further confirms the above reasoning.

HRMS discussion

The molecular masses were confirmed with HRMS. Compound **3** exhibited a $[M+H]^+$ peak. Compounds **5a-l** all exhibited $[M-Br]^+$ peaks. The bromine is pulled away from the structure and can be observed in spectrum at m/z= 79.0220 amu. It was also be observed that both chlorine (**5e-f**) and bromide (**5g-i**) substituted compounds have a $[M+2H]^+$ peaks. This is due to isotopic abundance of these two halogens. Bromine comes in two isotopes that have respective molecular weights of 79 g/mol and 81 g/mol. The natural abundance of these two isotopes are 50.69% and 49.31% respectively. Therefore, two peaks on the MS spectra of **5g-i** that are approximately the same peak height and 2 amu apart is observed. Chlorine also has two isotopes that have respective molecular weights of 35 g/mol and 37 g/mol. The natural abundance of these isotopes is 75.78% and 24.22% respectively. Therefore, two peaks on the MS spectra of **5e-f** with a peak height of 1 (^{35}Cl): 0.33 (^{37}Cl) and 2 g/mol apart is observed.

Molecular docking studies

Table 1: 3D and 2D representations and interactions of compound **3** and **5a**-**5l** docked within *Tc*AChE active site.

Comp.	Ligand interactions with aromatic residues (2D)	Compounds docked within the *Tc*AChE active site (3D)
**3**	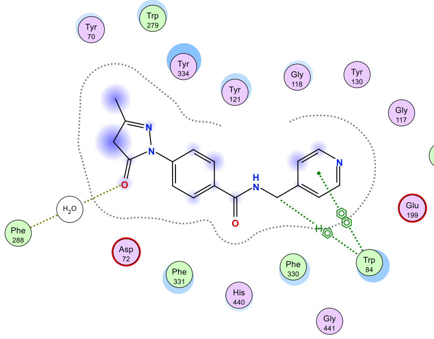	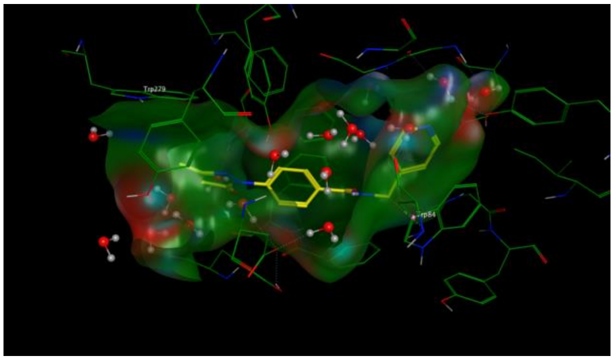
**5a**	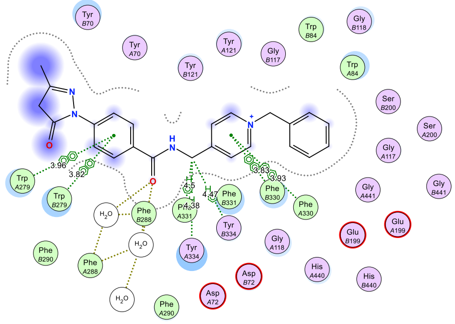	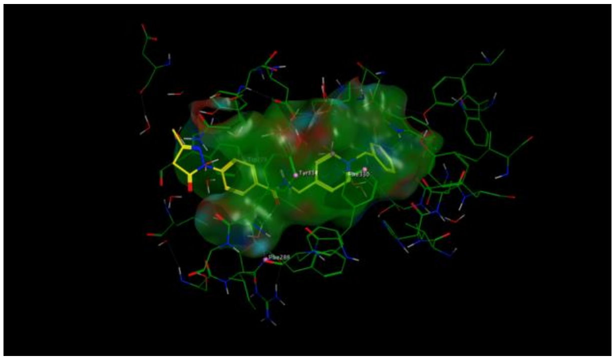
**5b**	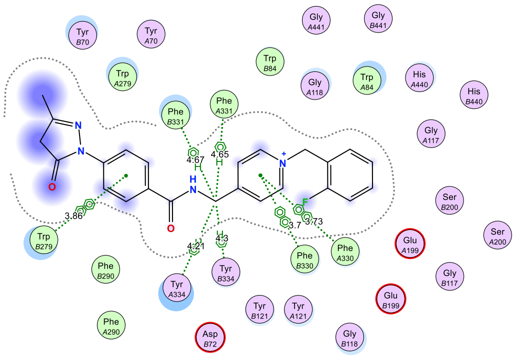	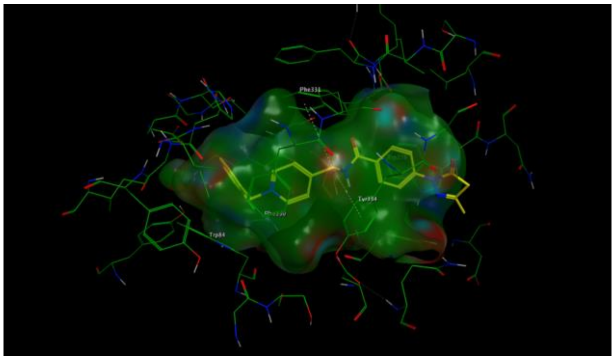
**5c**	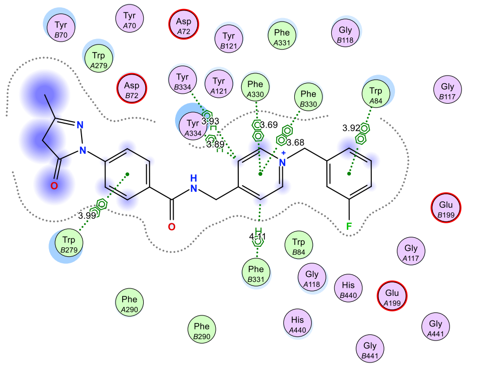	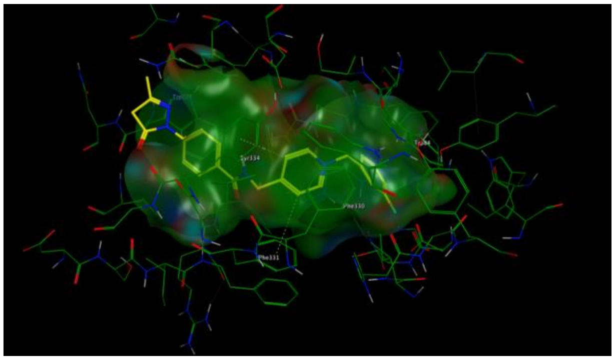

5d

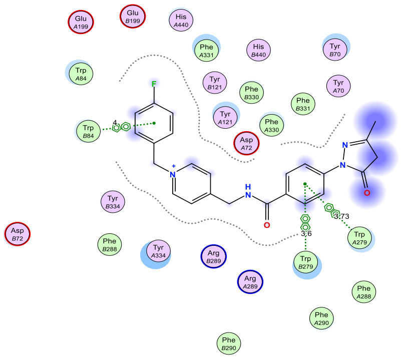
Glu A199
Glu B199
His A440
Phe A331
His B440
Tyr B70
Trp A84
Tyr B121
Phe B330
Tyr A70
Phe B331
Trp B84
Tyr A121
Phe A330
Asp A72
Tyr B334
Asp B72
Phe B288
Tyr A334
Arg B289
3.6
3.73
Trp A279
Trp B279
Arg A289
Phe A288
Phe A290
Phe B290

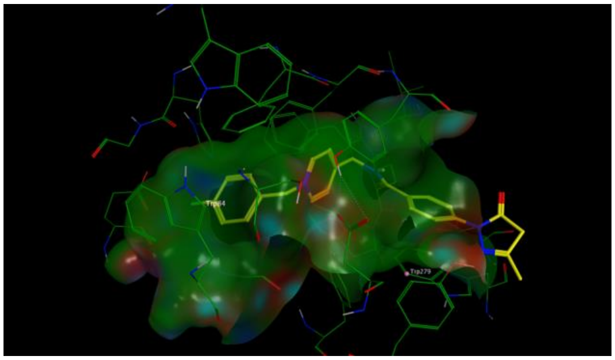

5e

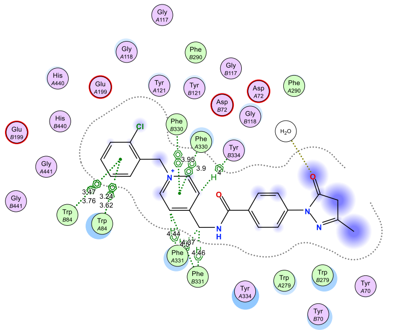
Gly A117
Gly A118
Phe B290
Gly B117
Phe A290
His A440
Glu A199
Tyr A121
Tyr B121
Asp A72
Asp B72
Gly B118
His B440
Phe B330
Phe A330
Glu B199
Tyr B334
Gly A441
3.9
Gly B441
Trp B84
Trp A84
Phe A331
Phe B331
Tyr A334
Trp A279
Trp B279
Tyr A70
Tyr B70

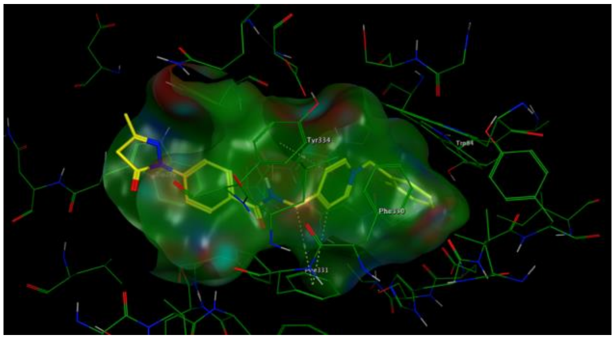

5f

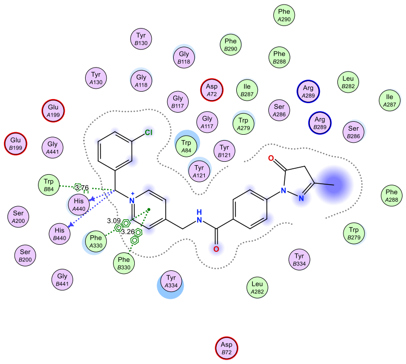
Phe A290
Tyr B130
Phe B290
Gly B118
Phe B288
Tyr A130
Gly A118
Asp A72
Ile B287
Arg A289
Leu B282
Ile A287
Gly B117
Gly A117
Trp A279
Ser A286
Arg B289
Glu A199
Ser B286
Glu B199
Gly A441
Trp A84
Tyr B121
Tyr A121
Phe A288
Trp B84
3.75
His A440
Ser A200
3.09
3.26
Trp B279
His B440
Phe A330
Tyr B334
Phe B330
Ser B200
Gly B441
Tyr A334
Leu A282
Asp B72

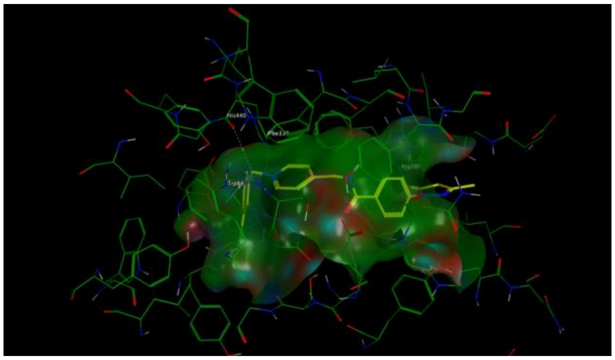

5g

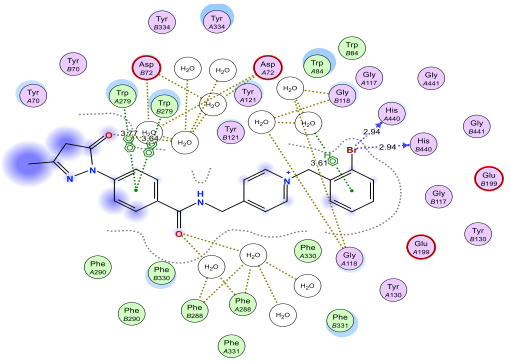
Tyr B334
Tyr A334
Trp B84
Tyr B70
Asp B72
Asp A72
Trp A84
Gly A117
Gly A441
Tyr A70
Trp A279
Trp B279
Tyr A121
Gly B118
His A440
Gly B441
Tyr B121
2.94
His B440
3.77
3.64
3.61
Glu B199
Gly B117
Tyr B130
Phe A330
Glu A199
Gly A118
Phe A290
Phe B330
Phe A288
Tyr A130
Phe B290
Phe B288
Phe B331
Phe A331

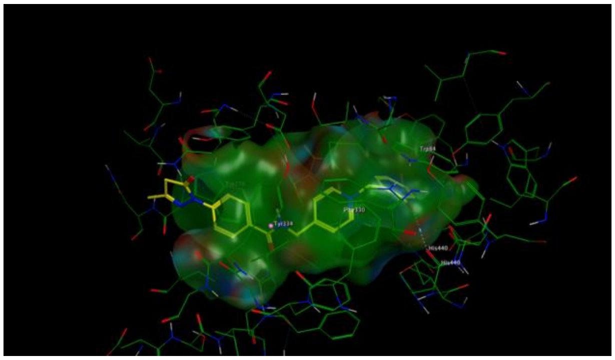

5h

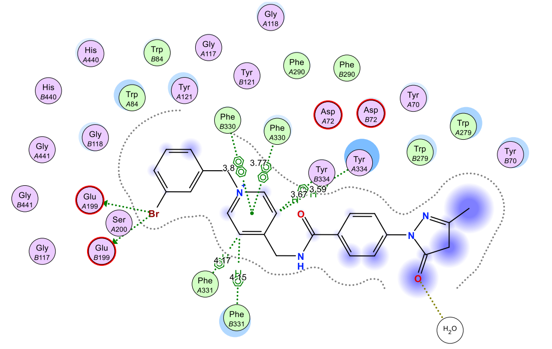
Gly A118
His A440
Trp B84
Gly A117
Tyr B121
Phe A290
Phe B290
His B440
Trp A84
Tyr A121
Asp A72
Asp B72
Tyr A70
Trp A279
Gly A441
Gly B118
Phe B330
Phe A330
Trp B279
Tyr B70
Tyr B334
Tyr A334
Gly B441
Glu A199
Ser A200
Gly B117
Glu B199
Phe A331
Phe B331
Br
H2O

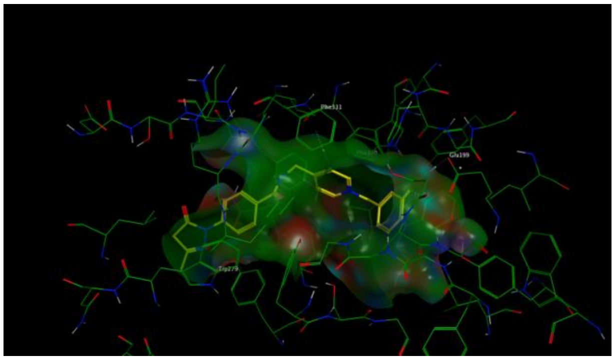

5i

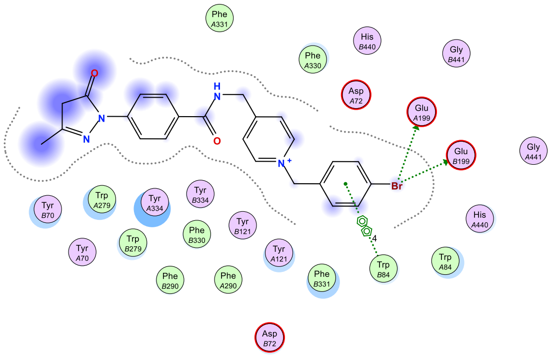
Phe A331
His B440
Phe A330
Gly B441
Asp A72
Glu A199
Glu B199
Gly A441
Br
Tyr B70
Trp A279
Tyr A334
Tyr B334
Phe B330
Tyr B121
His A440
Tyr A70
Trp B279
Tyr A121
Trp A84
Phe B290
Phe A290
Phe B331
Trp B84
Asp B72

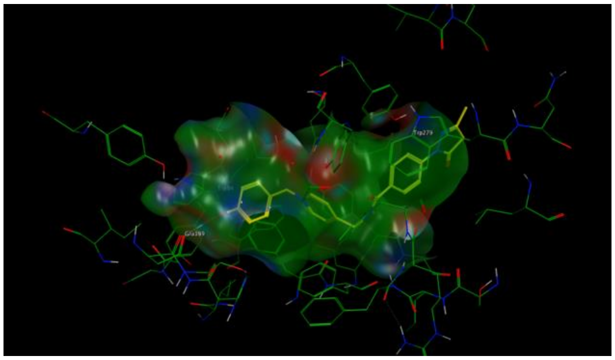

5j

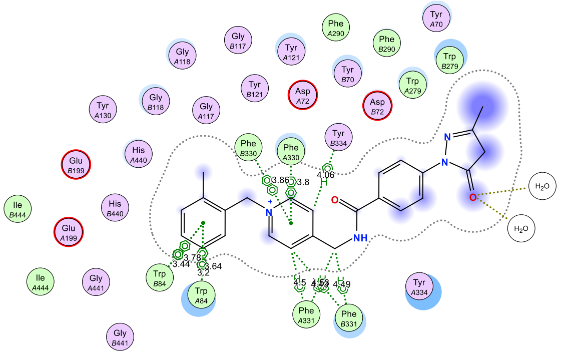
Phe A290
Tyr A70
Gly B117
Phe B290
Gly A118
Tyr A121
Trp B279
Tyr B70
Tyr B121
Trp A279
Tyr A130
Gly B118
Gly A117
Asp A72
Asp B72
Phe B330
Phe A330
Tyr B334
Glu B199
His A440
His B440
Ile B444
Glu A199
Trp B84
Trp A84
Ile A444
Gly A441
Phe A331
Phe B331
Tyr A334
Gly B441
H2O
H2O

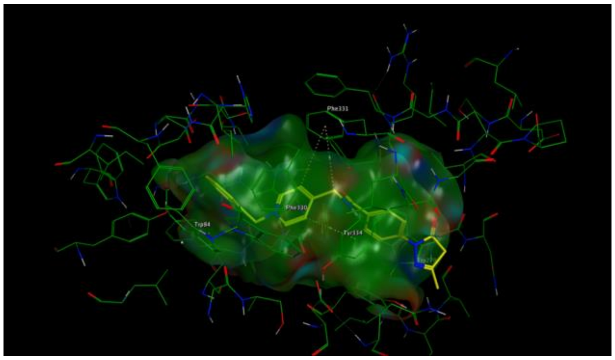

5k

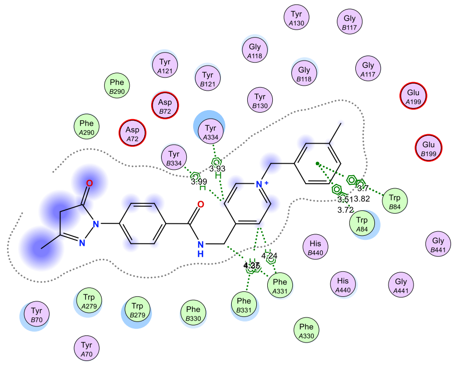
Tyr A130
Gly B117
Gly A118
Tyr A121
Tyr B121
Gly B118
Gly A117
Phe B290
Asp B72
Tyr B130
Glu A199
Phe A290
Asp A72
Tyr A334
Tyr B334
Glu B199
Trp B84
Trp A84
His B440
Gly B441
His A440
Gly A441
Phe A331
Phe B331
Tyr B70
Trp A279
Trp B279
Phe B330
Phe A330
Tyr A70

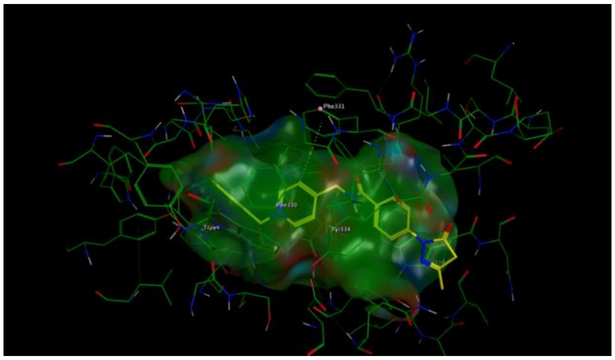

5l

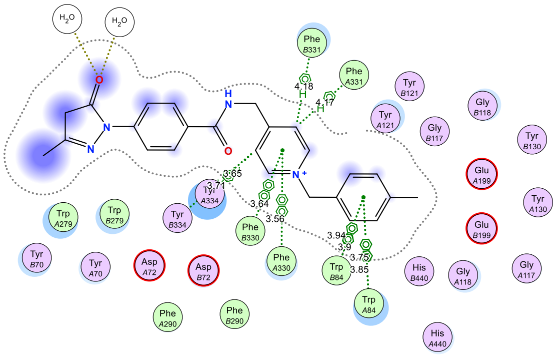
H2O
H2O
O
N
N
H
N
O
N+
Phe B331
Phe A331
Tyr B121
Tyr A121
Gly B117
Gly B118
Tyr B130
Glu A199
Glu B199
Tyr A130
Gly A117
Gly A118
His B440
His A440
Trp A84
Trp B84
Phe A330
Phe B330
Tyr A334
Tyr B334
Trp A279
Trp B279
Tyr B70
Tyr A70
Asp A72
Asp B72
Phe A290
Phe B290
4.18
4.17
3.65
3.71
3.64
3.56
3.94
3.9
3.75
3.85

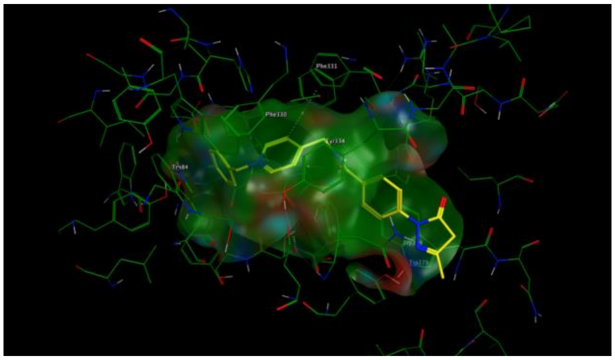

AChE inhibition dose response curves

3

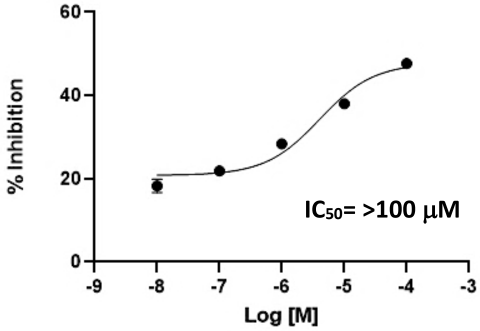

5a

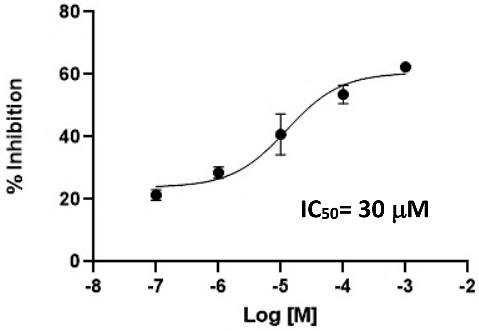

5b

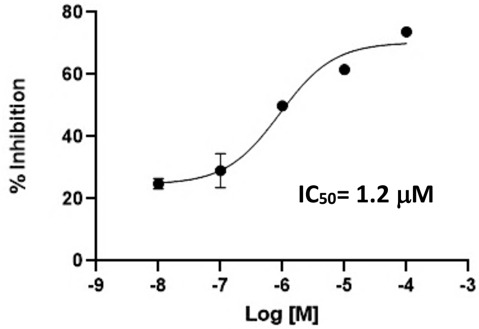

5c

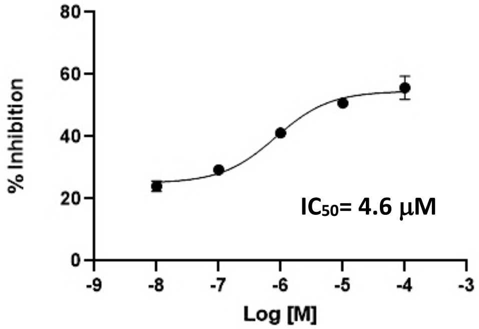

5d

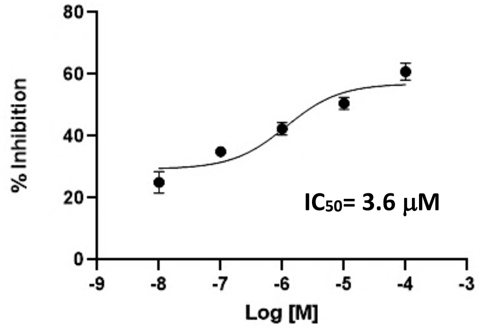

5e

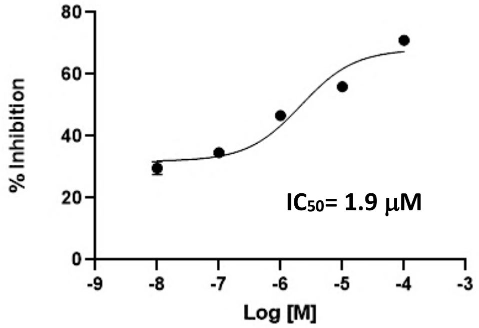

5f

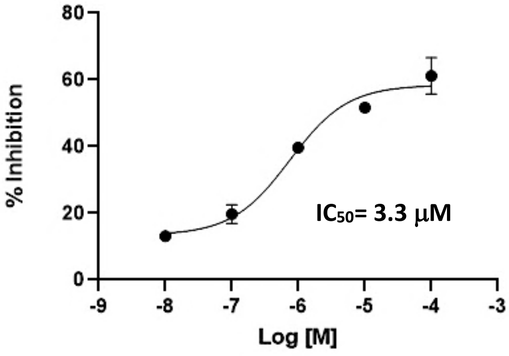

5g

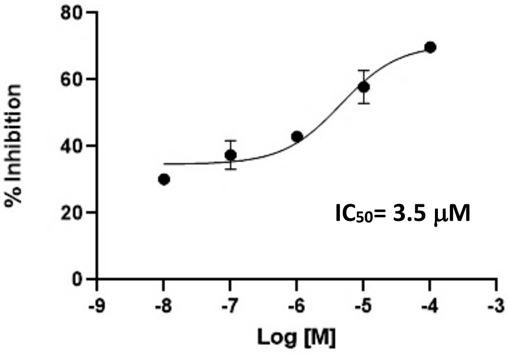

5h

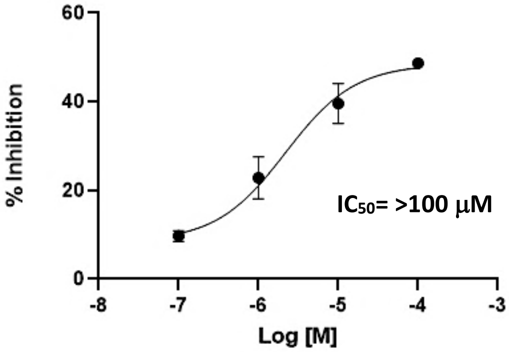
% Inhibition
Log [M]
IC$_{50}$= >100 μM

5i

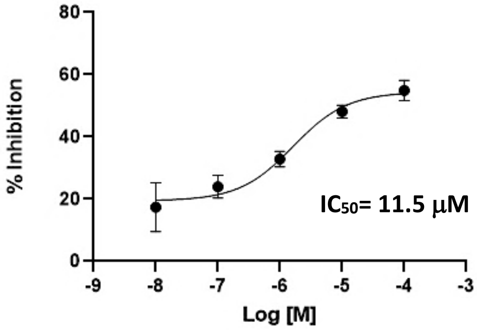
% Inhibition
Log [M]
IC$_{50}$= 11.5 μM

5j

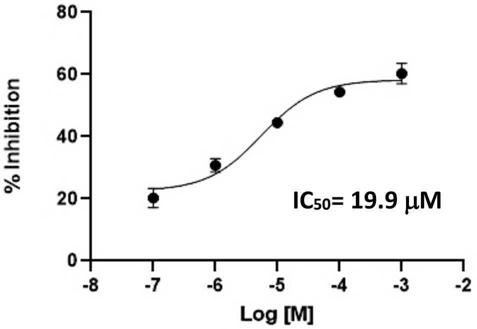
% Inhibition
Log [M]
IC$_{50}$= 19.9 μM

5k

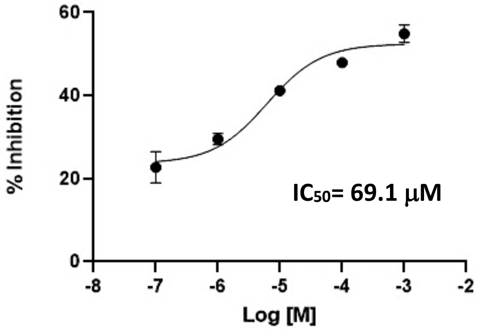
% Inhibition
Log [M]
IC$_{50}$= 69.1 μM

5l

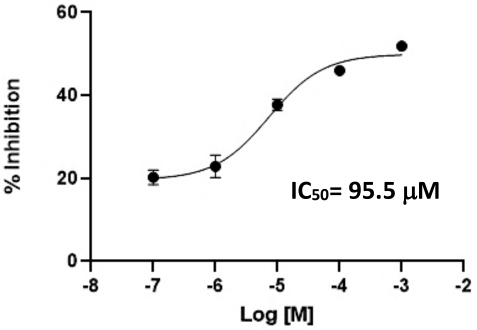
% Inhibition
Log [M]
IC$_{50}$= 95.5 μM

BuChE inhibition dose response curves

3

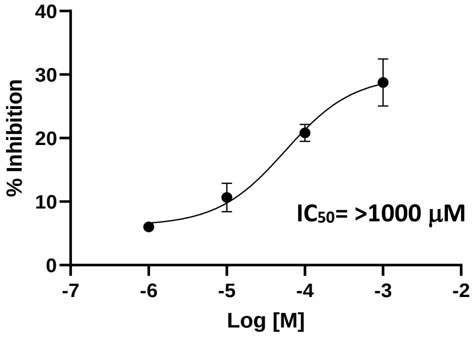

5a

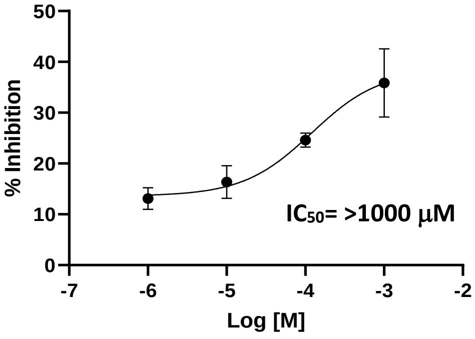

5b

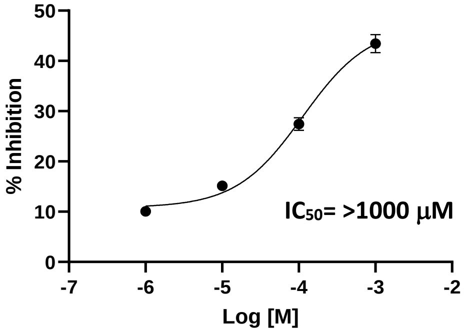

5c

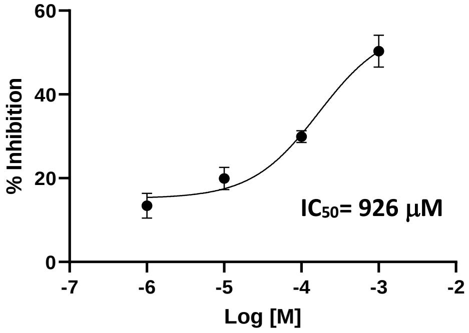

5d

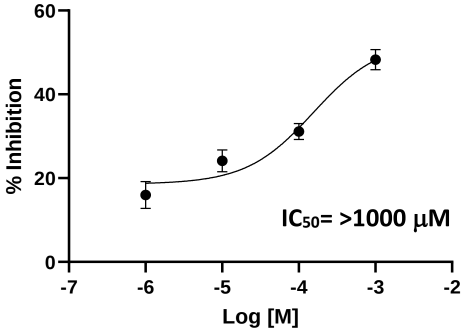

5e

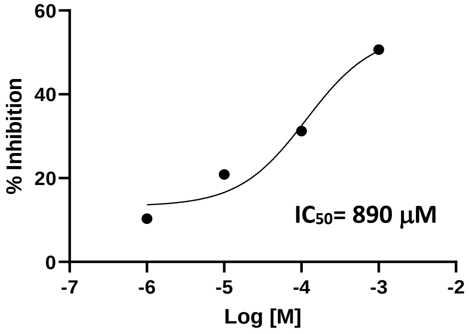

5f

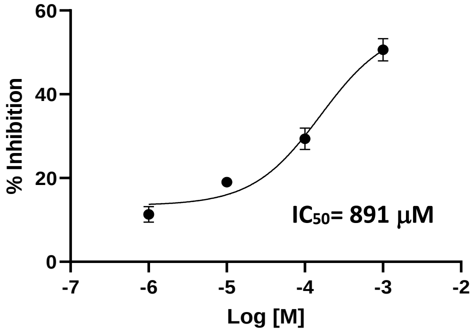

5g

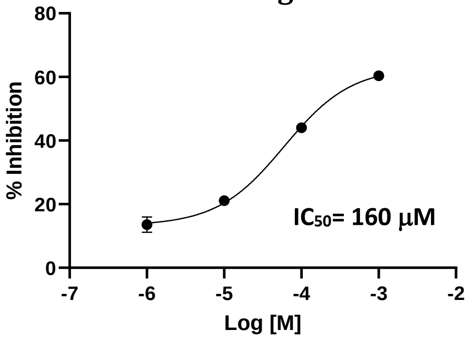

5h

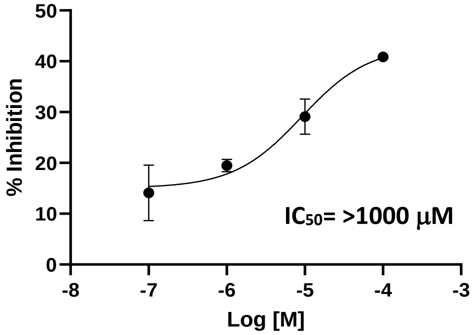
% Inhibition
Log [M]
IC_{50}= >1000 μM

5i

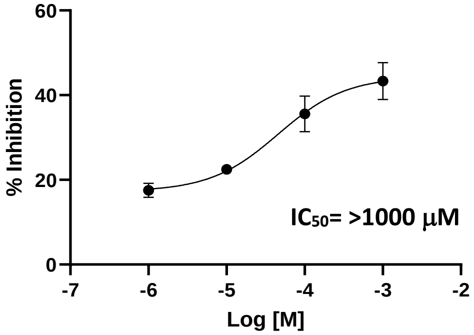
% Inhibition
Log [M]
IC_{50}= >1000 μM

5j

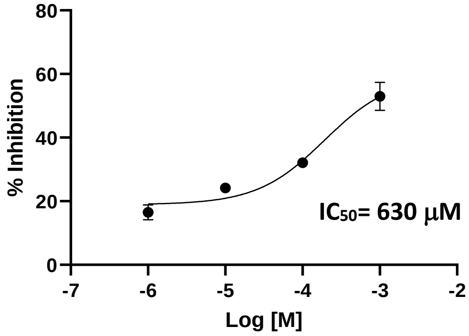
% Inhibition
Log [M]
IC_{50}= 630 μM

5k

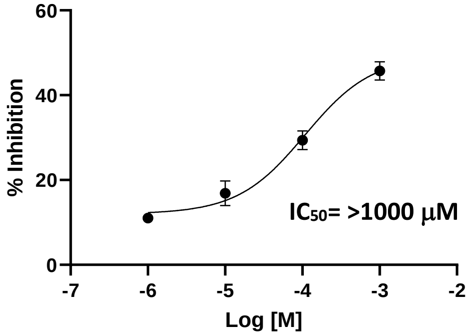
% Inhibition
Log [M]
IC_{50}= >1000 μM

5l

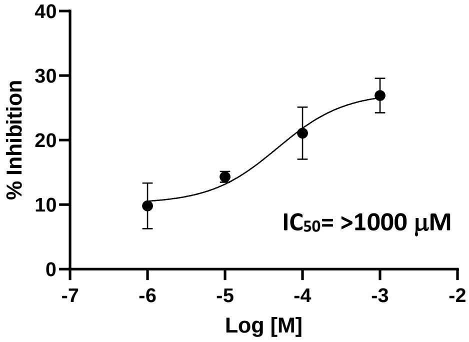
% Inhibition
Log [M]
IC_{50}= >1000 μM

Antioxidant activity (DPPH^{+}) dose response curves

1

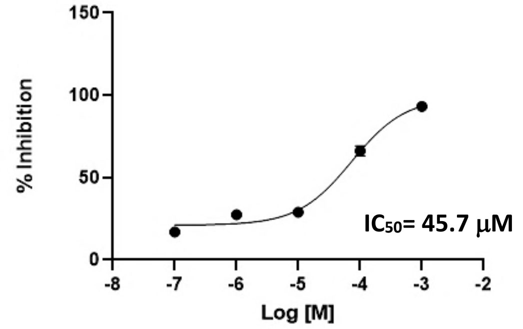

3

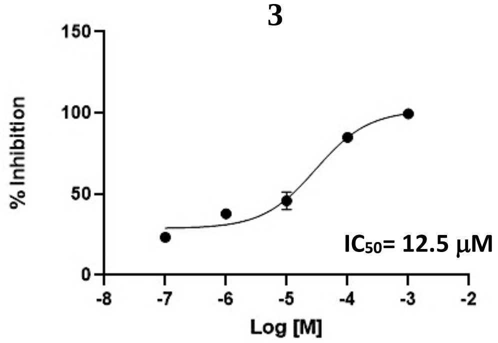

5a

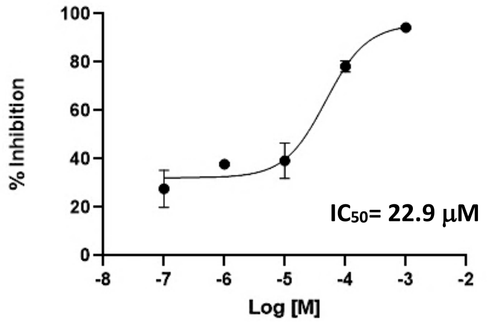

5b

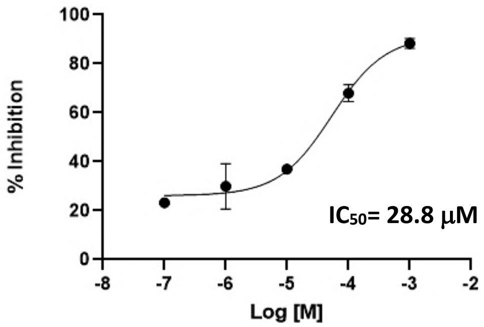

5c

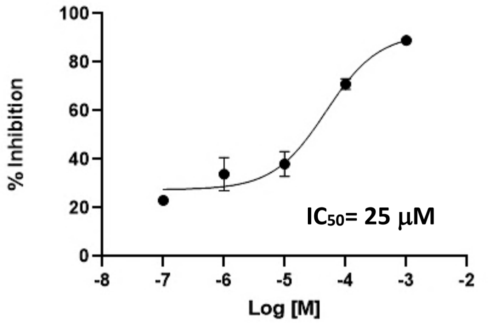

5d

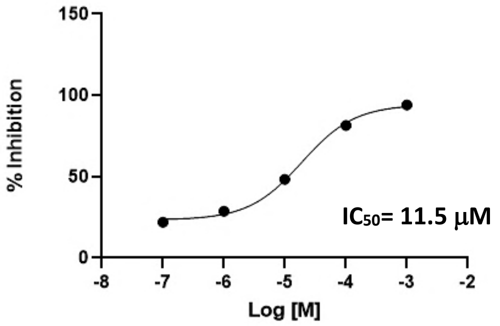

5e

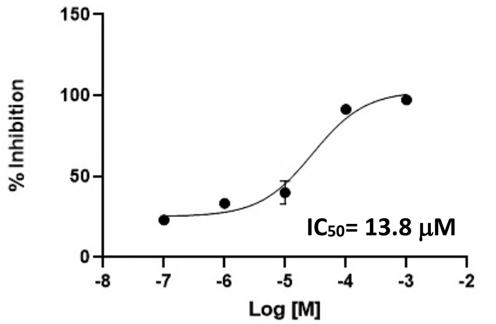

5f

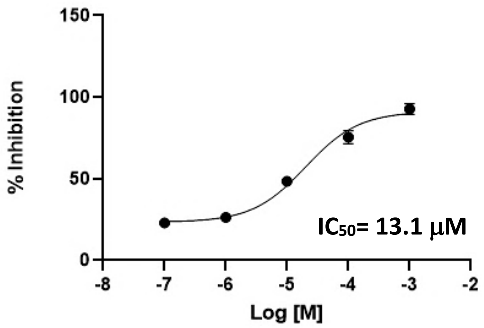

5g

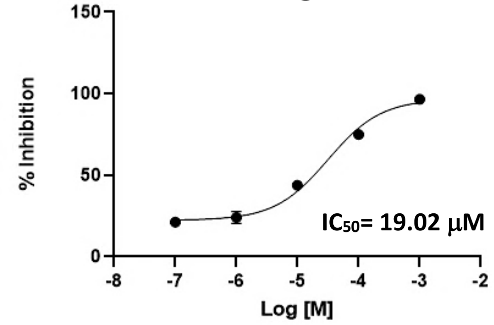

5h

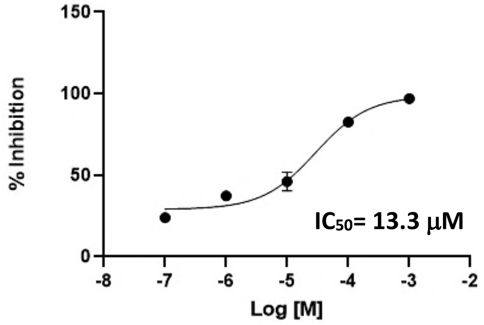

5i

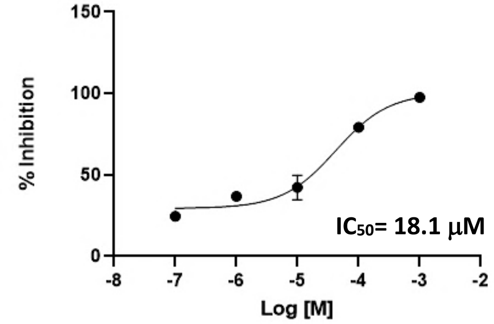

5j

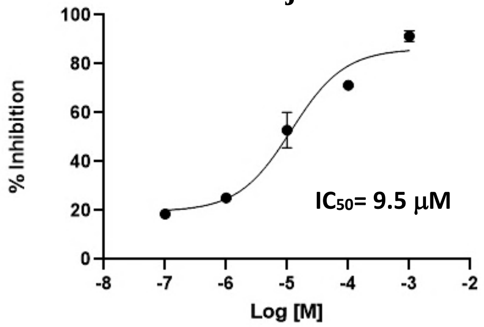

5k

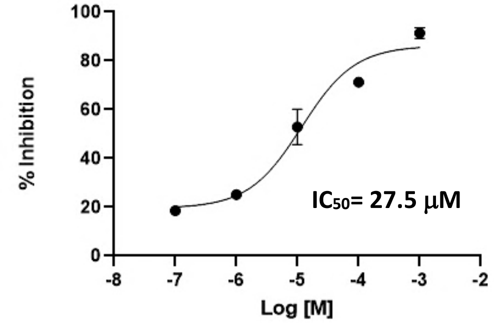

5l

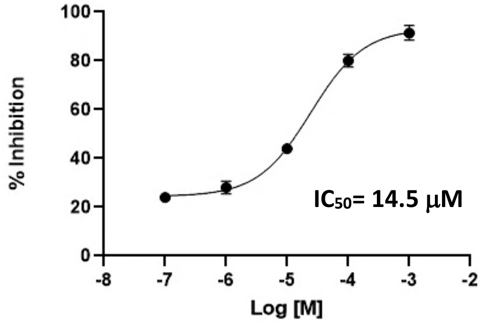

Trolox (Control)

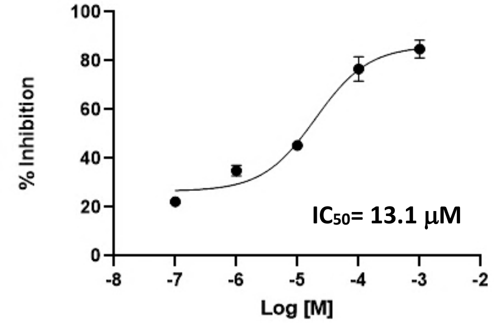

Spectral Data

^{1}H NMR; ^{13}C NMR; HSQC NMR; IR; MS

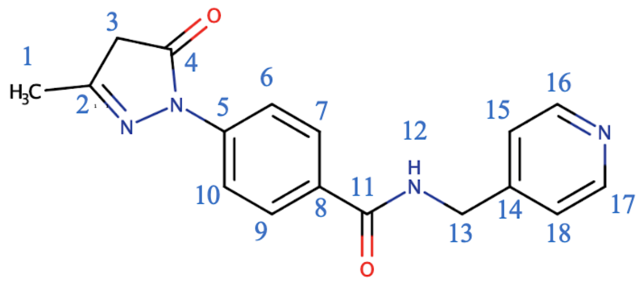

Compound **3**

Spectrum 1: ^{1}H NMR Compound 3

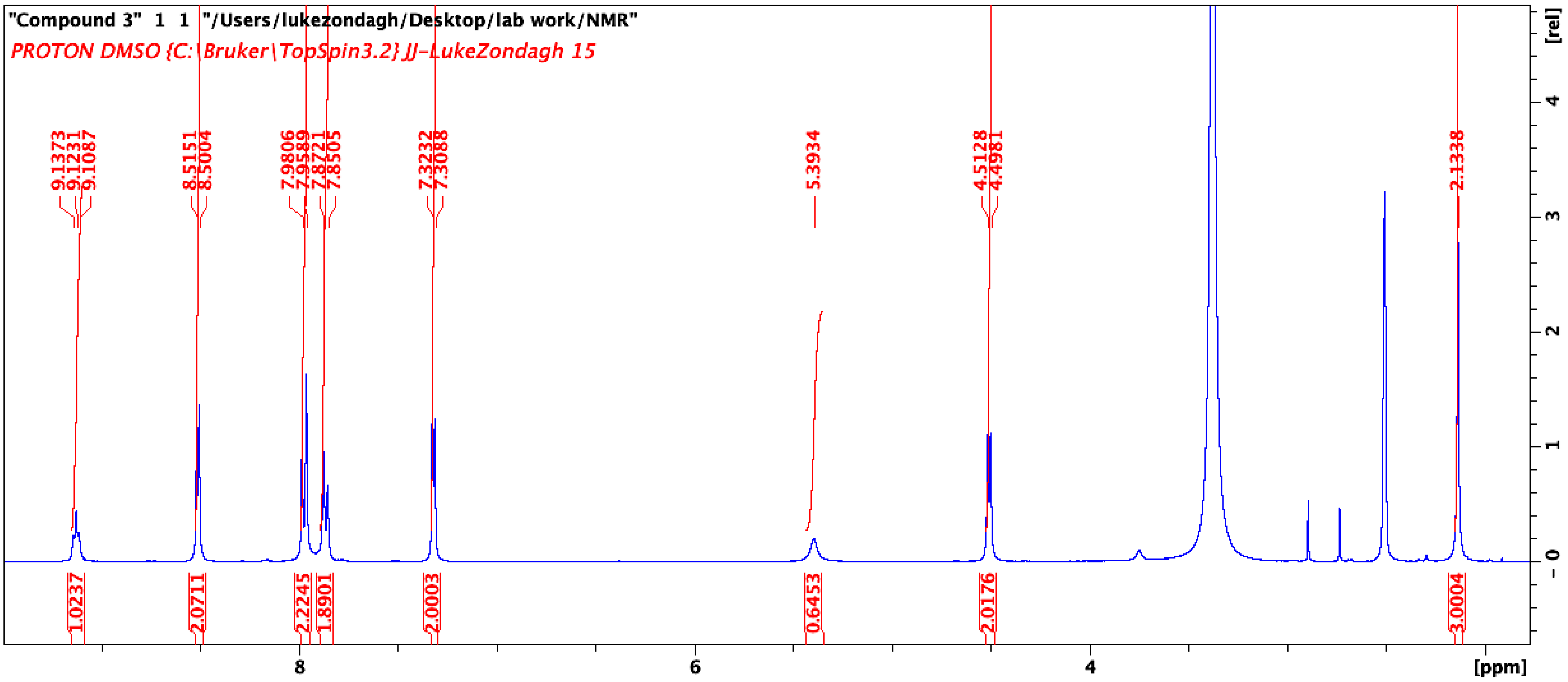

Spectrum 2: ^{13}C NMR Compound 3

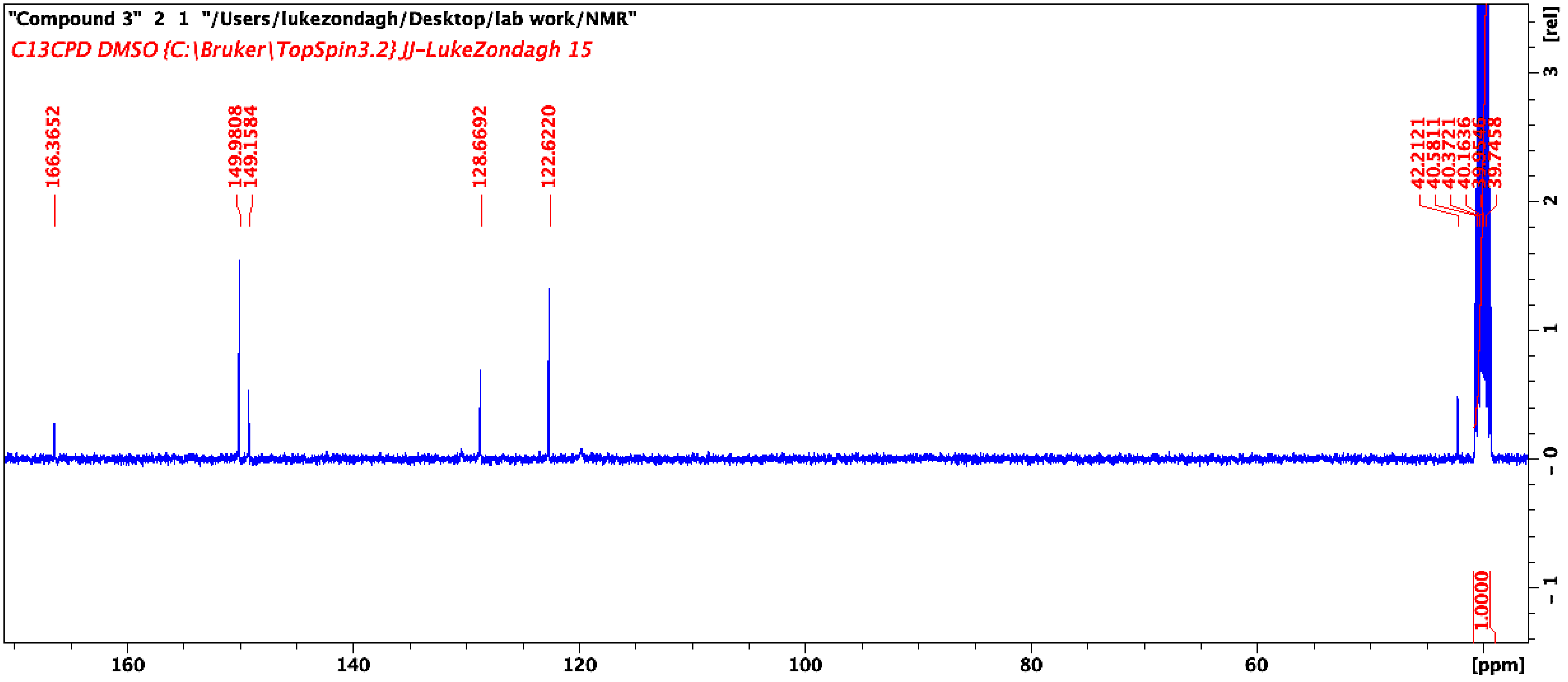

Spectrum 3: HSQC NMR Compound 3

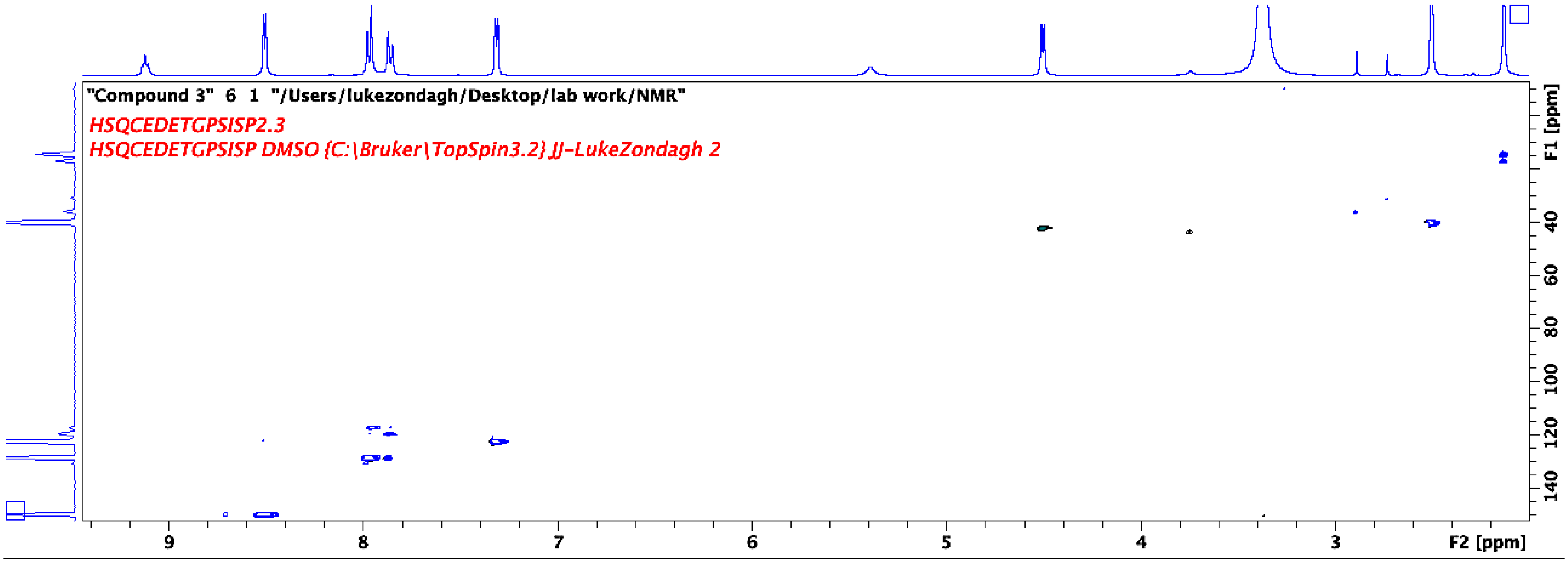

Spectrum 4: IR Compound 3

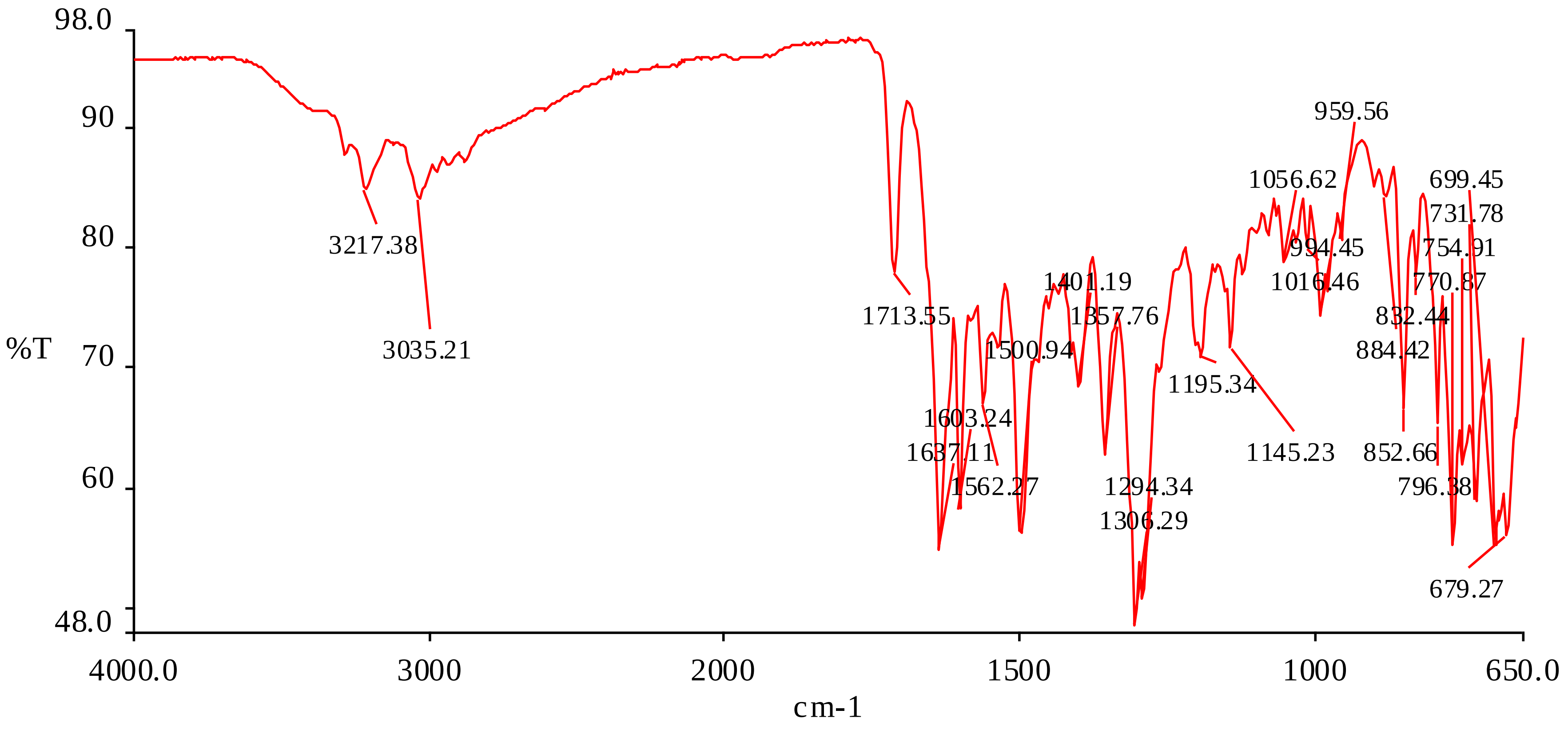

Spectrum 5: MS Compound 3

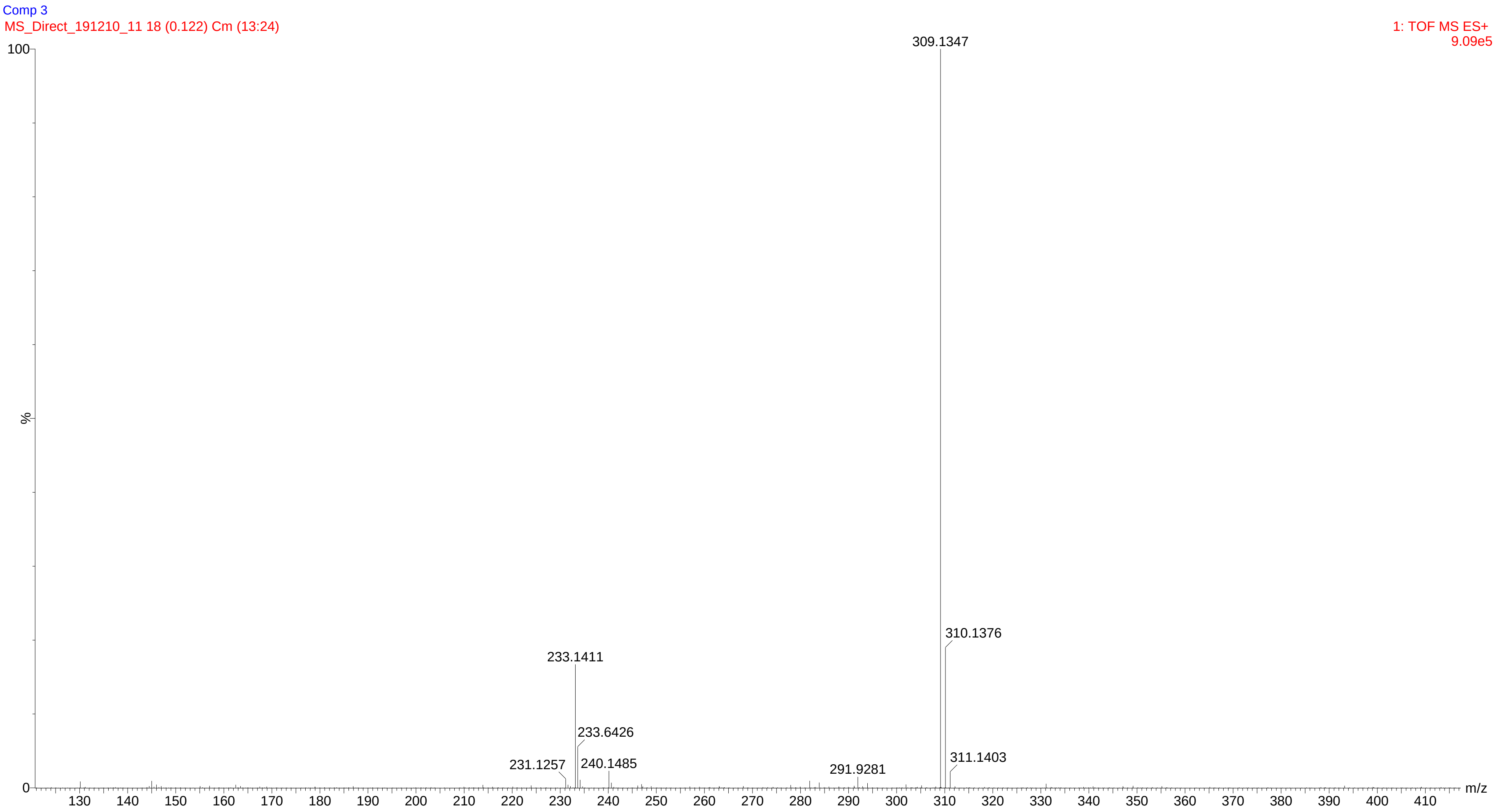

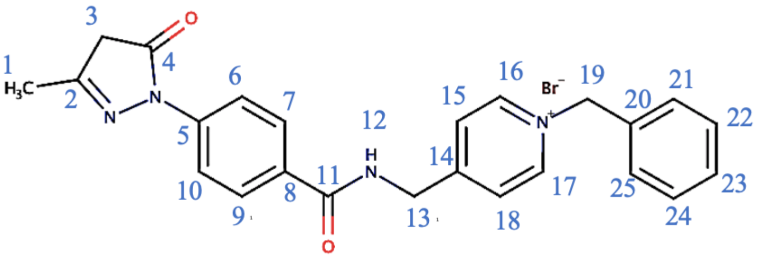

Compound **5a**

Spectrum 6: ^{1}H NMR Compound 5a

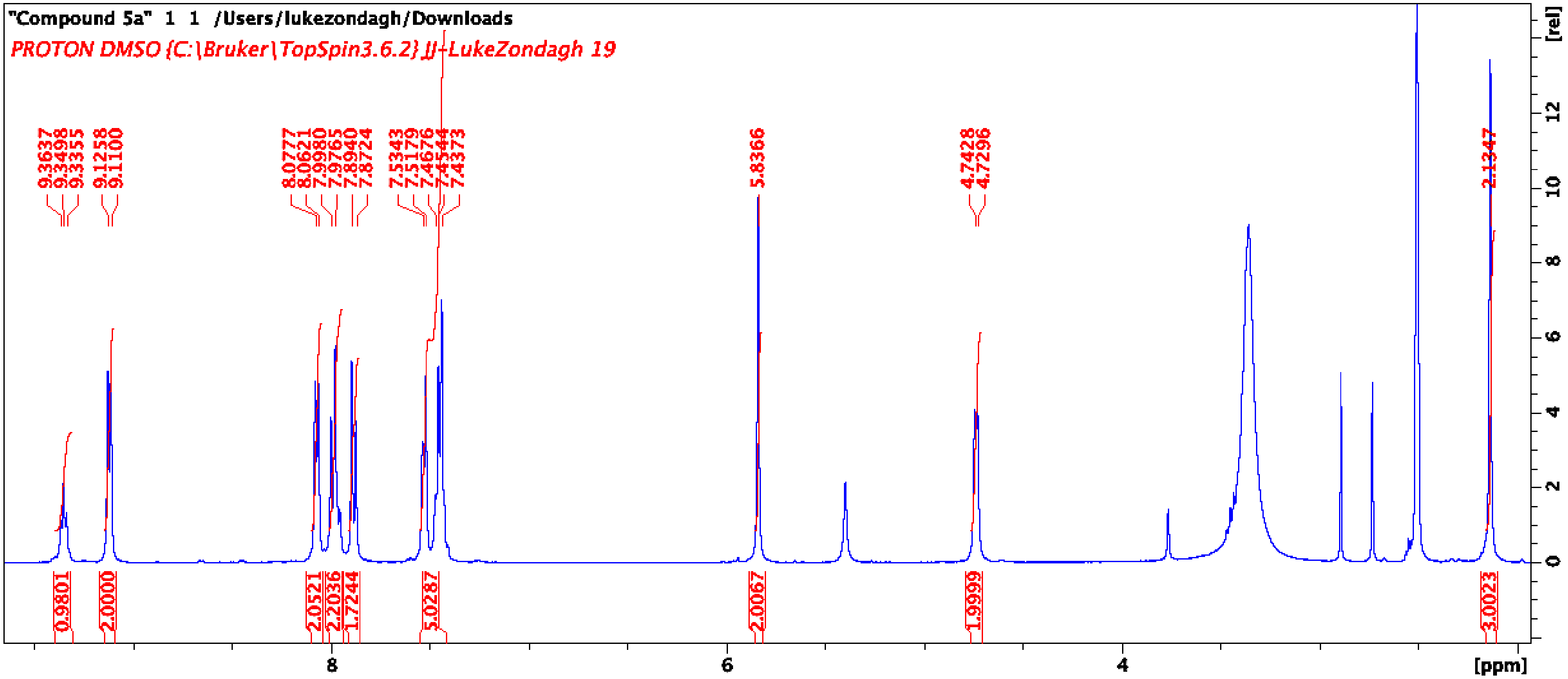

Spectrum 7: ^{13}C NMR Compound 5a

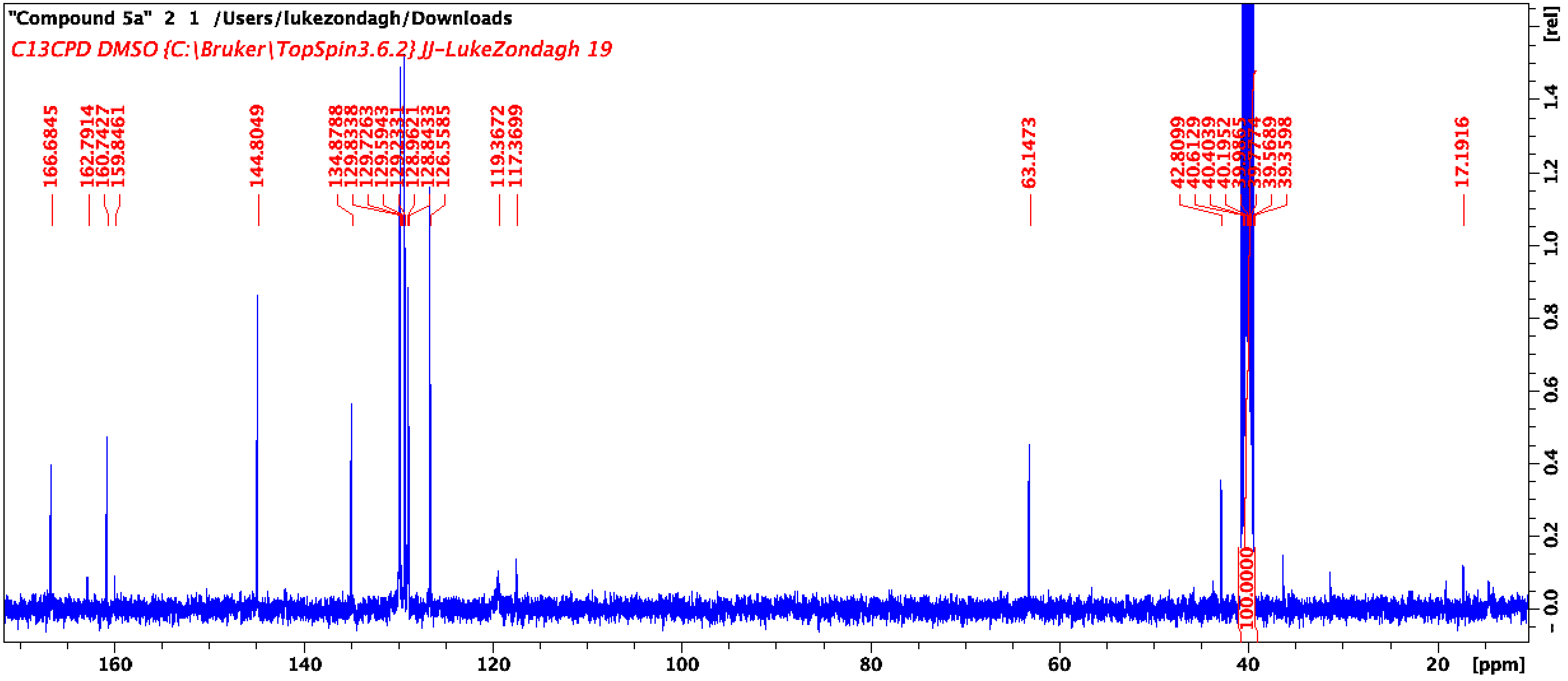

Spectrum 8: IR Compound 5a

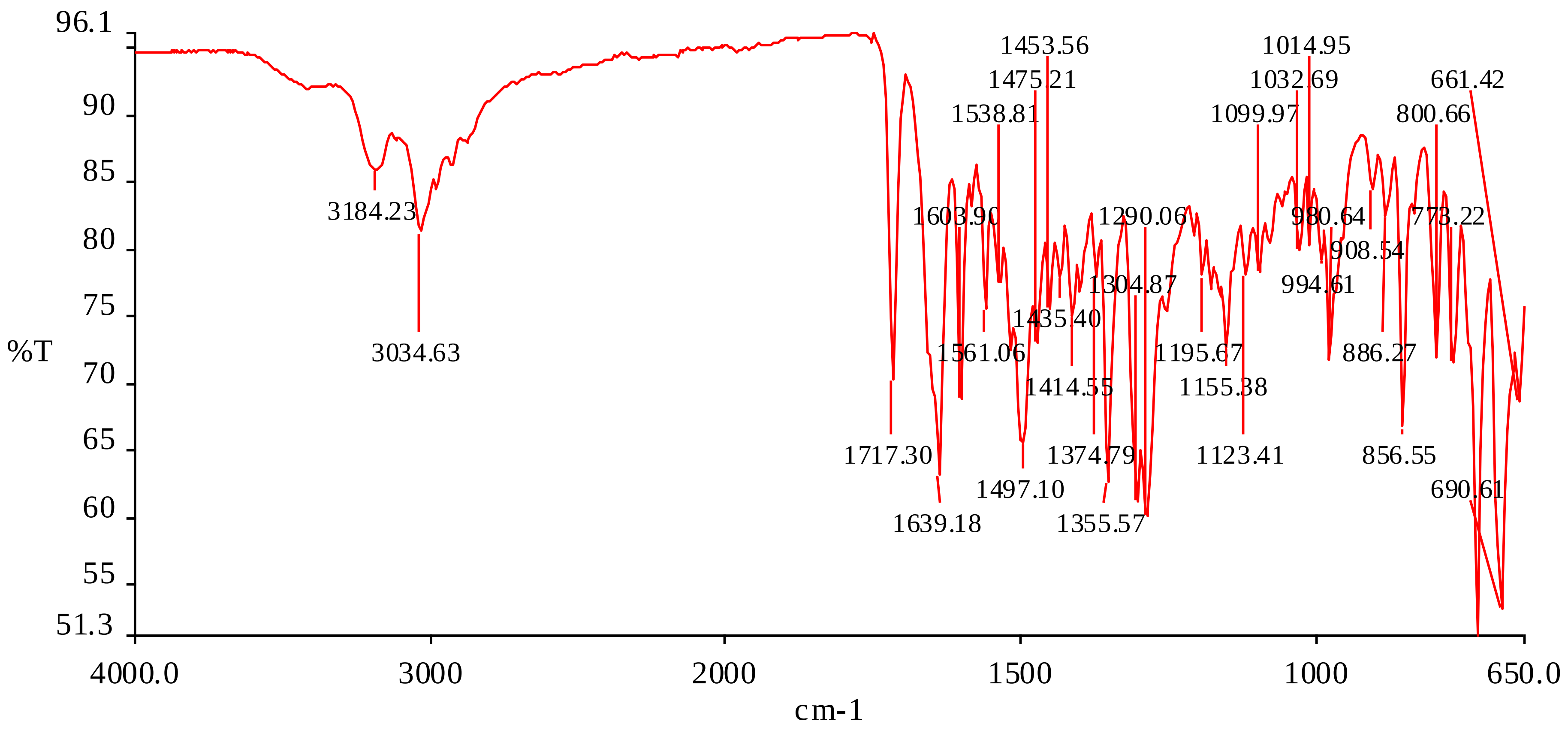

Spectrum 9: MS Compound 5a

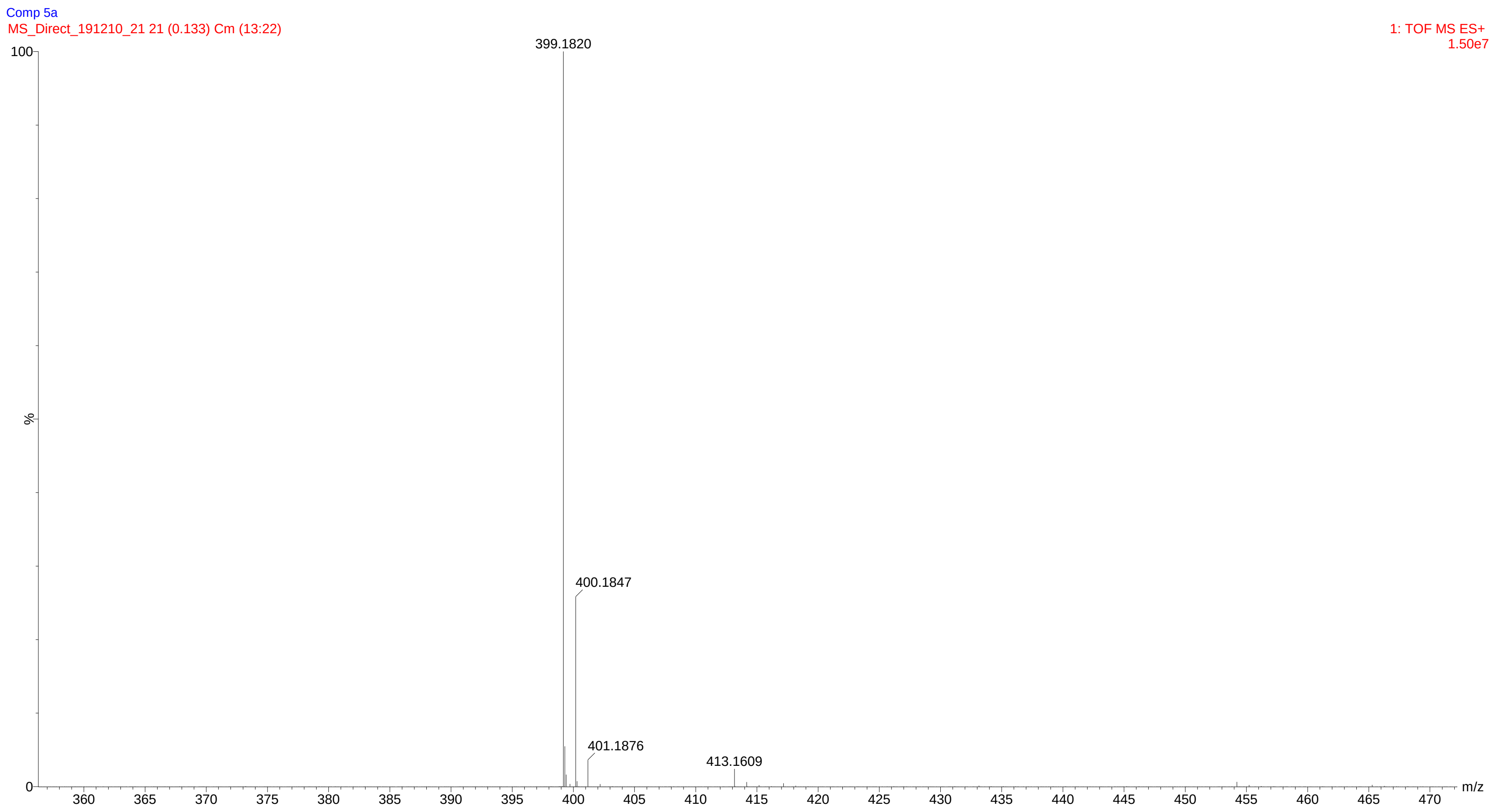

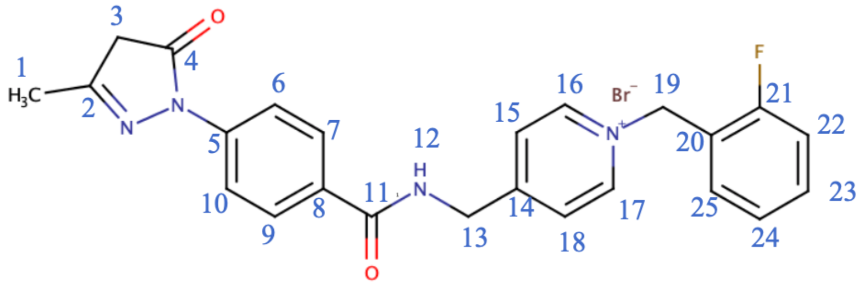

Compound **5b**

Spectrum 10: ^{1}H NMR Compound 5b

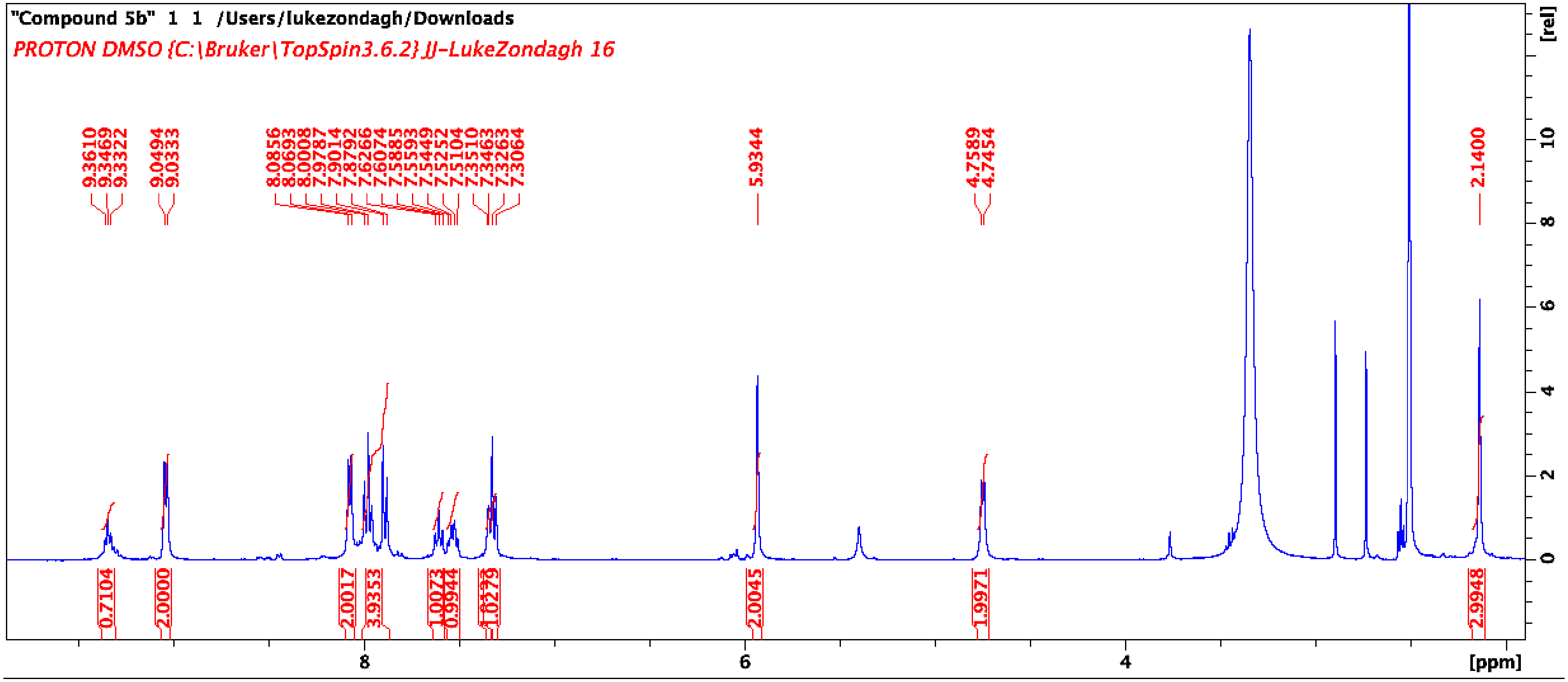

Spectrum 11: ^{13}C NMR Compound 5b

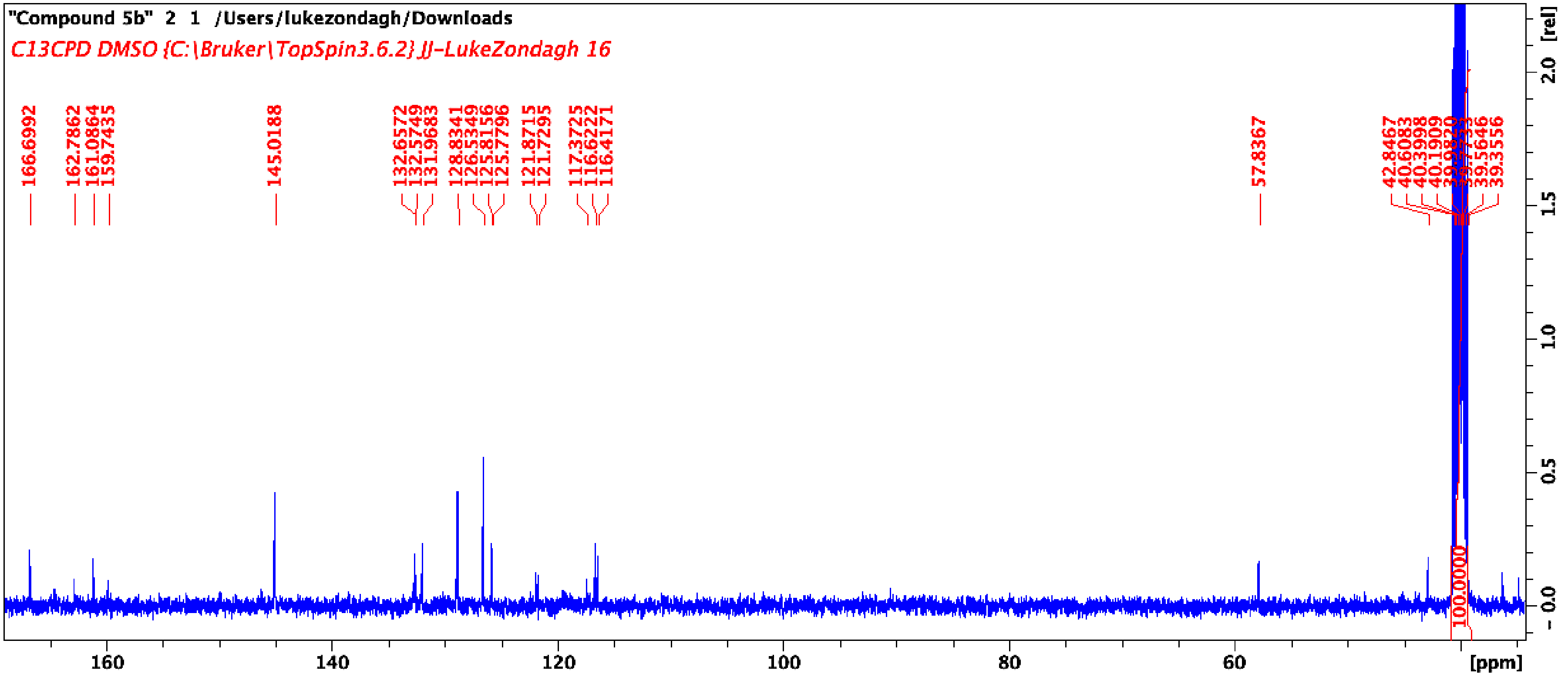

Spectrum 12: IR Compound 5b

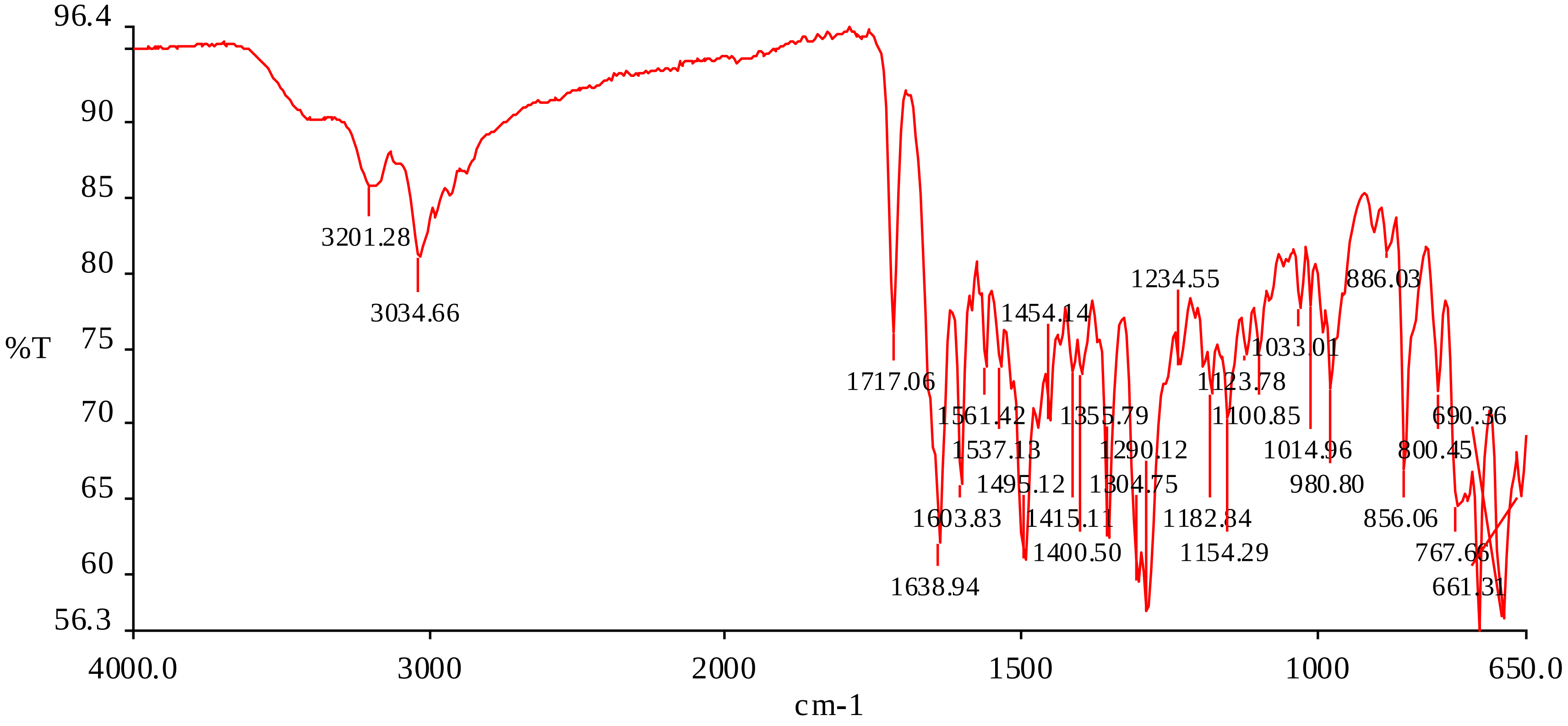

<u>Spectrum 13: MS Compound 5b</u>

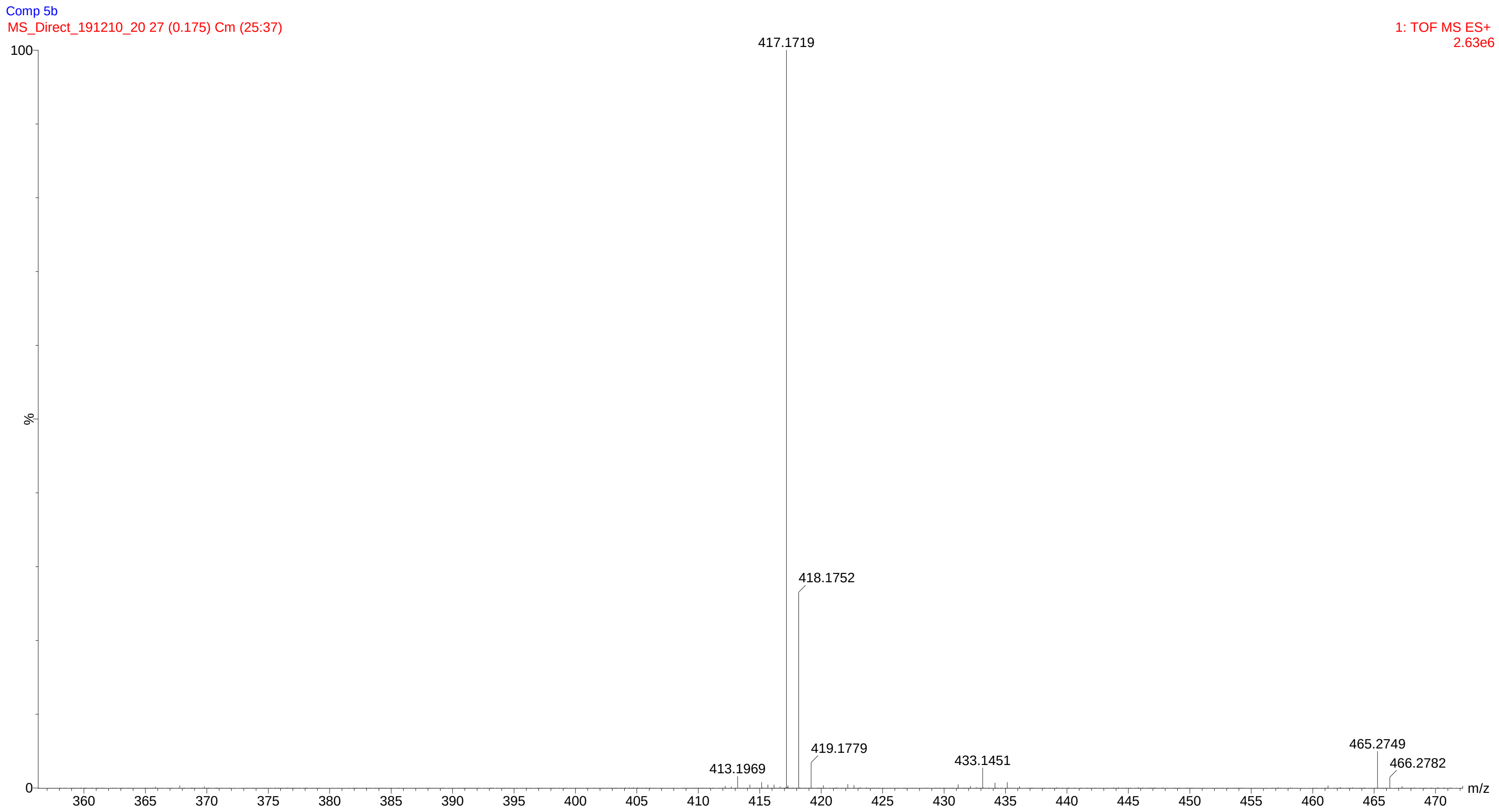

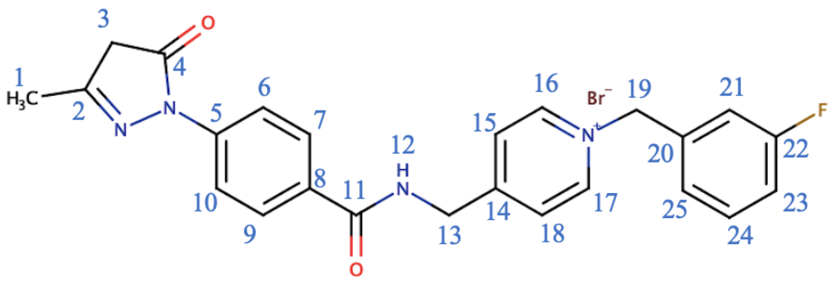

Compound **5c**

Spectrum 14: ^{1}H NMR Compound 5c

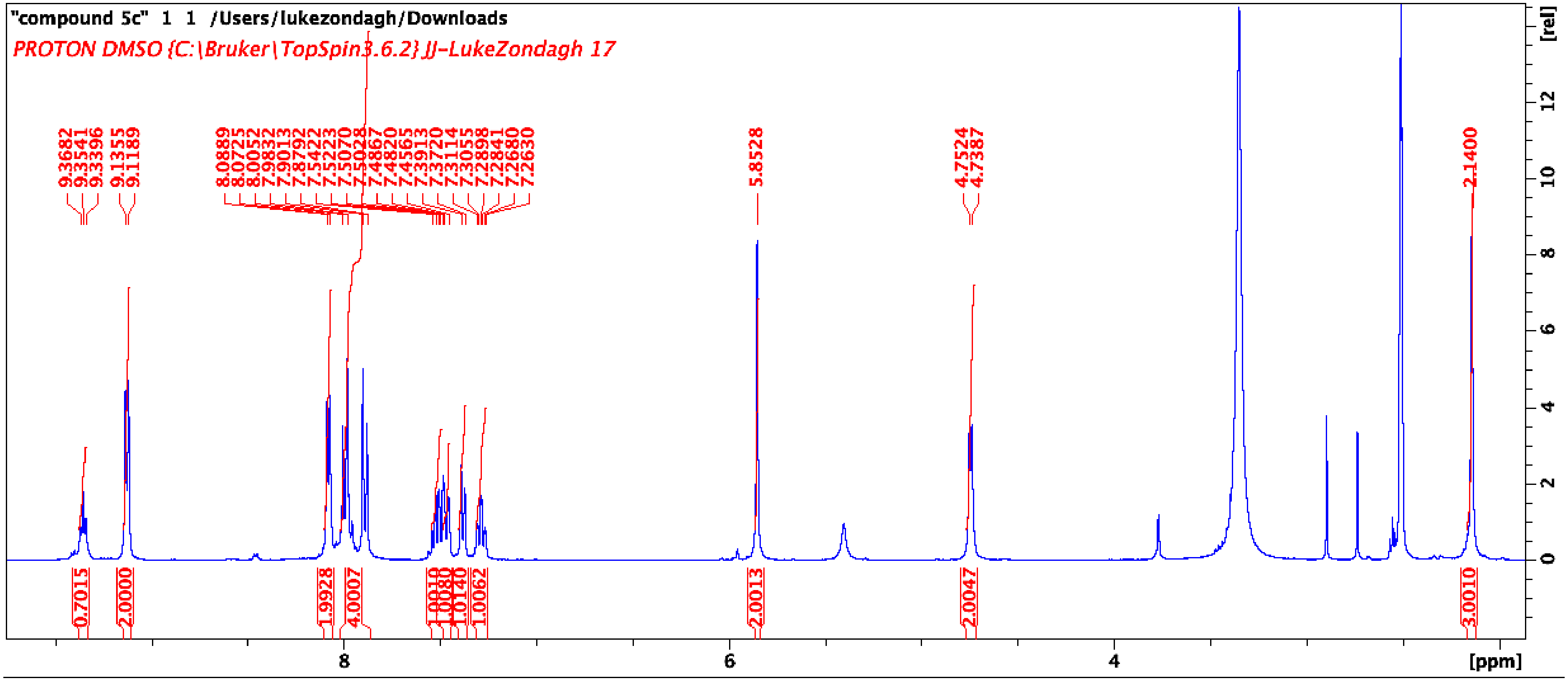

Spectrum 15: ^{13}C NMR Compound 5c

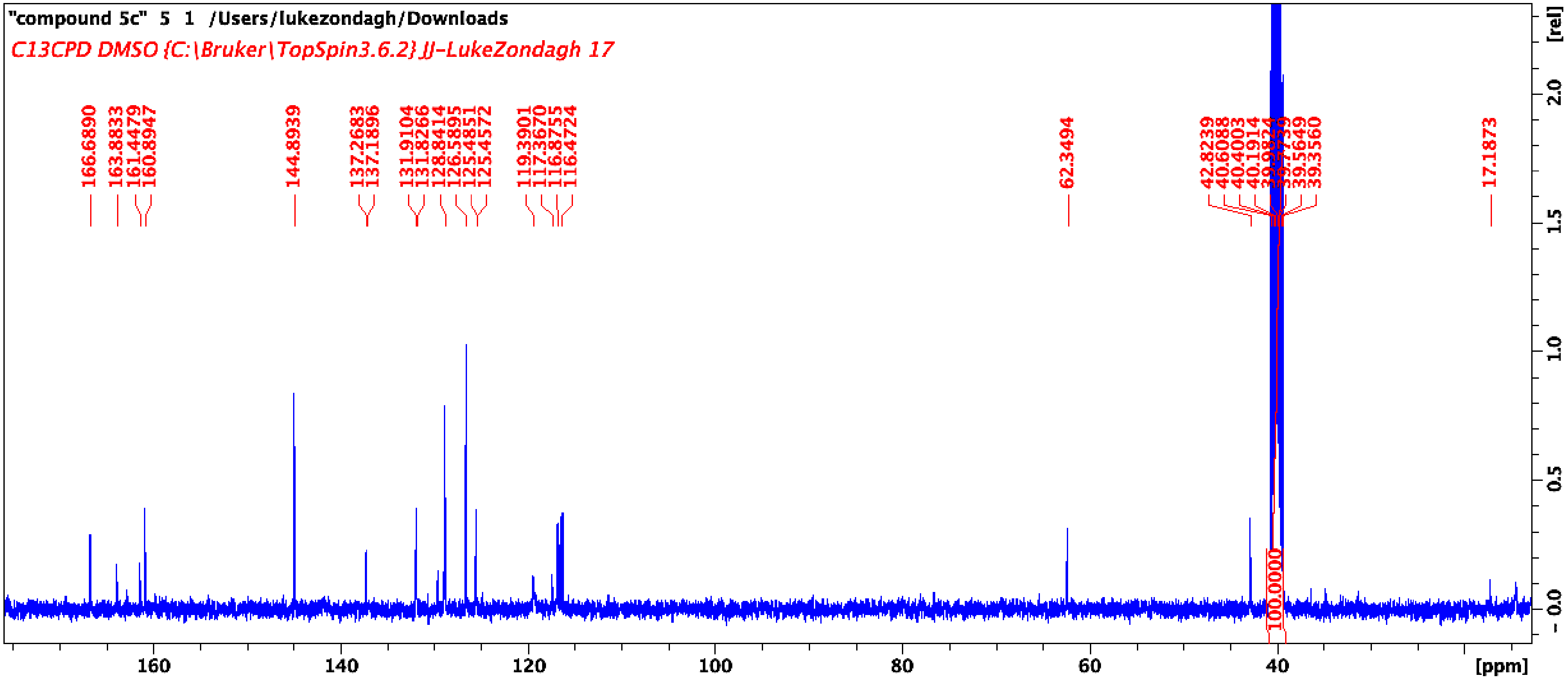

Spectrum 16: IR Compound 5c

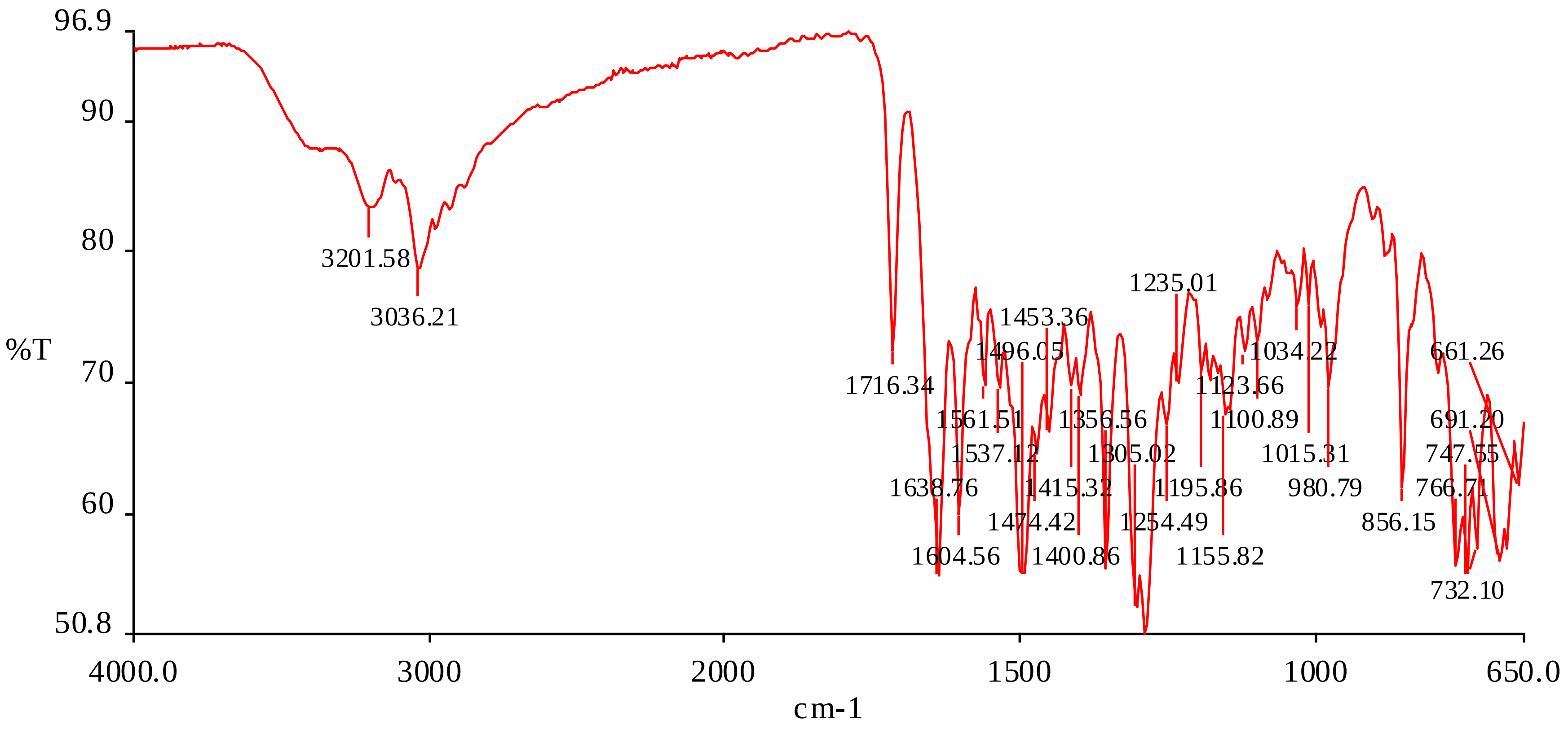

Spectrum 17: MS Compound 5c

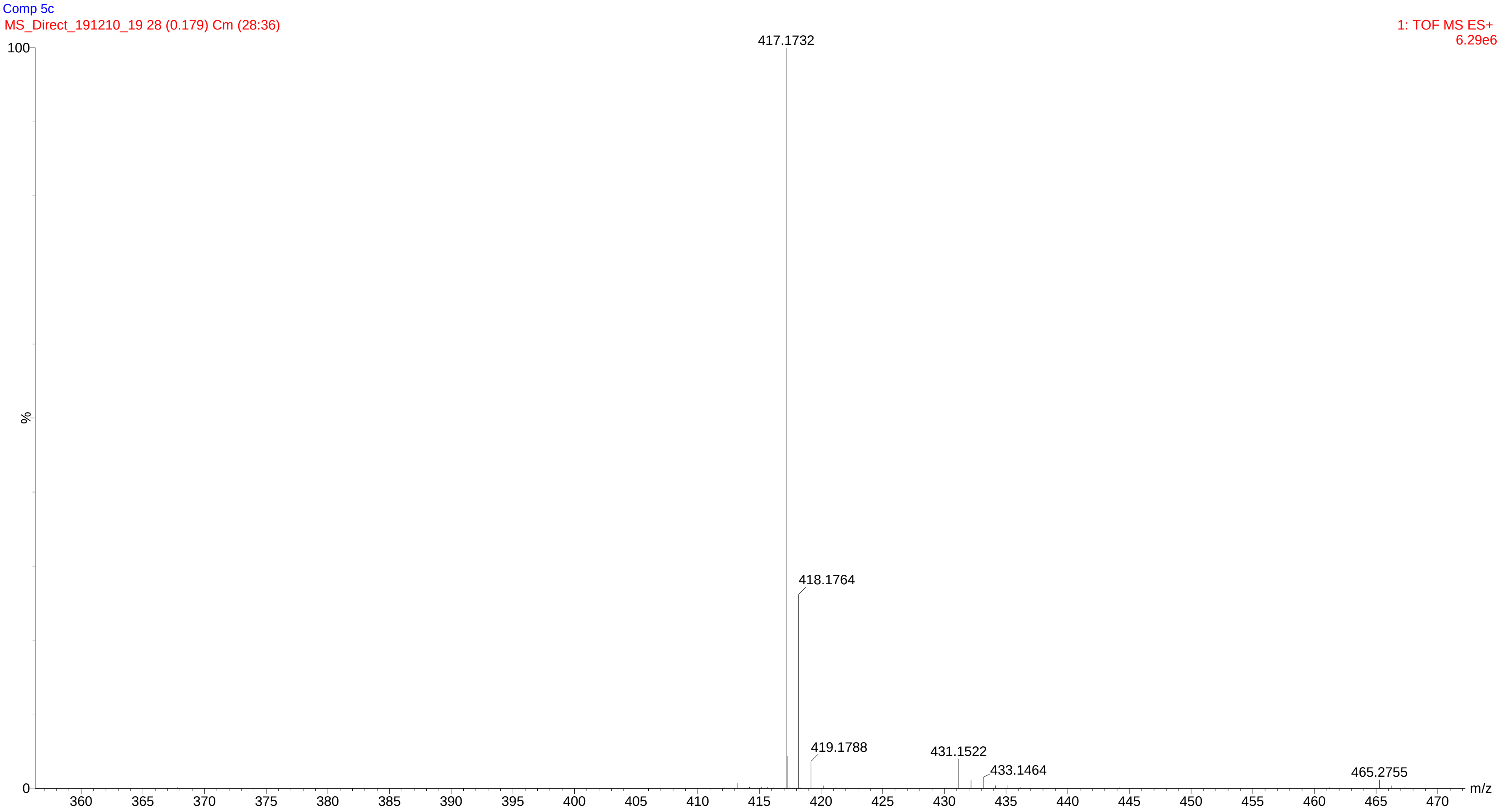

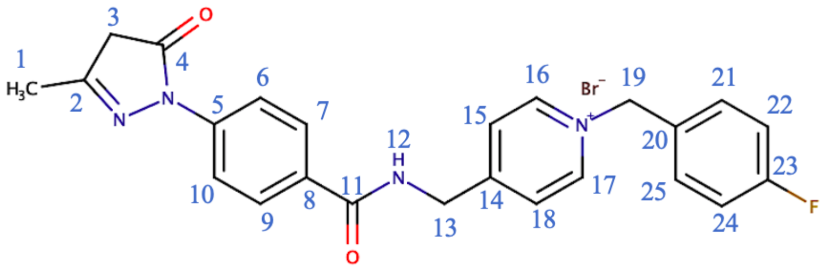

Compound **5d**

Spectrum 18: ^{1}H NMR Compound 5d

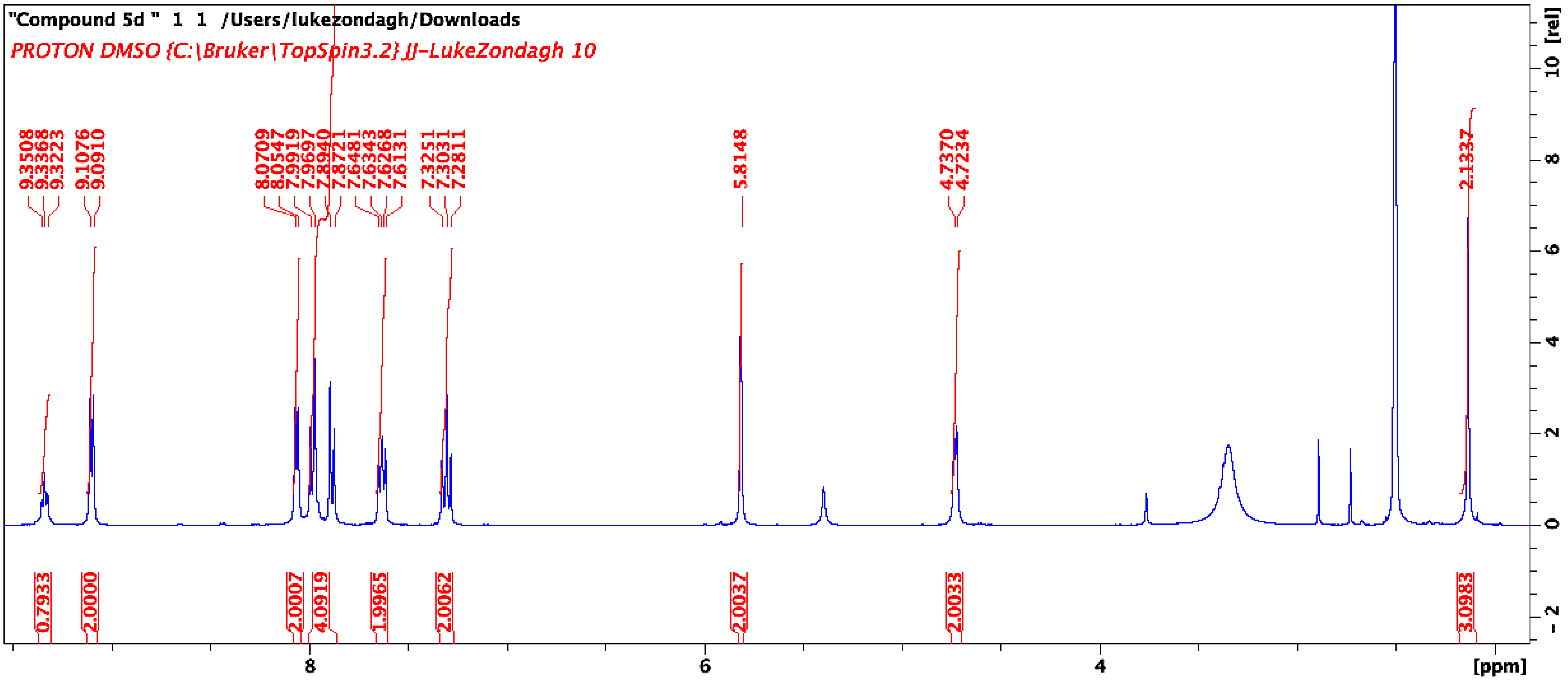

Spectrum 19: ^{13}C NMR Compound 5d

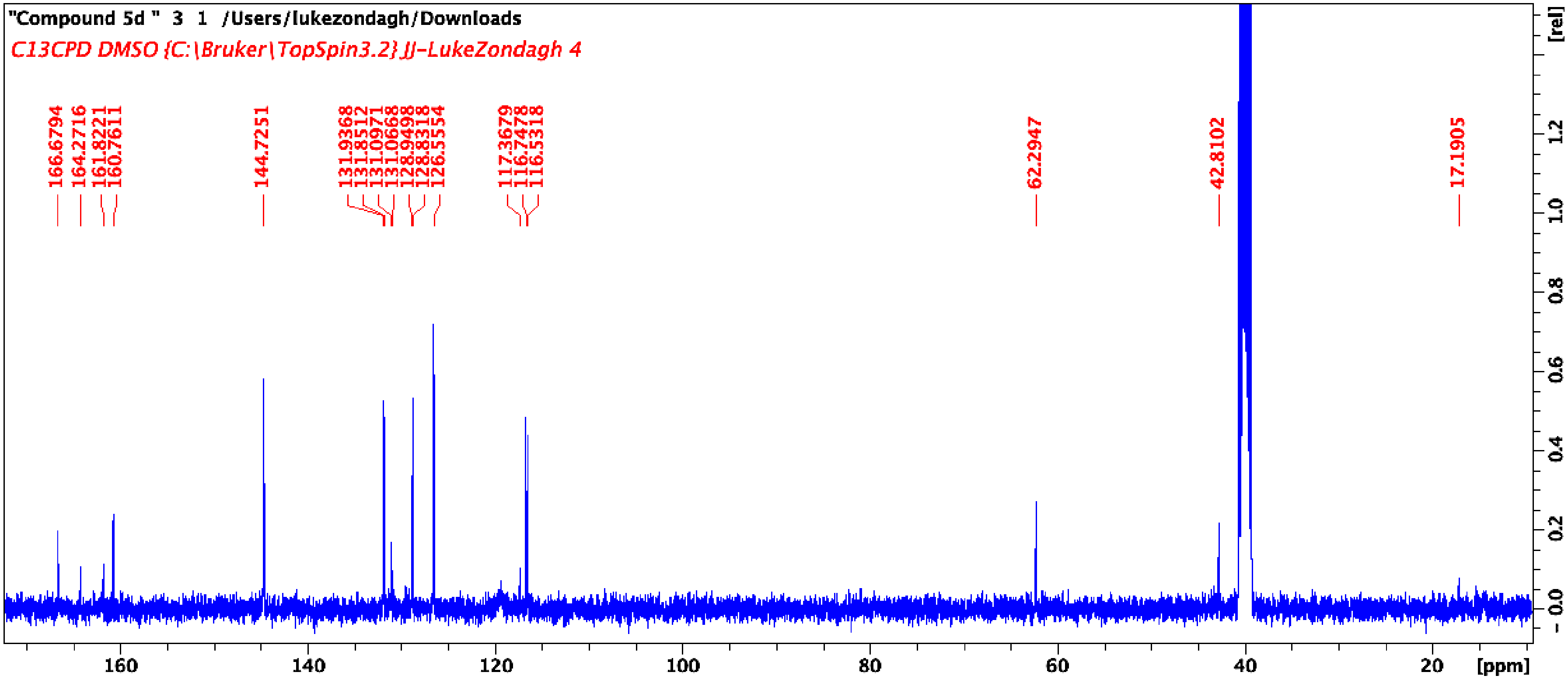

Spectrum 20: IR Compound 5d

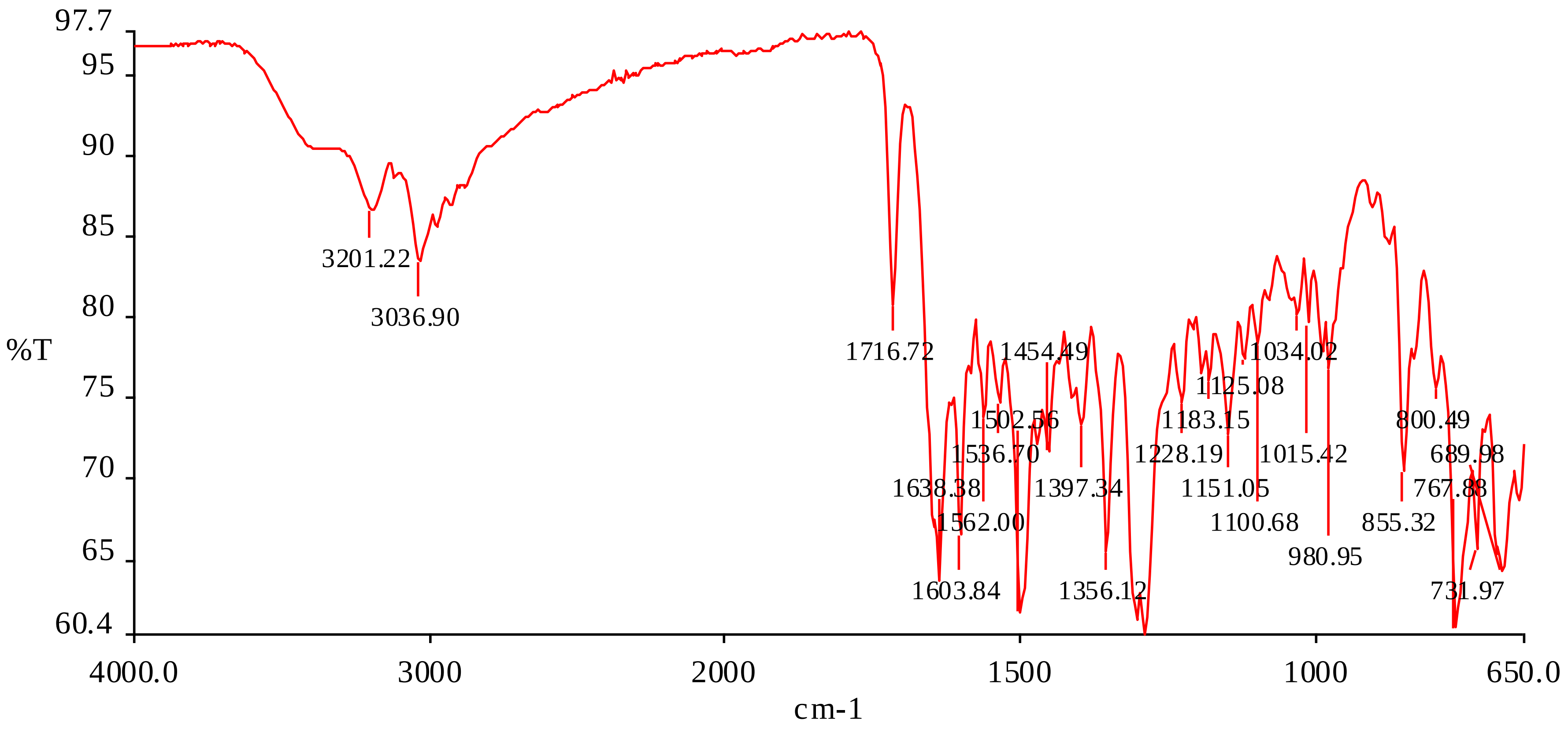

Spectrum 21: MS Compound 5d

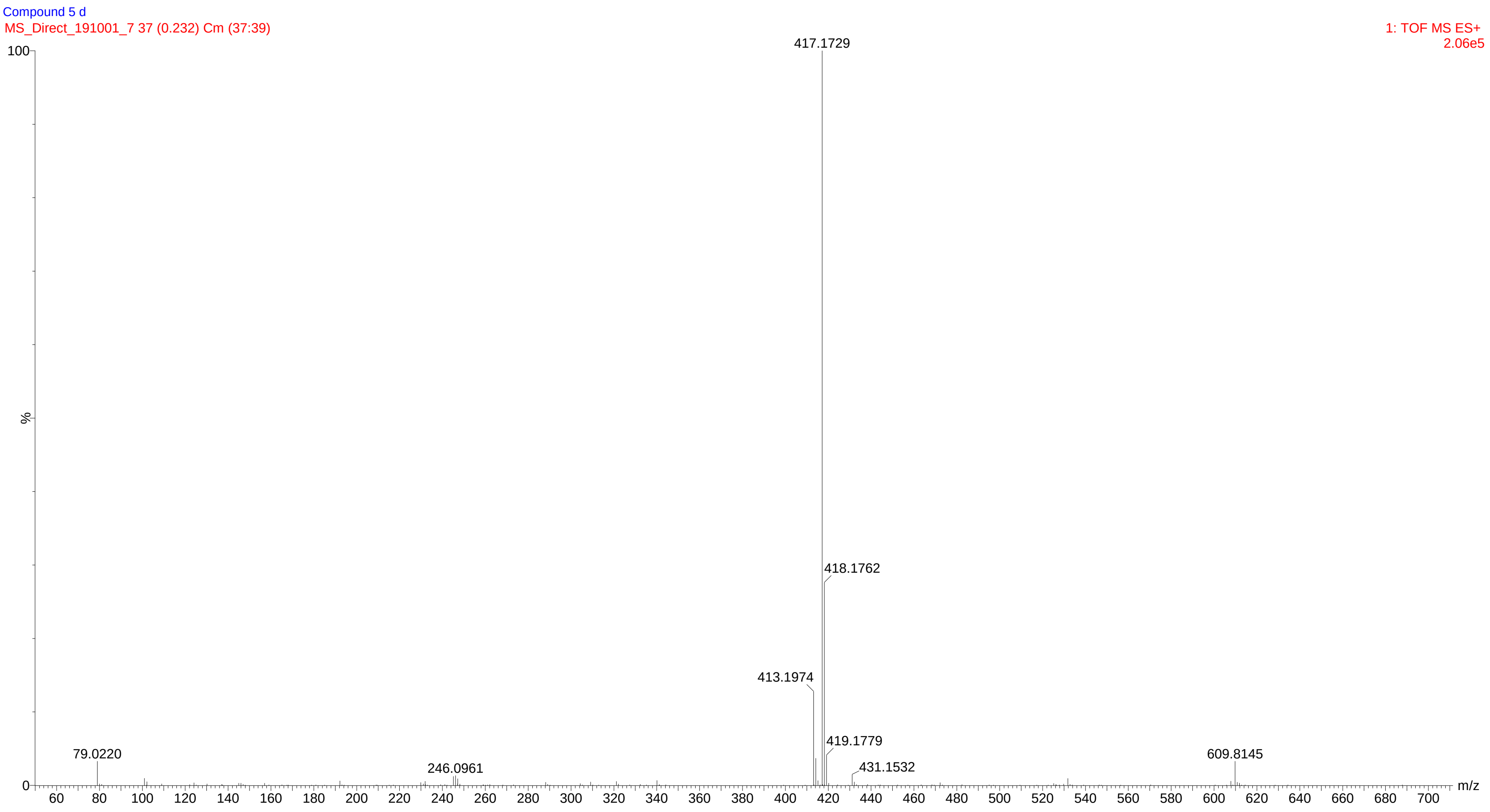

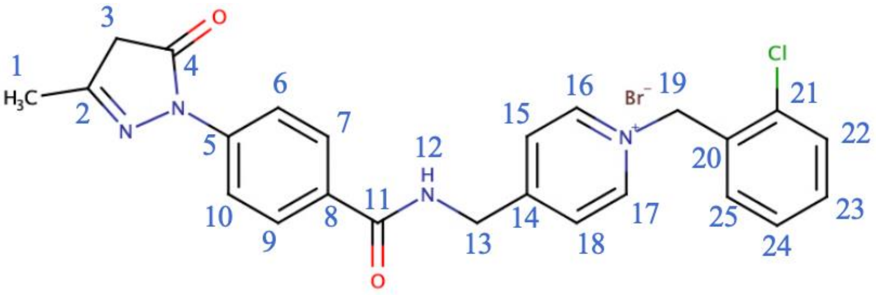

Compound **5e**

Spectrum 22: ^{1}H NMR Compound 5e

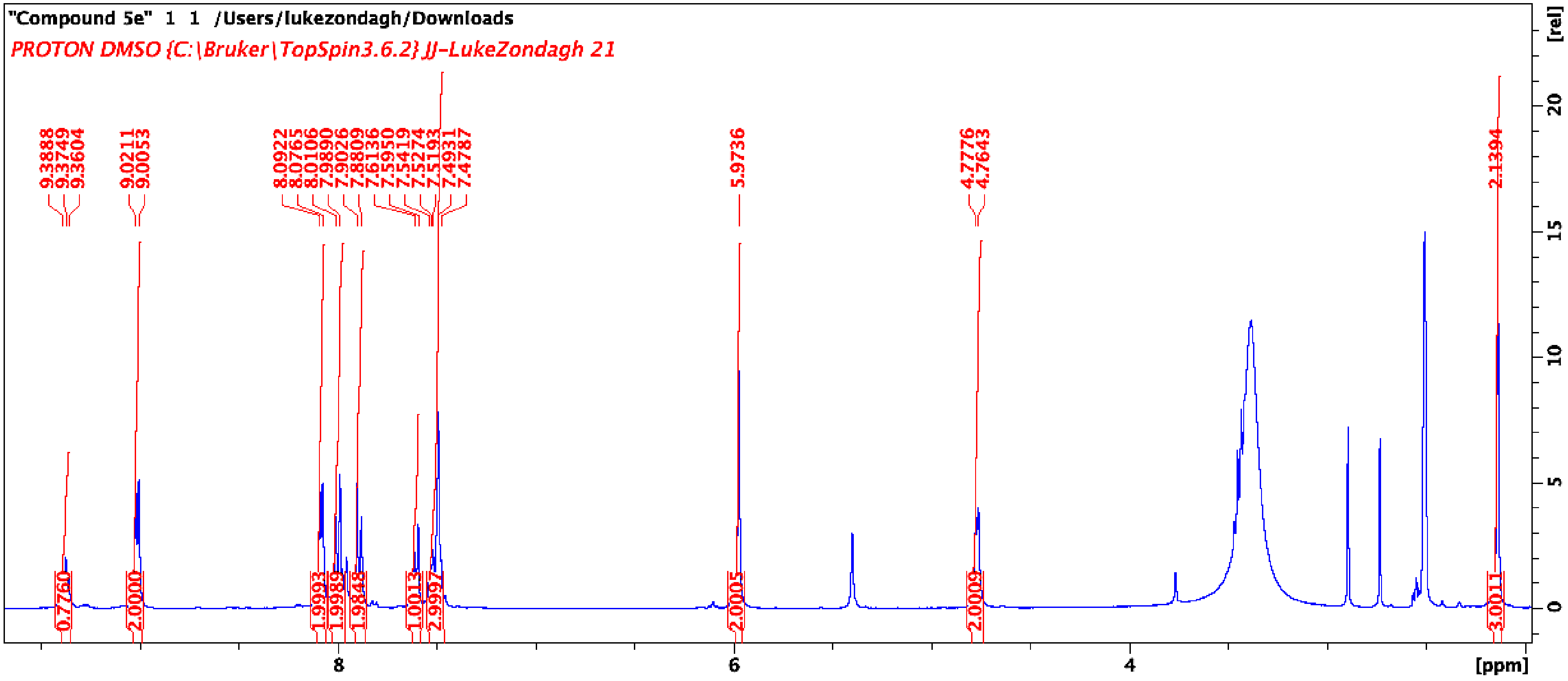

Spectrum 23: ^{13}C NMR Compound 5e

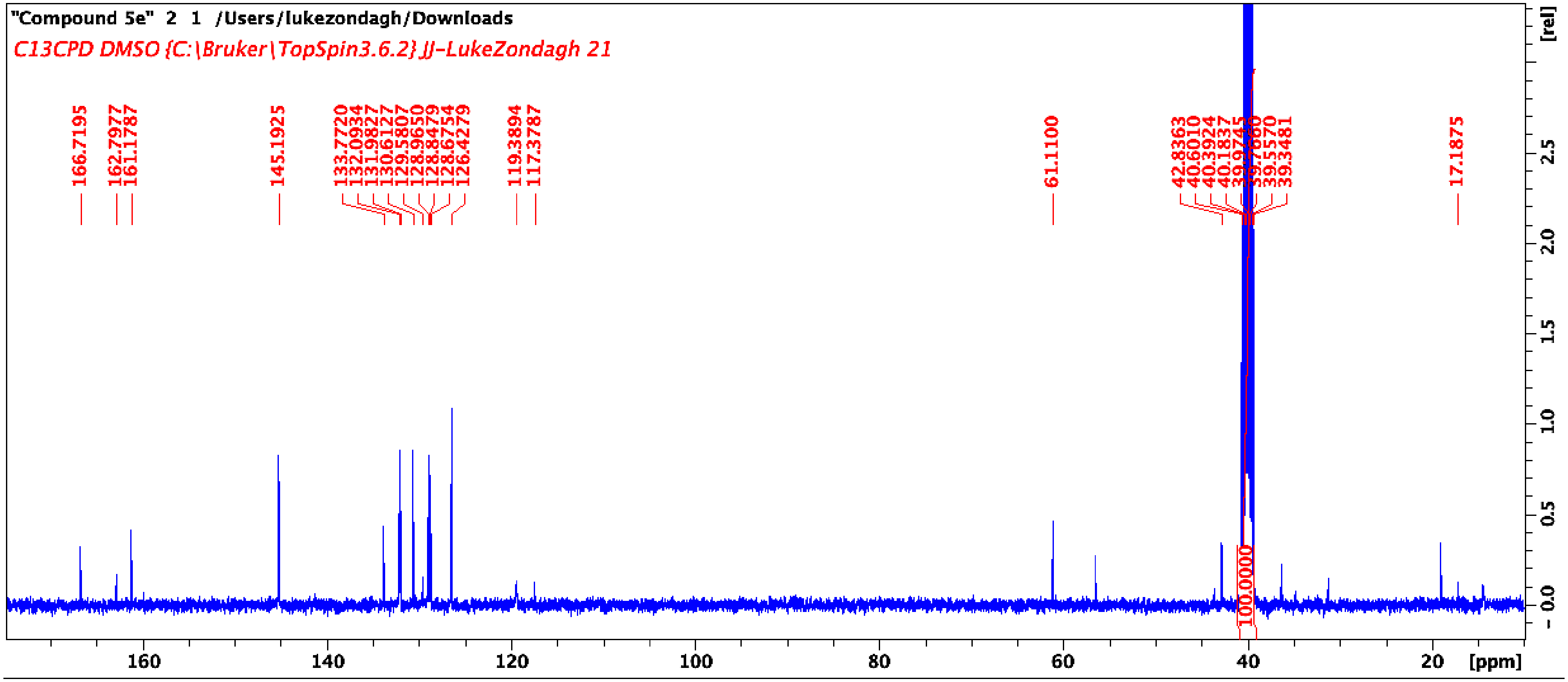

Spectrum 24: IR Compound 5e

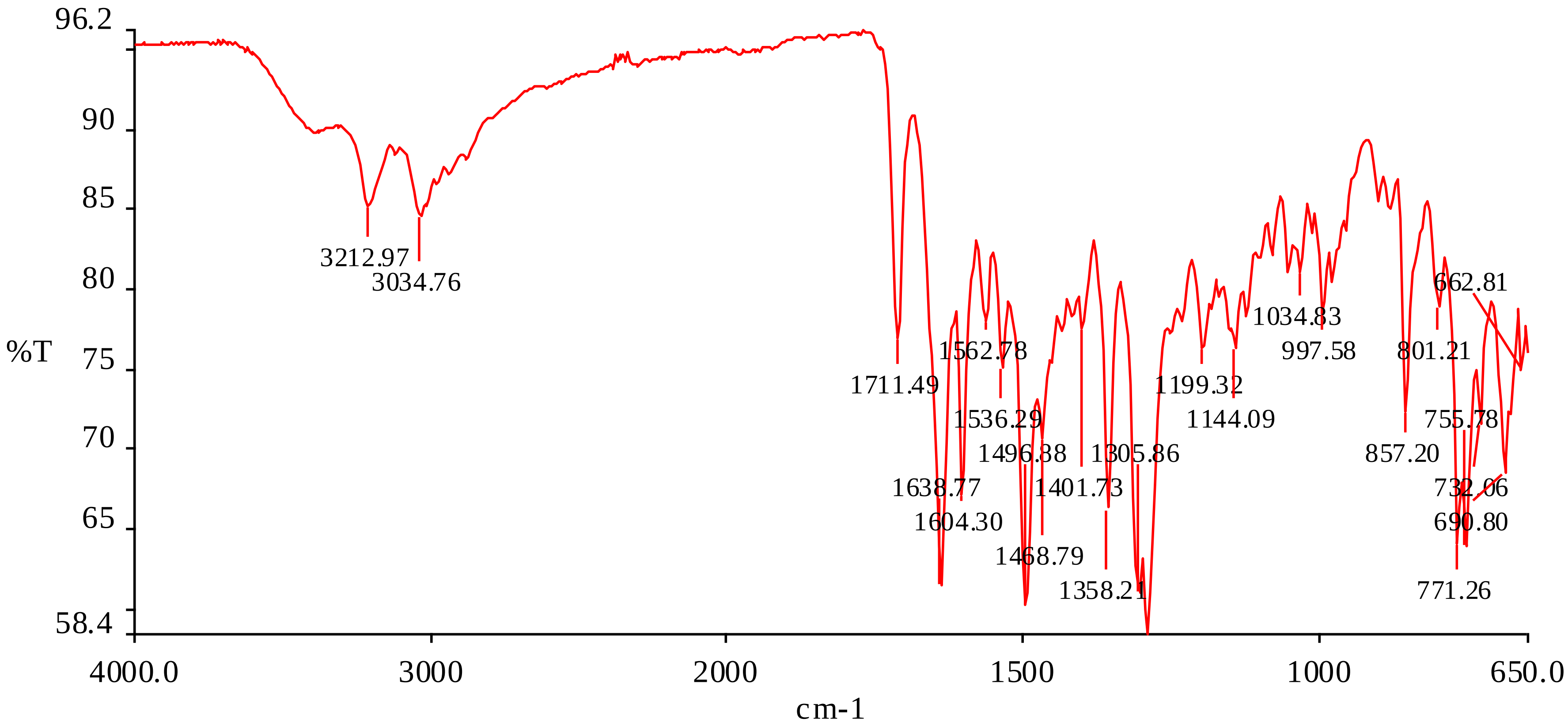

Spectrum 25: MS Compound 5e

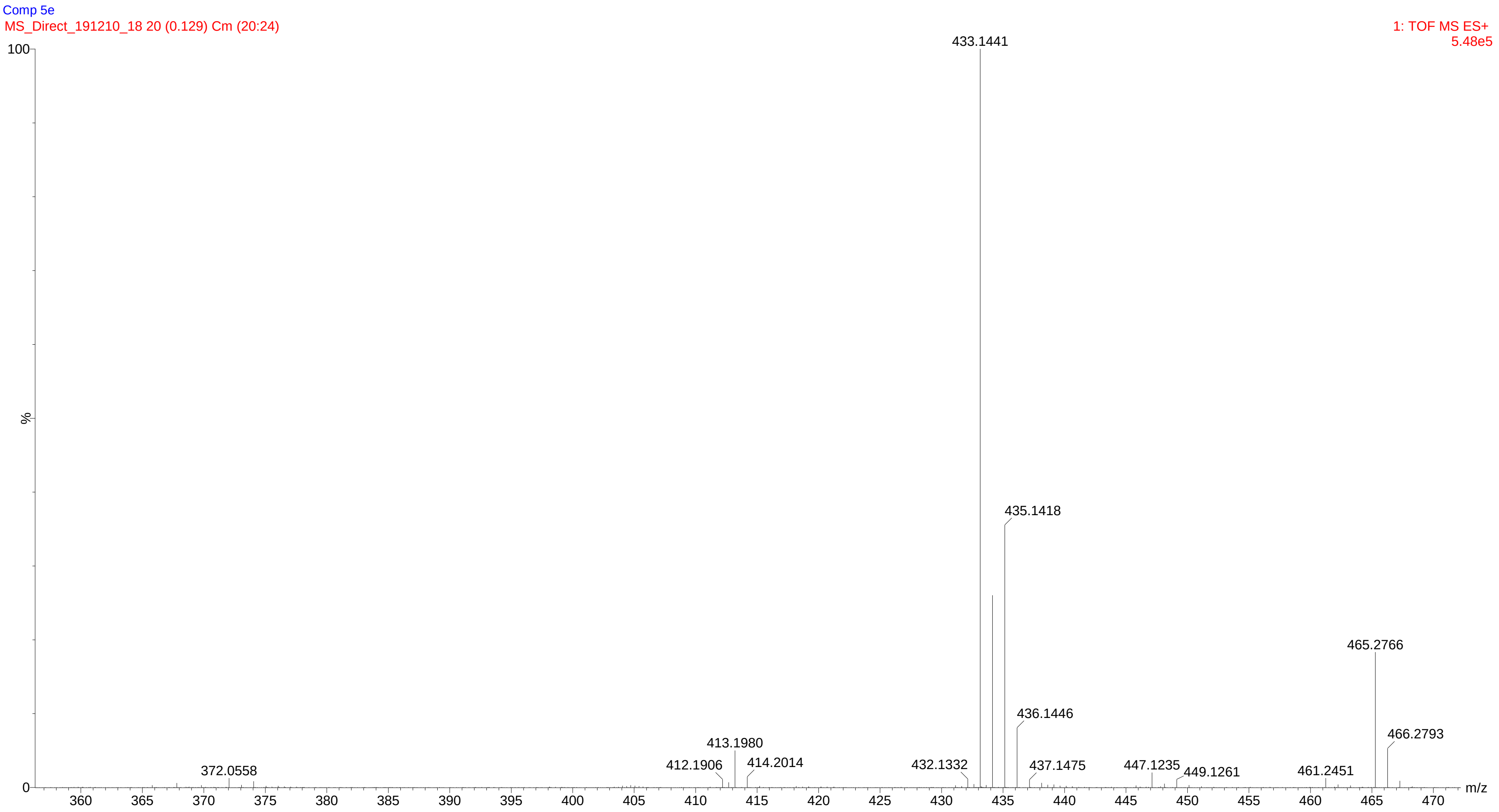

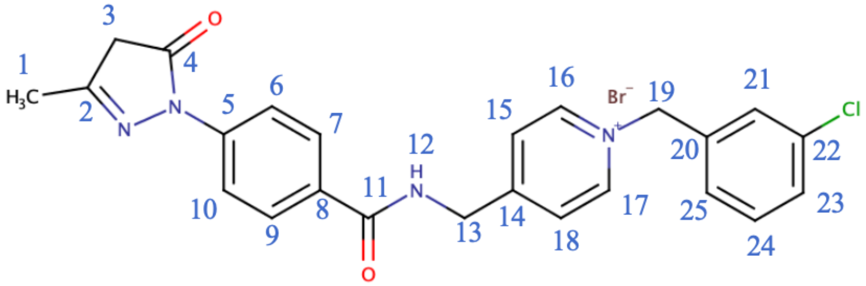

Compound **5f**

<u>Spectrum 26: ^{1}H NMR Compound 5f</u>

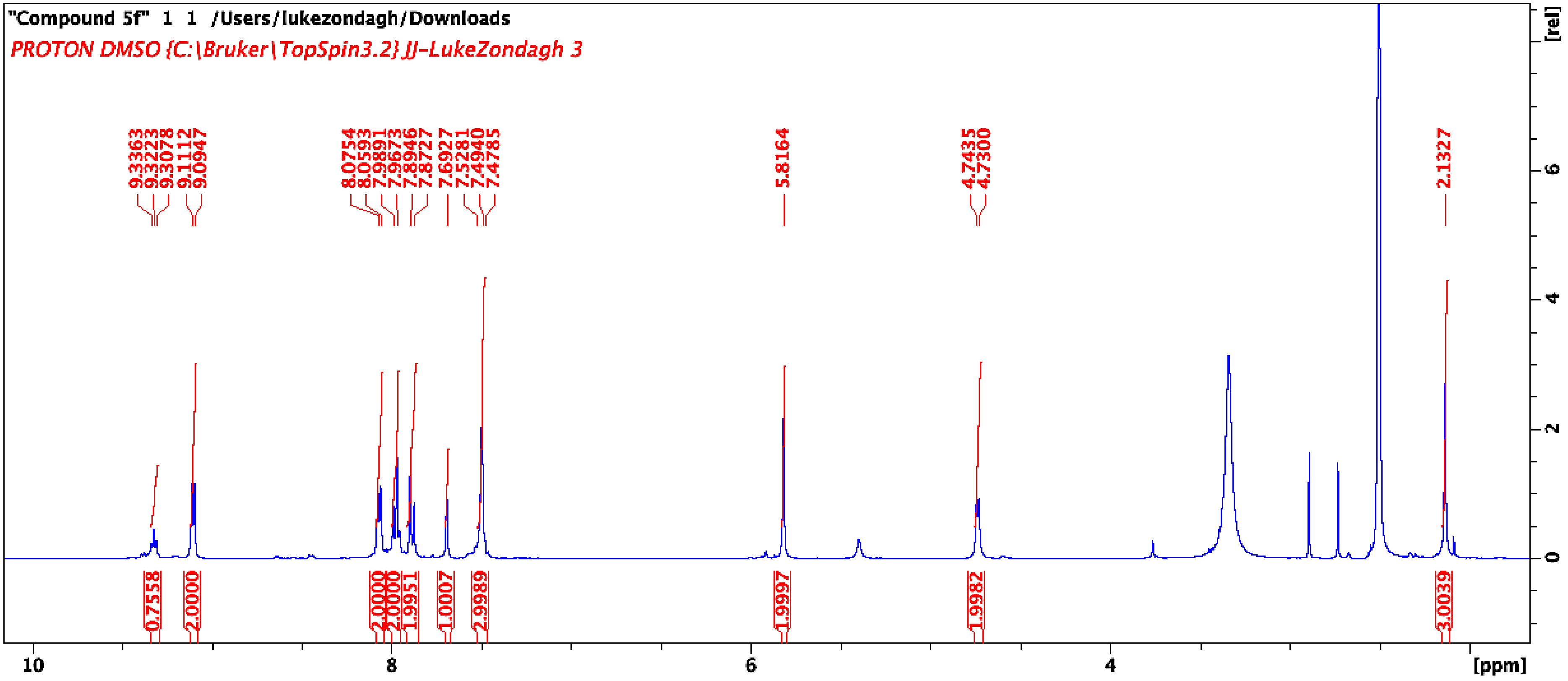

Spectrum 27: ^{13}C NMR Compound 5f

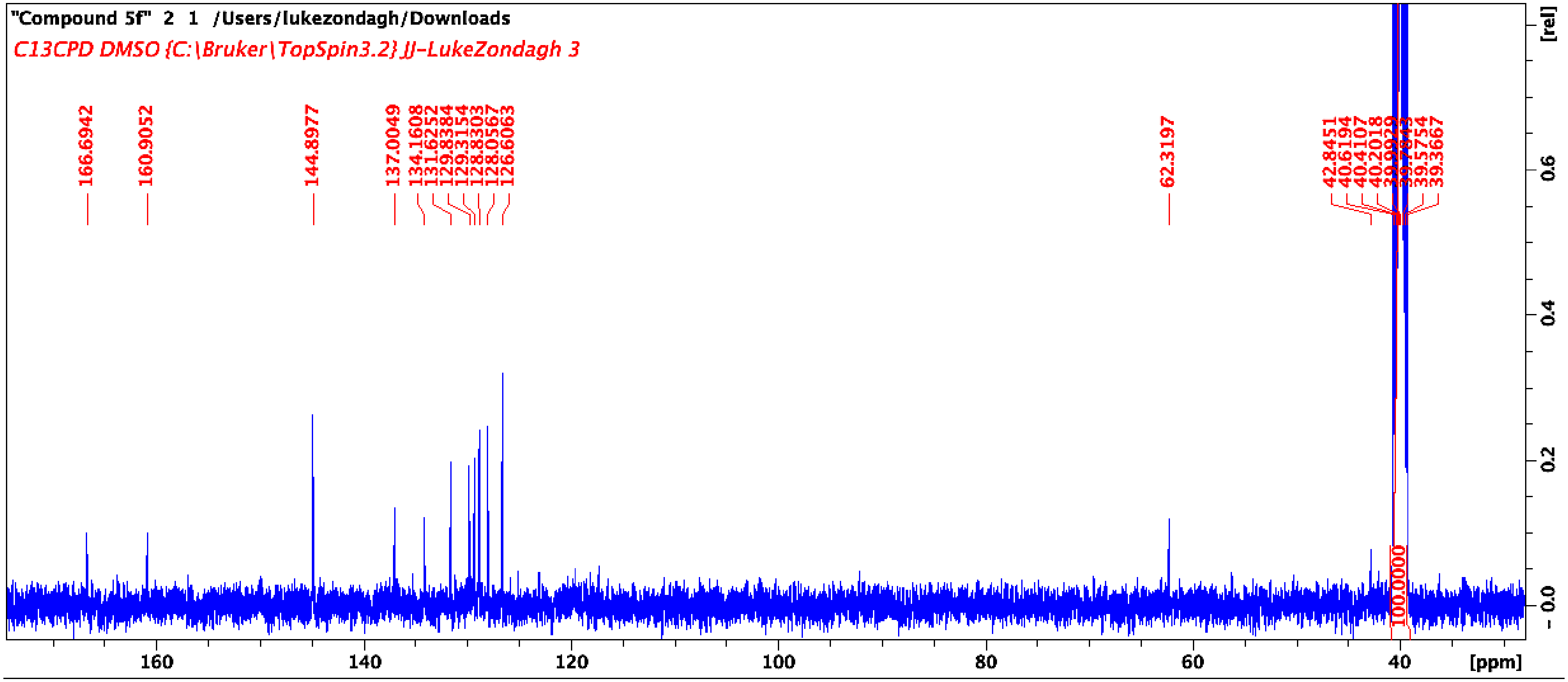

Spectrum 28: HSQC NMR Compound 5f

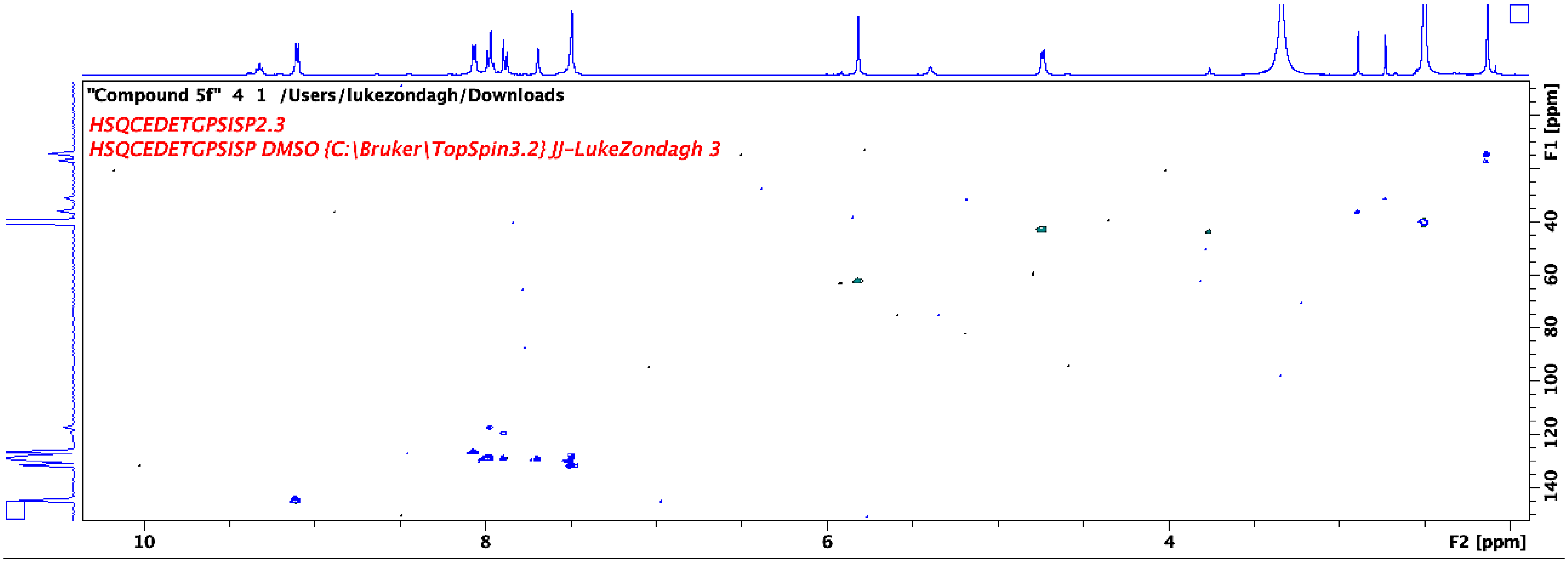

Spectrum 29: IR Compound 5f

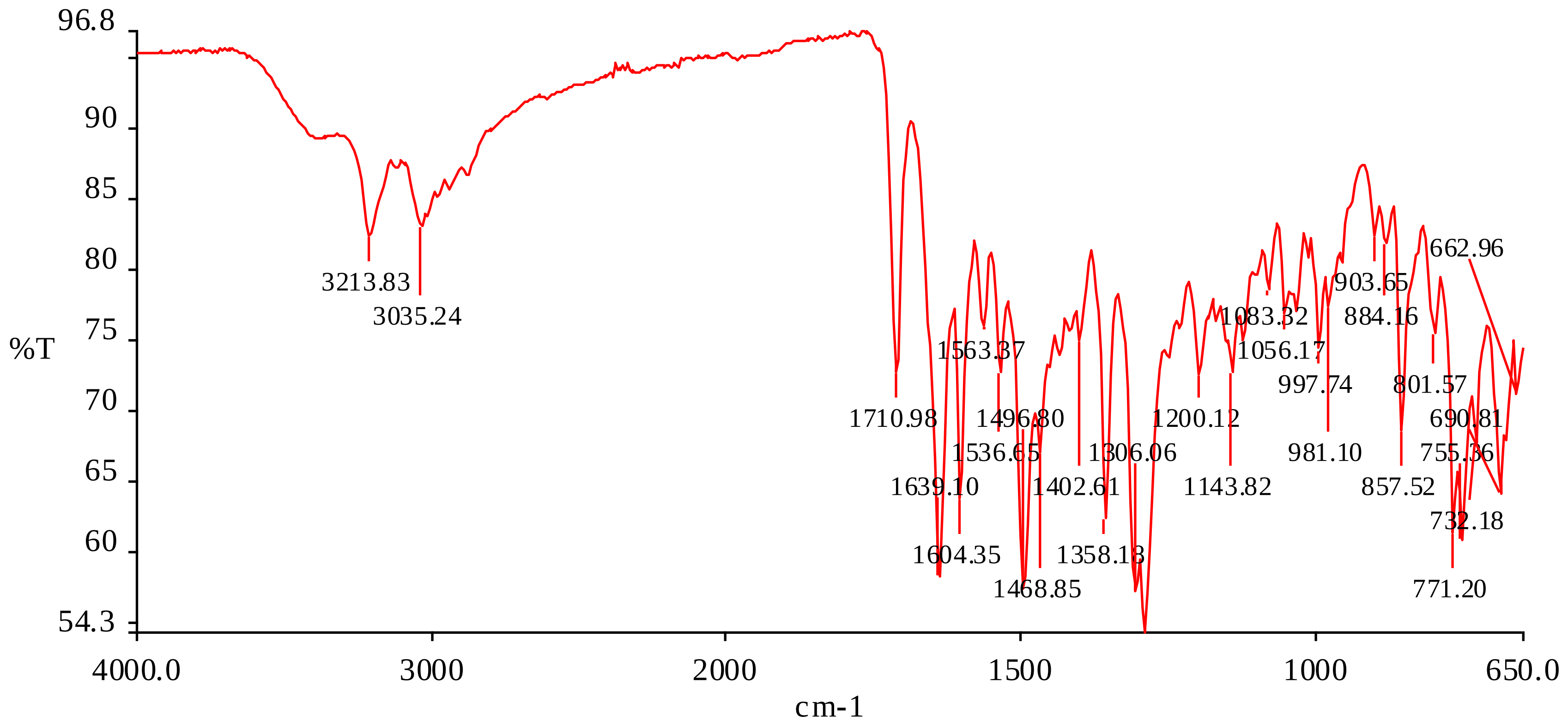

Spectrum 30: MS Compound 5f

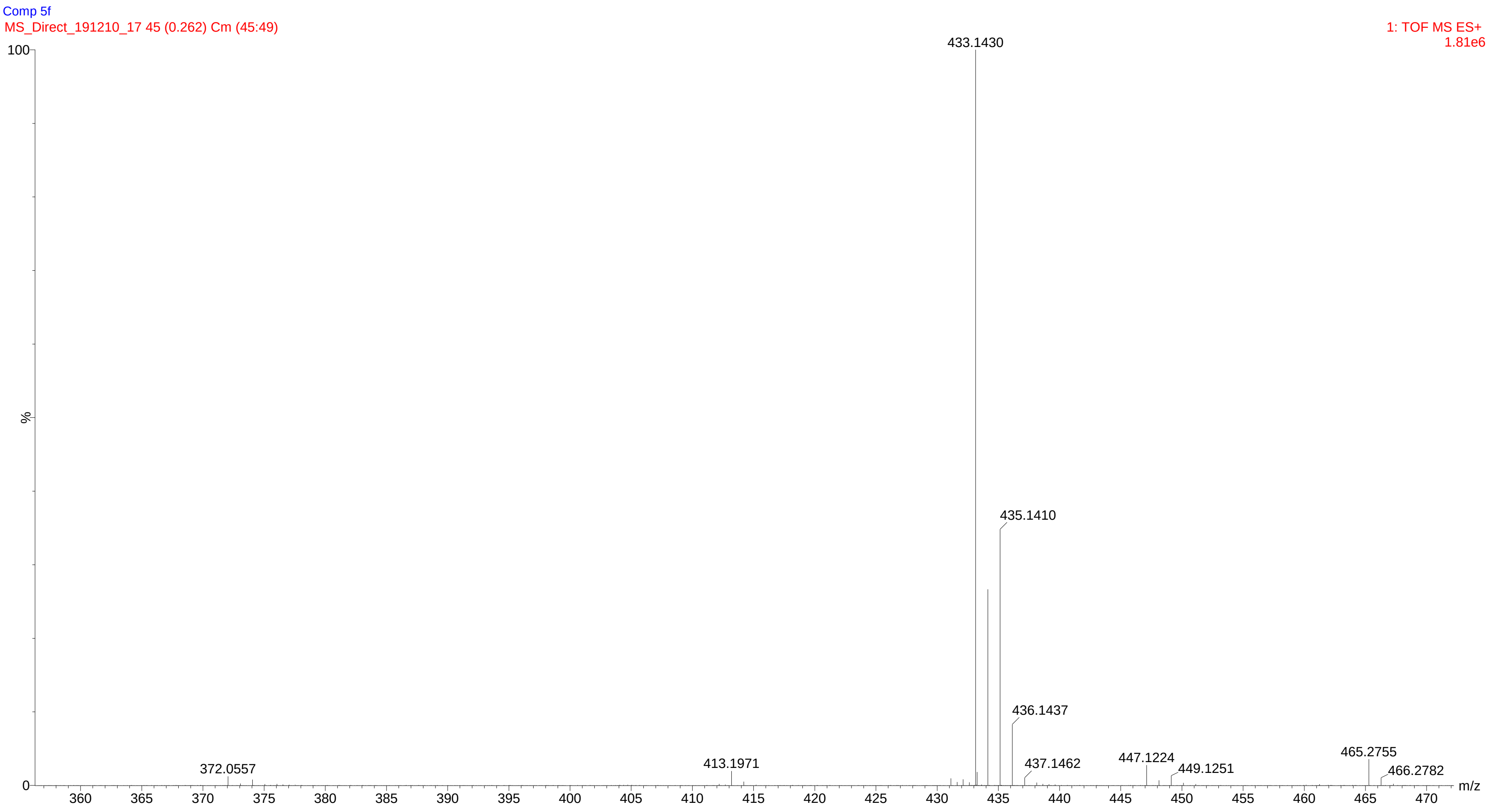

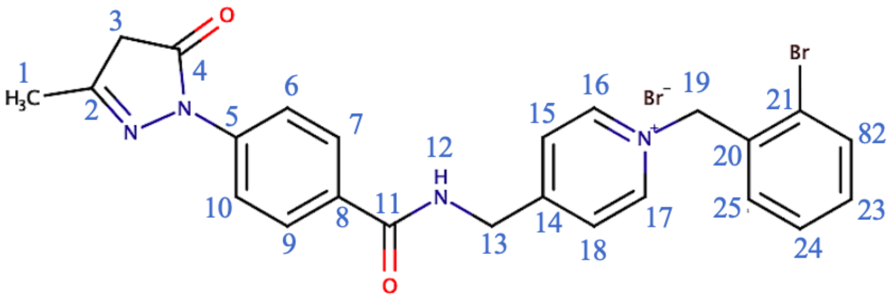

Compound **5g**

Spectrum 31: ^{1}H NMR Compound 5g

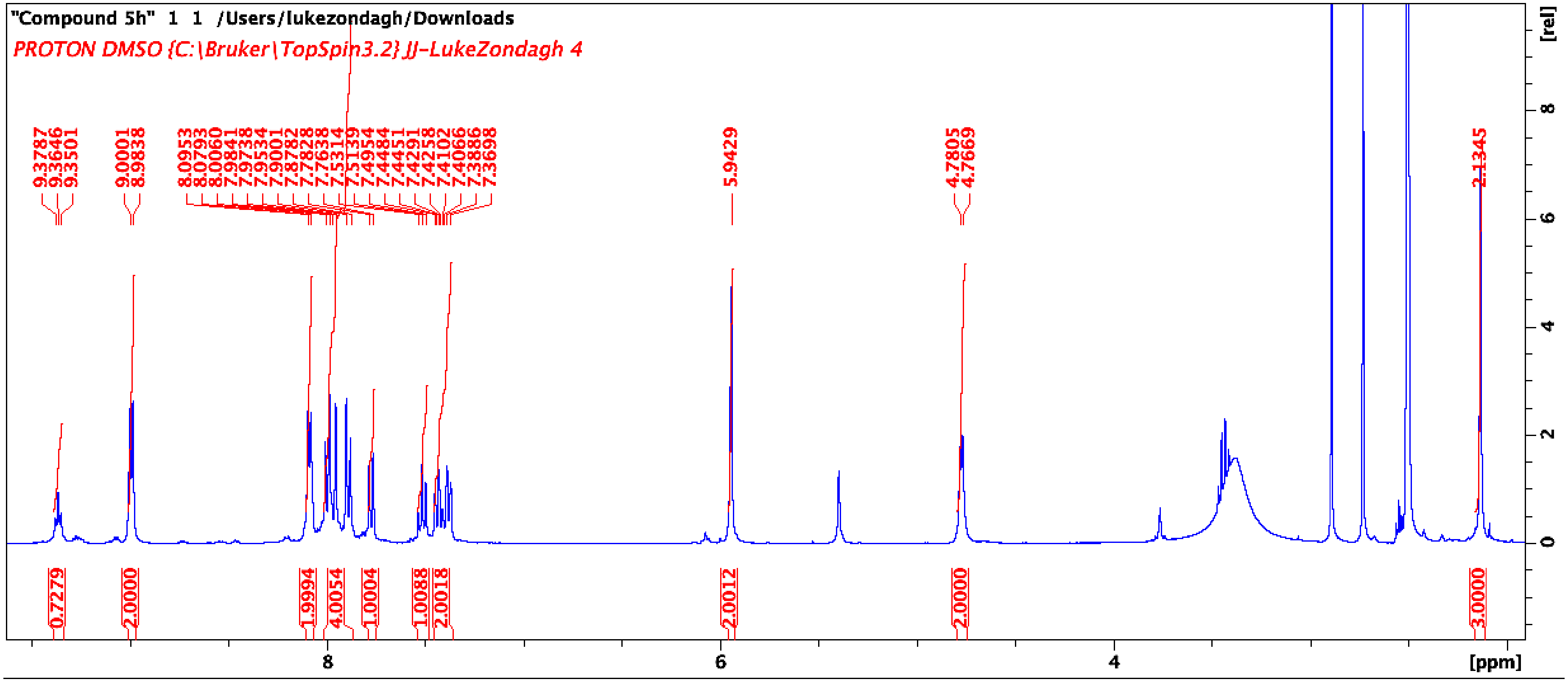

Spectrum 32: ^{13}C NMR Compound 5g

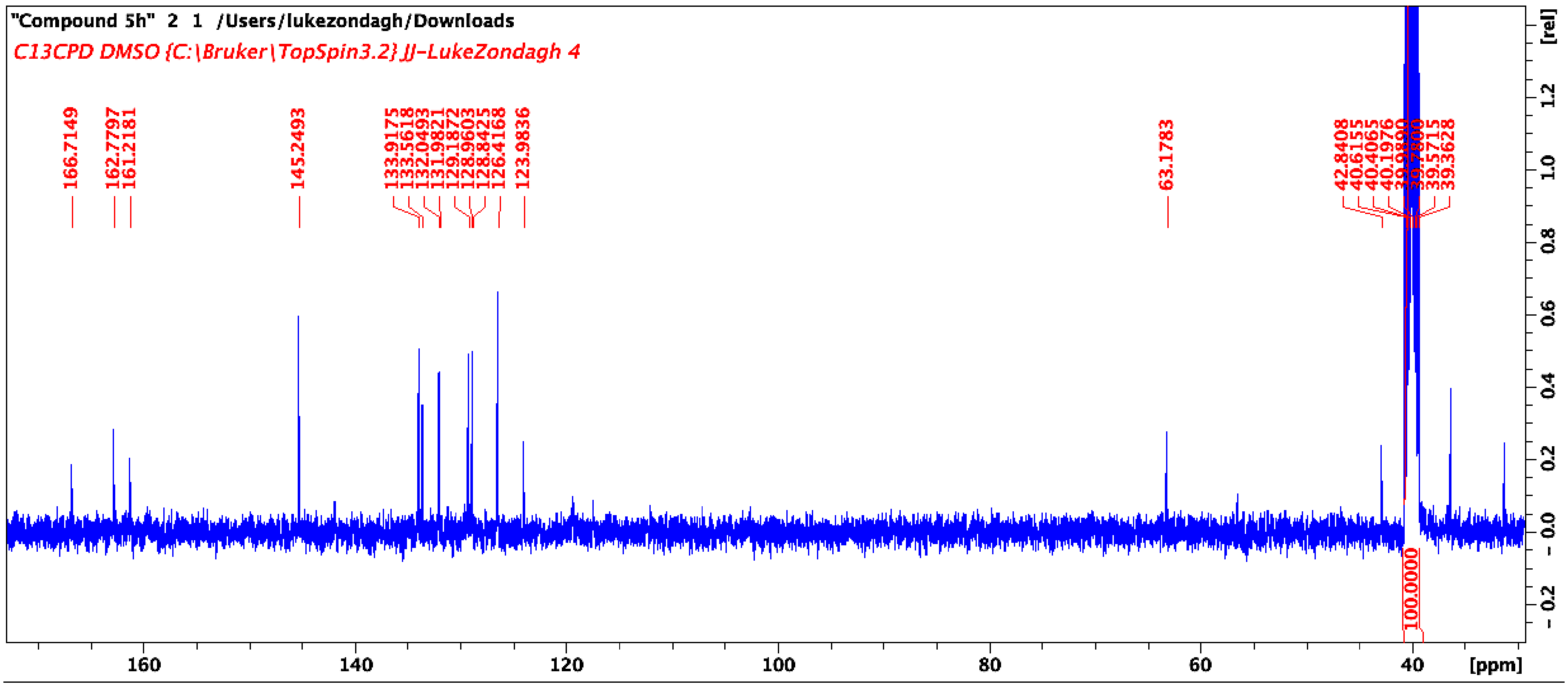

Spectrum 33: HSQC NMR Compound 5g

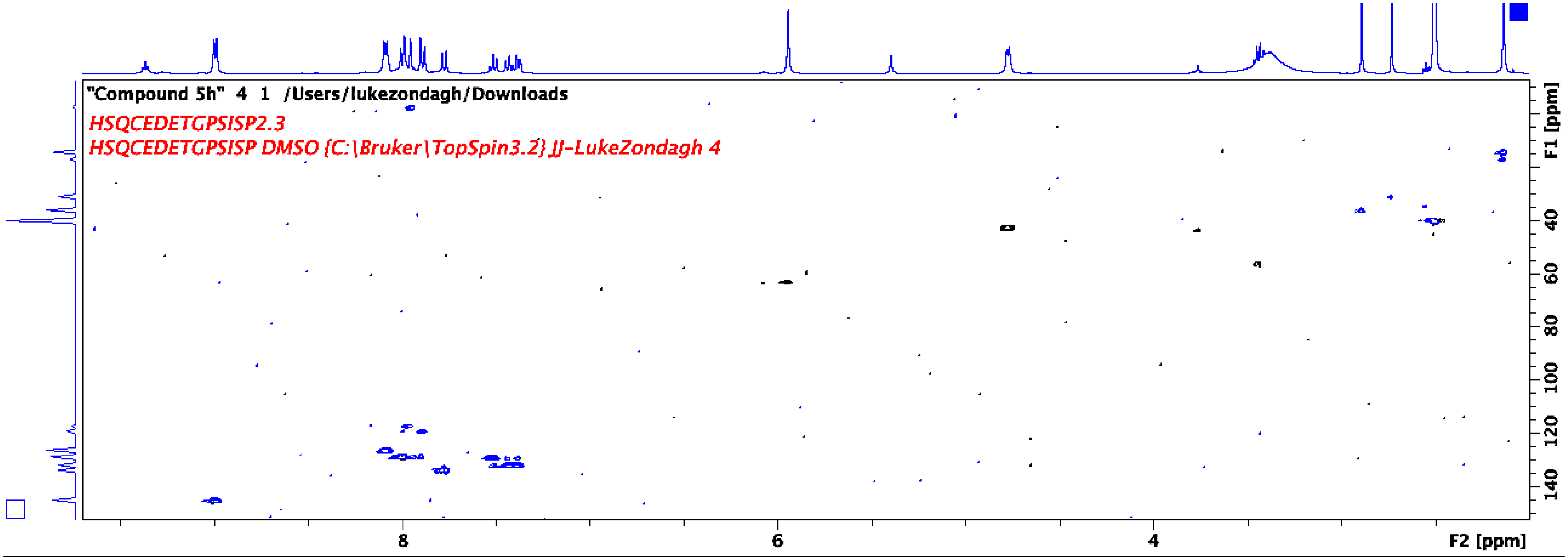

Spectrum 34: IR Compound 5g

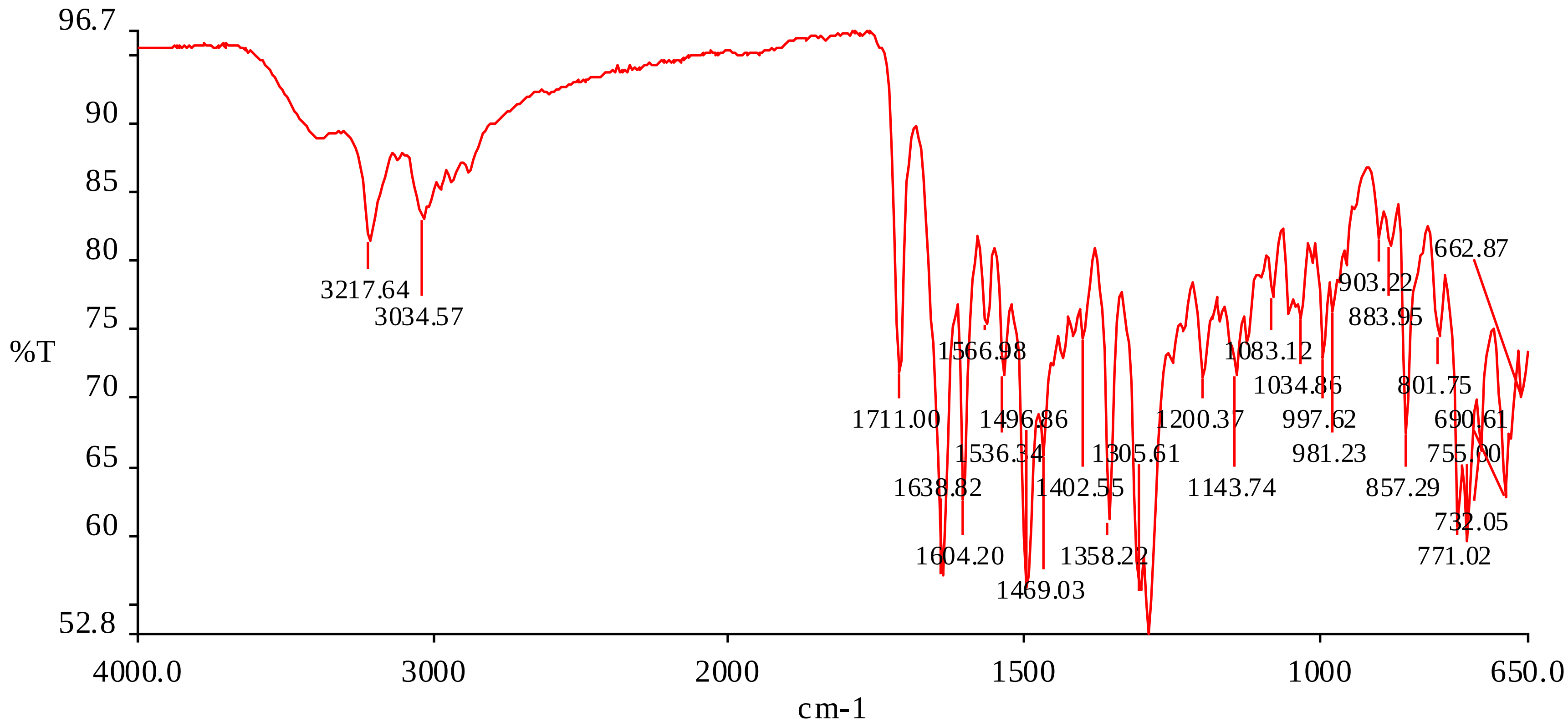

Spectrum 35: MS Compound 5g

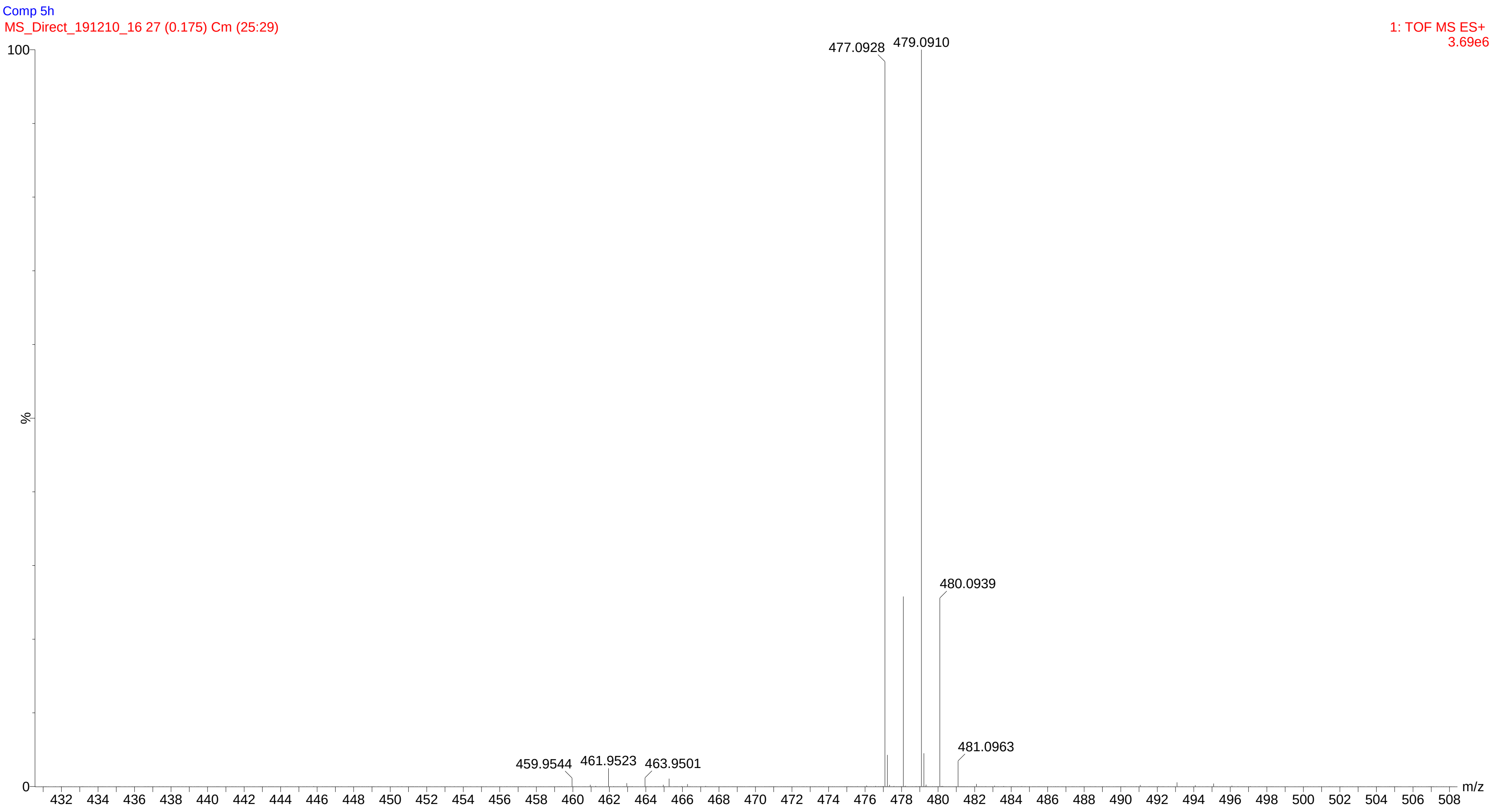

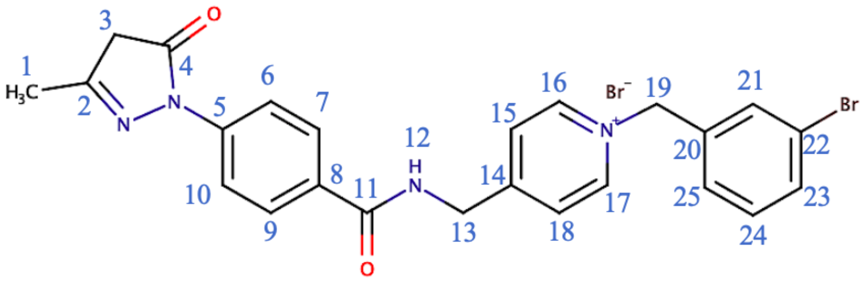

Compound **5h**

Spectrum 36: ^{1}H NMR Compound 5h

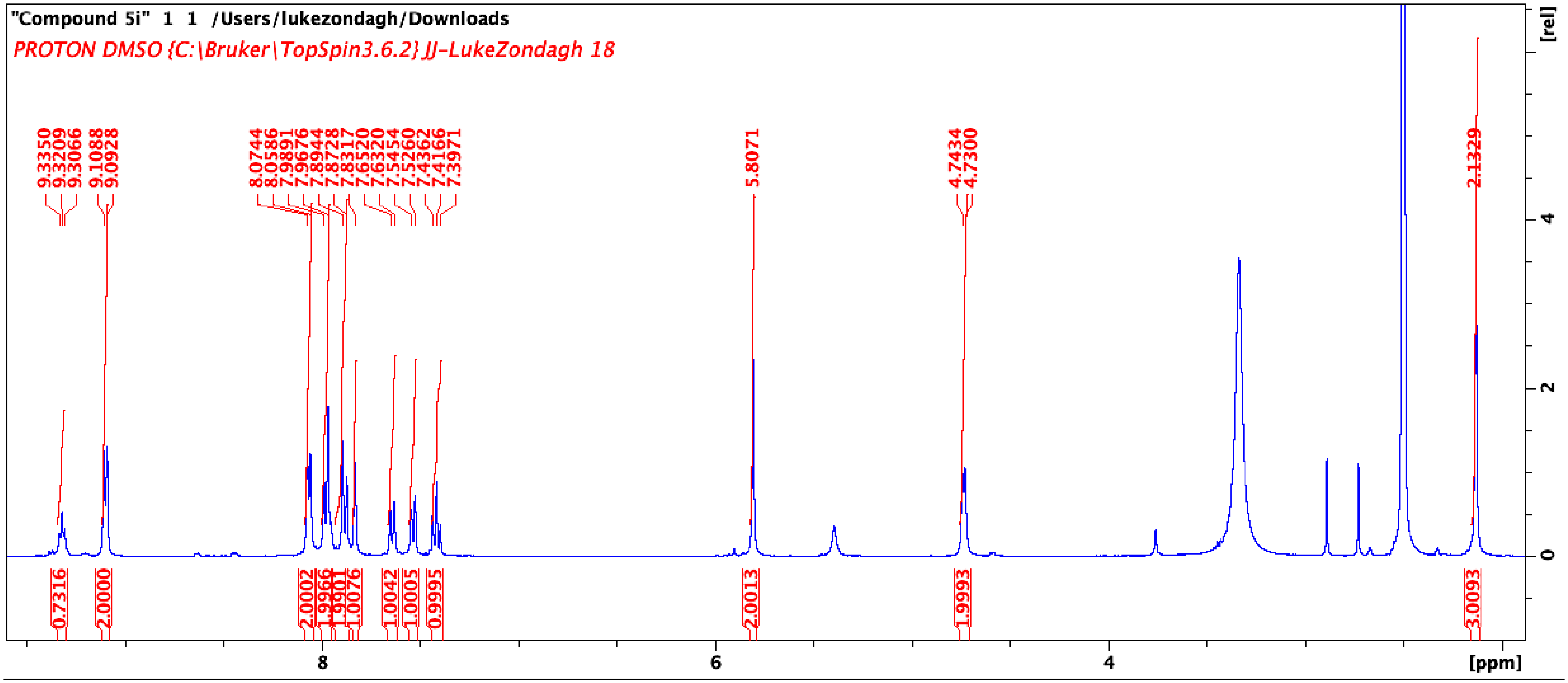

Spectrum 37: ^{13}C NMR Compound 5h

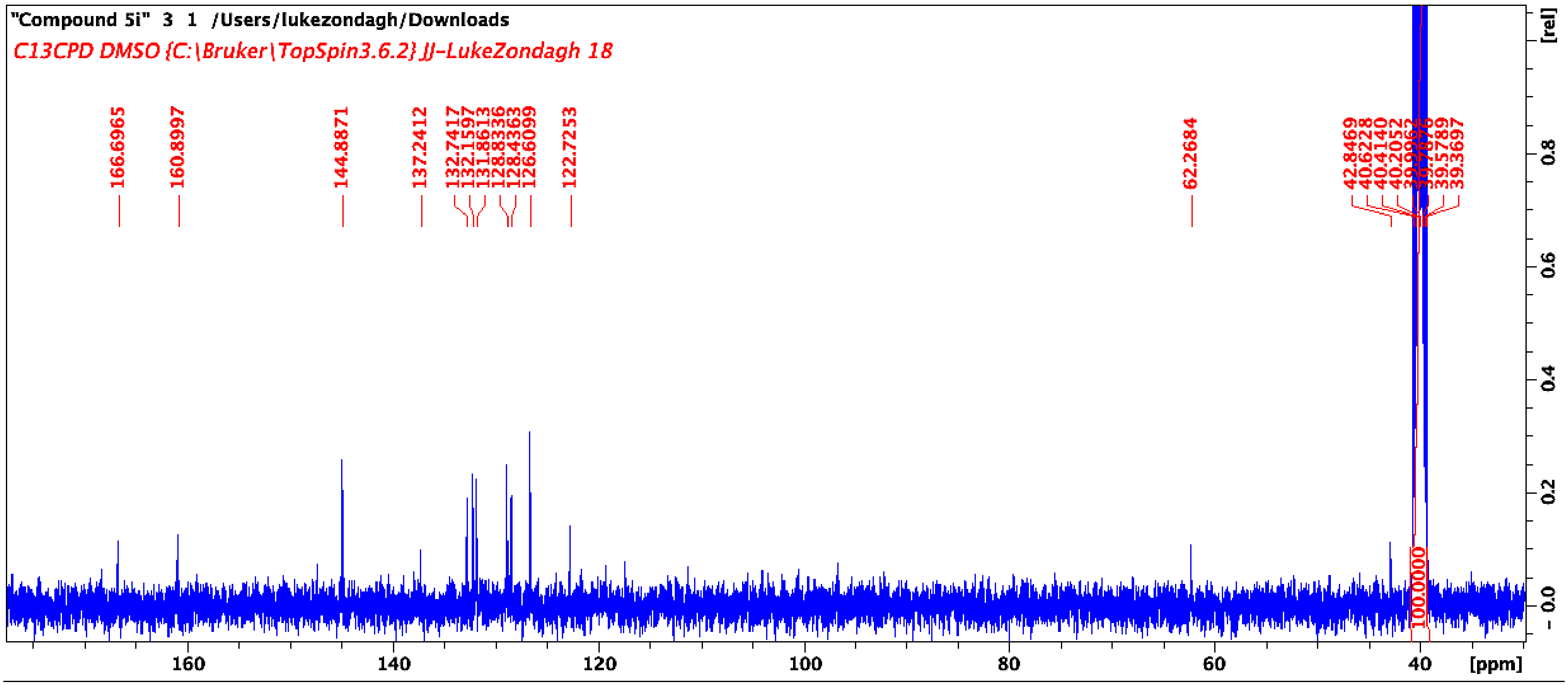

Spectrum 38: IR Compound 5h

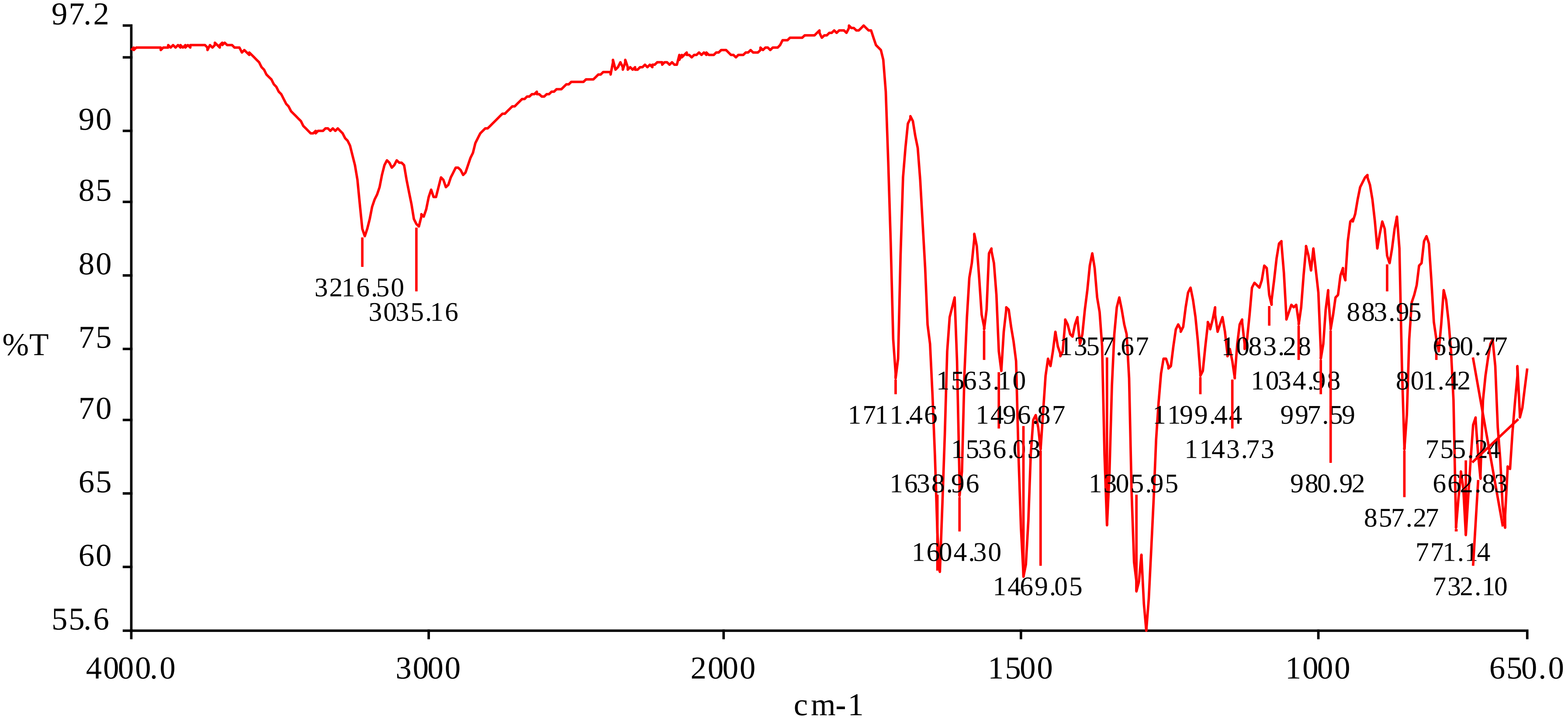

<u>Spectrum 39: MS Compound 5h</u>

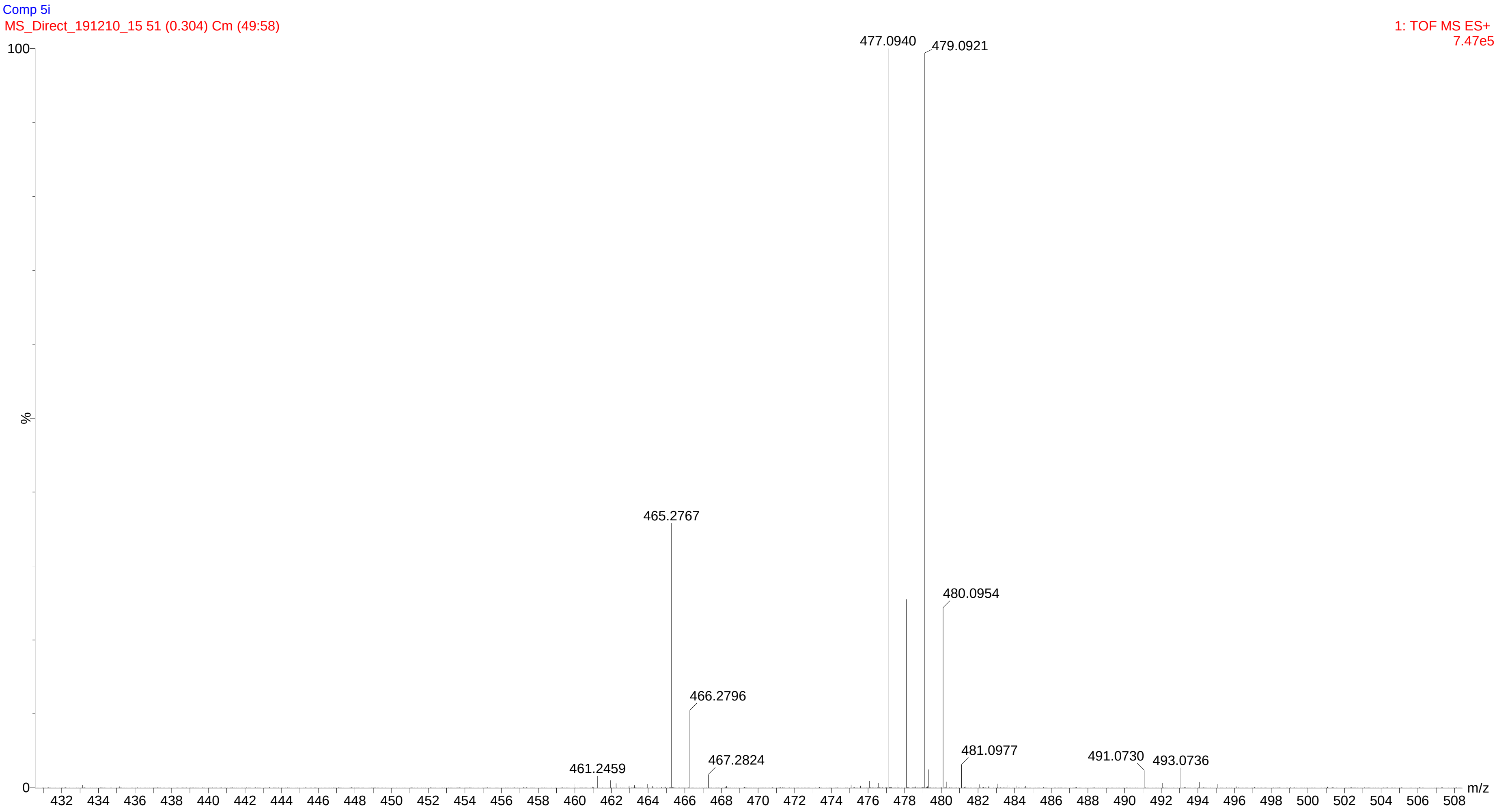

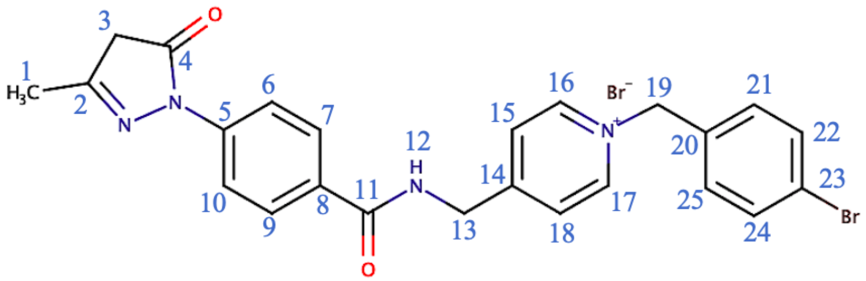

Compound **5i**

Spectrum 40: ^{1}H NMR Compound 5i

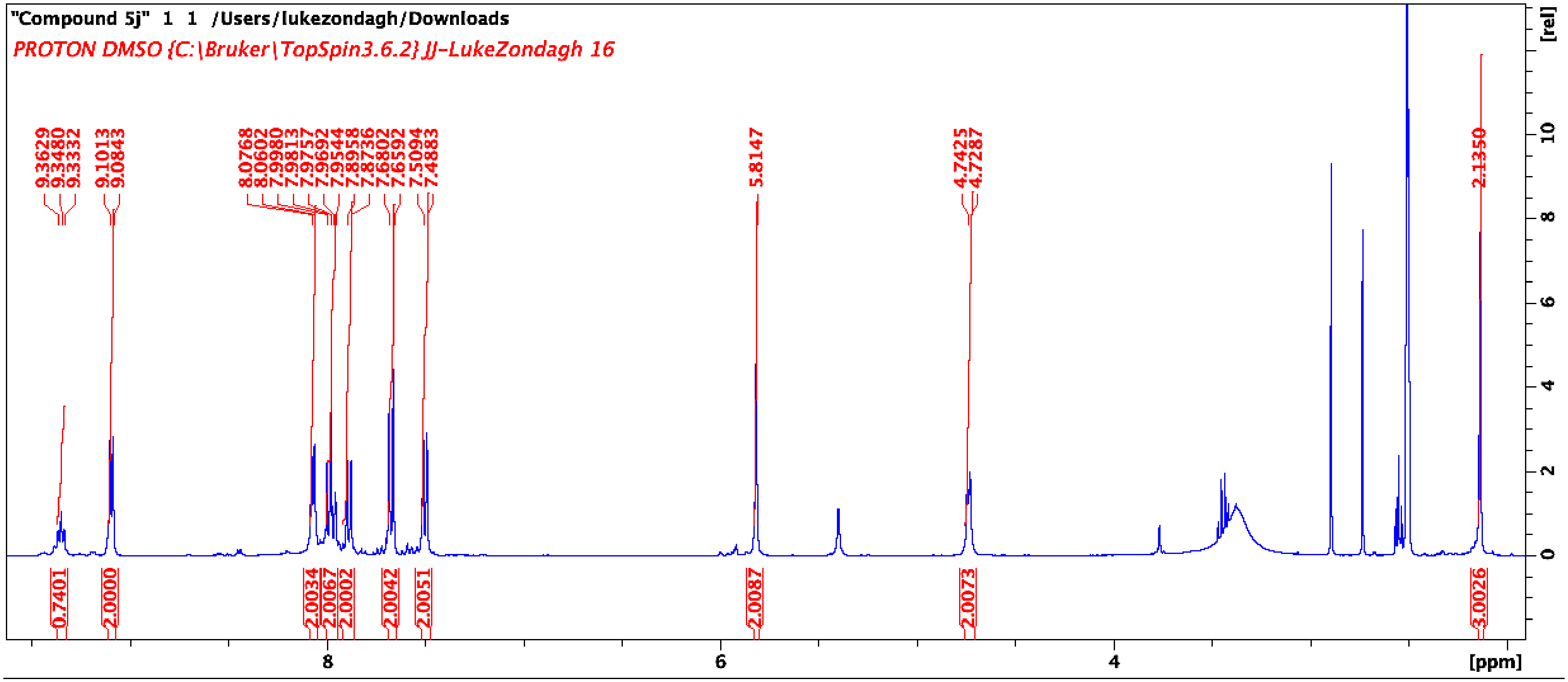

Spectrum 41: ^{13}C NMR Compound 5i

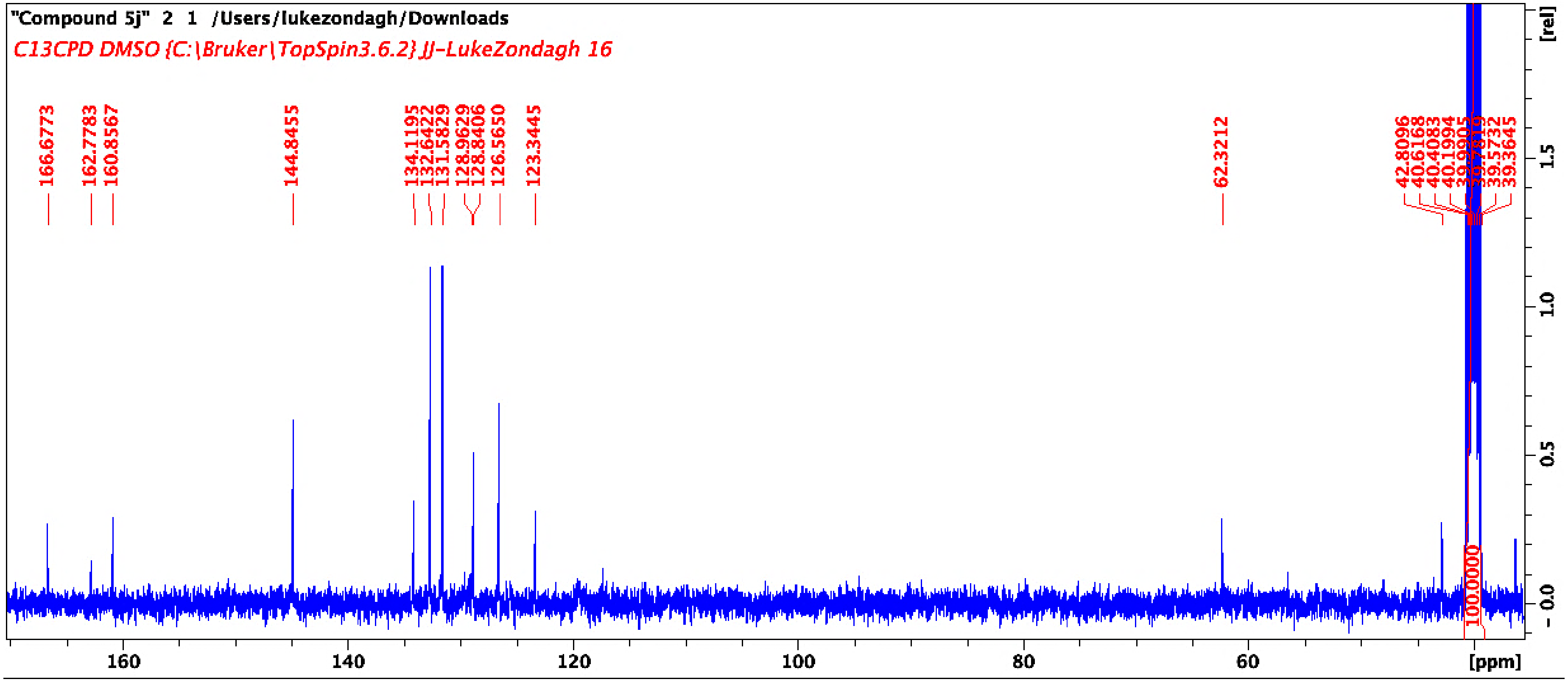

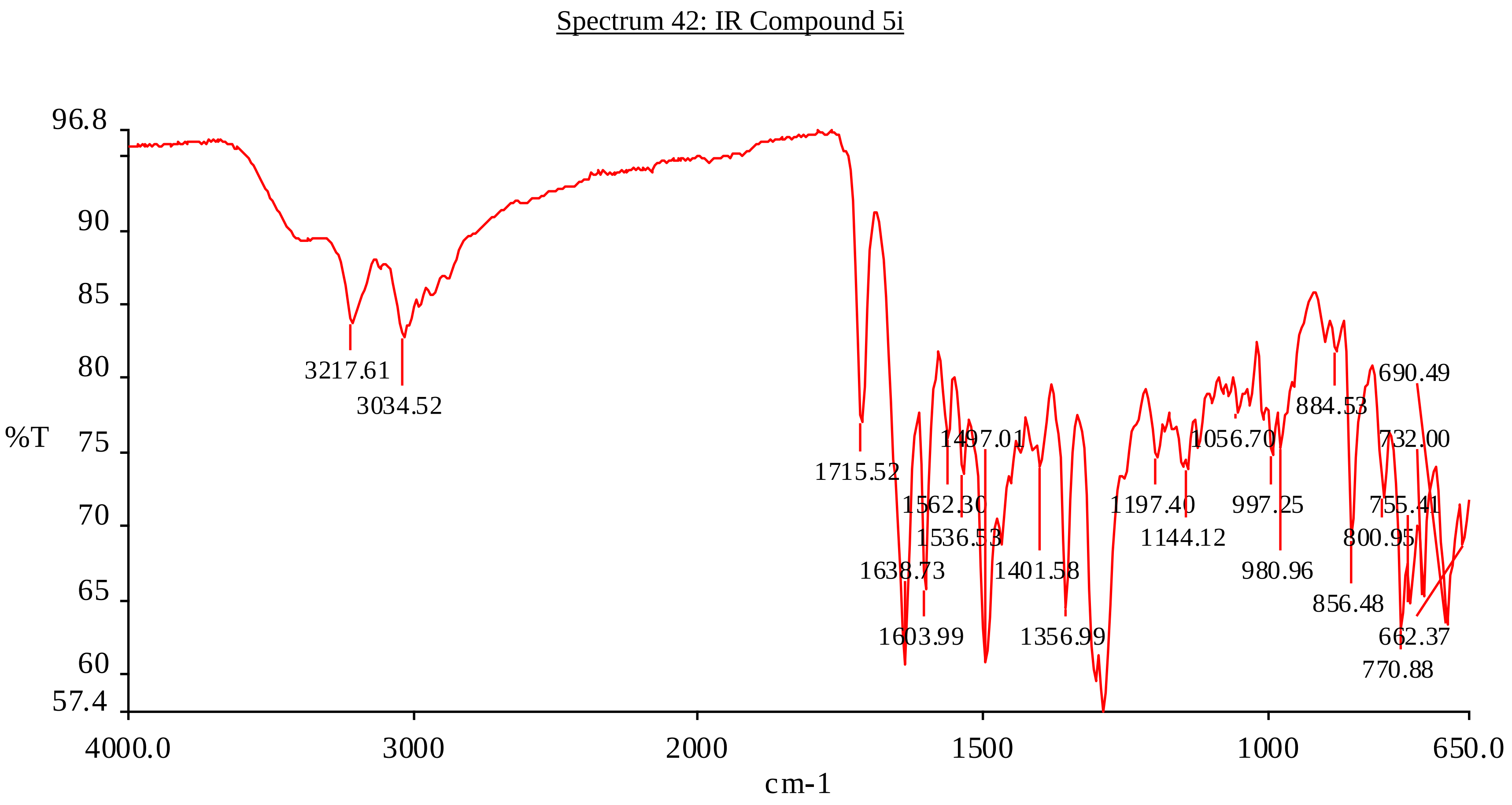
Spectrum 42: IR Compound 5i
96.8
90
85
80
75
70
65
60
57.4
%T
4000.0
3000
2000
1500
1000
650.0
cm-1
3217.61
3034.52
1715.52
1638.73
1603.99
1562.30
1536.53
1497.01
1401.58
1356.99
1197.40
1144.12
1056.70
997.25
980.96
884.53
856.48
800.95
770.88
755.41
732.00
690.49
662.37

Spectrum 43: MS Compound 5i

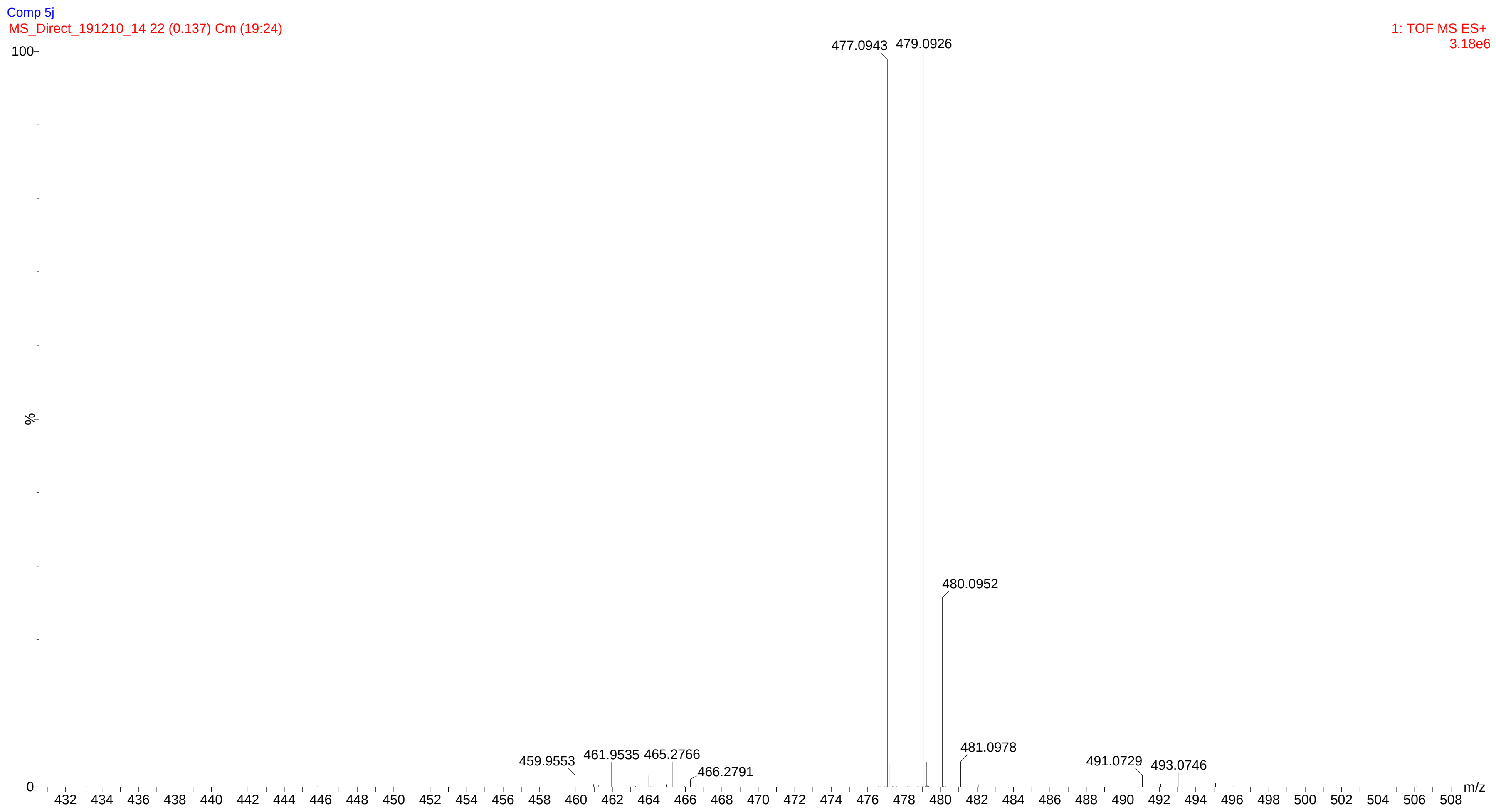

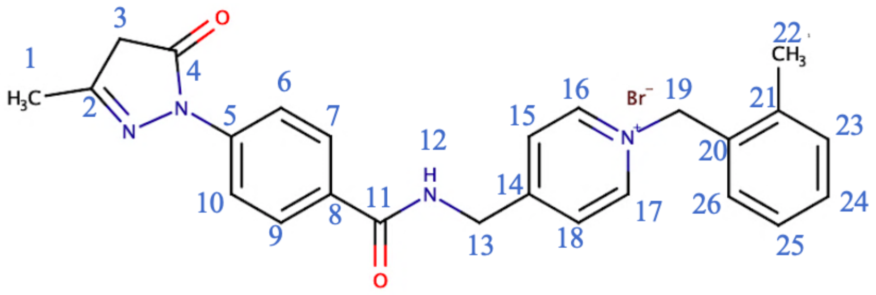

Compound **5j**

<u>Spectrum 44: ^{1}H NMR Compound 5j</u>

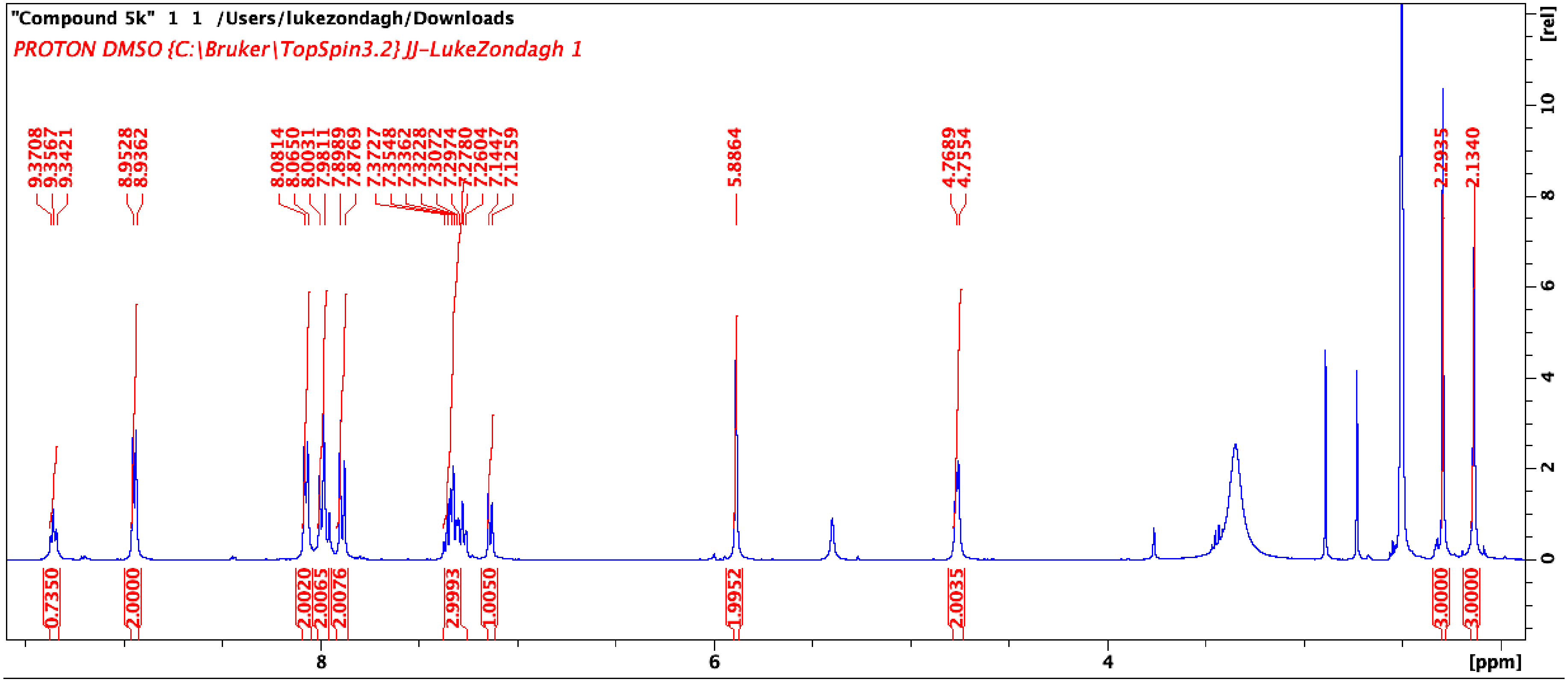

Spectrum 45: ^{13}C NMR Compound 5j

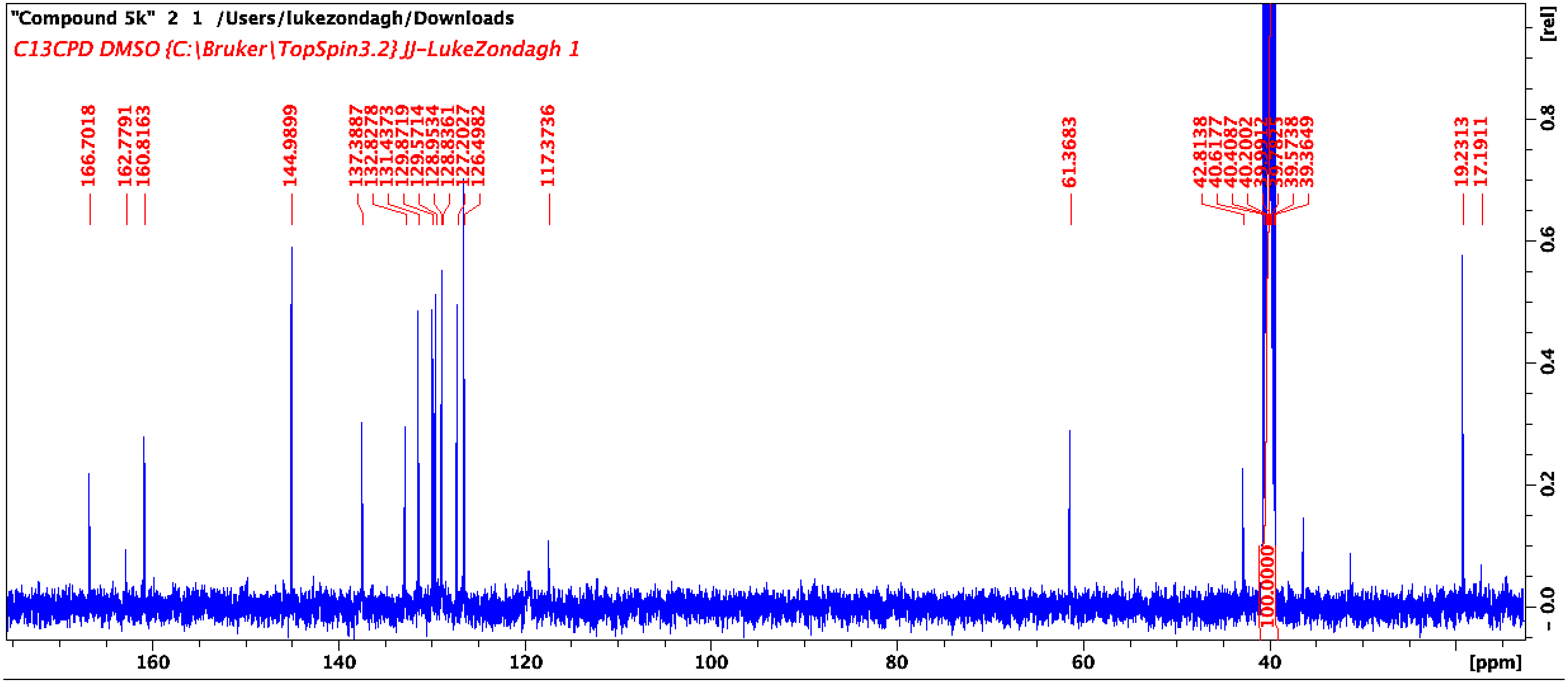

Spectrum 46: IR Compound 5j

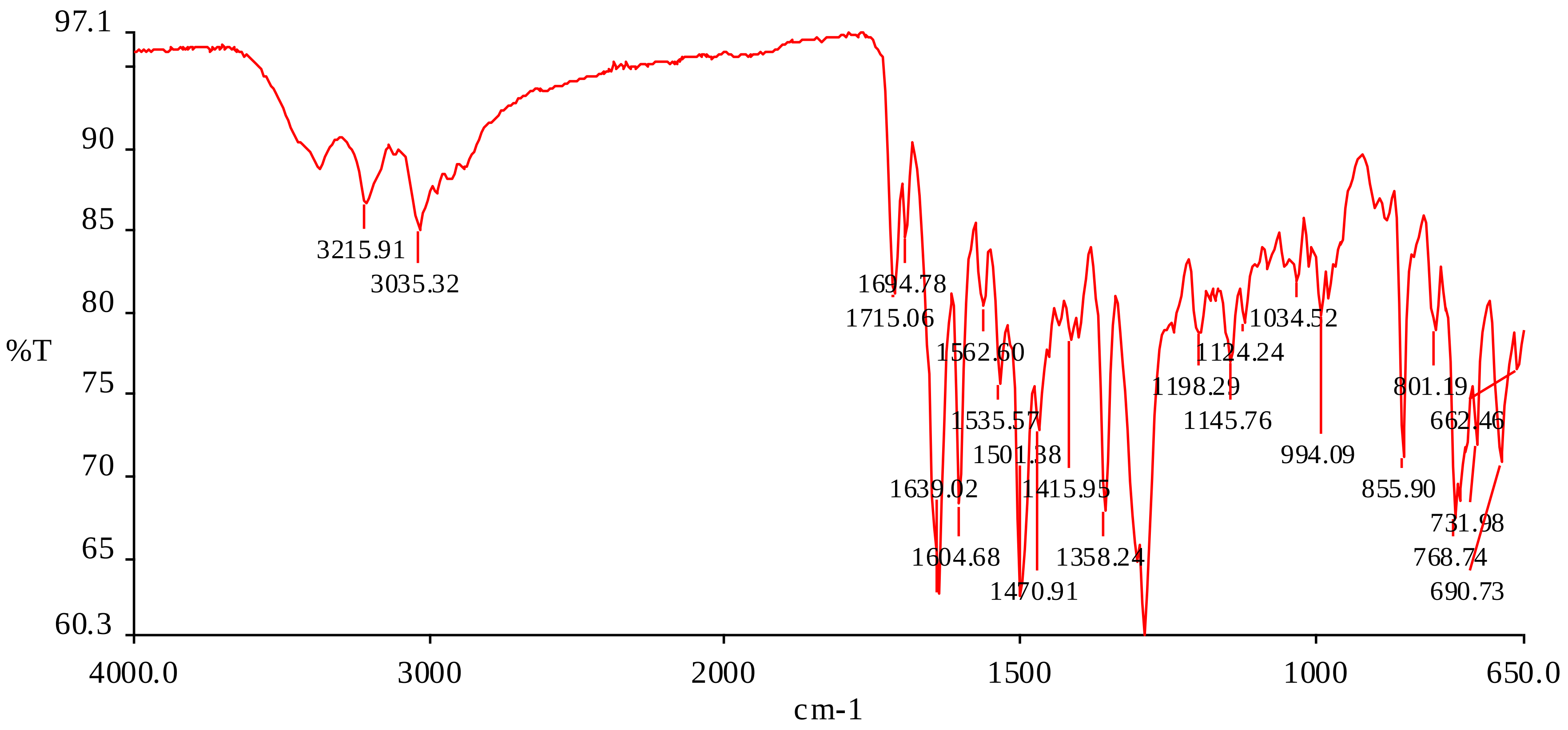

Spectrum 47: MS Compound 5j

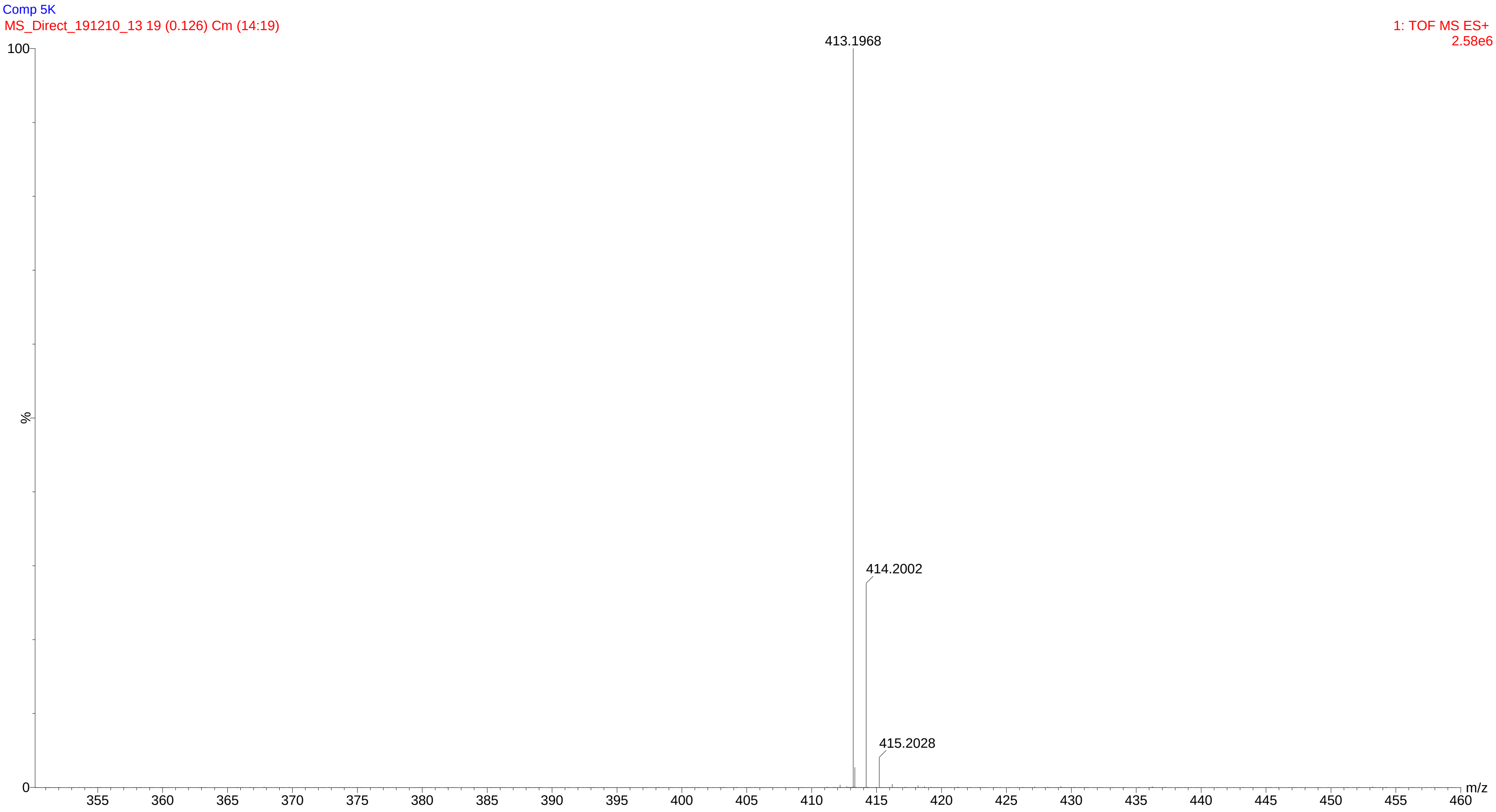

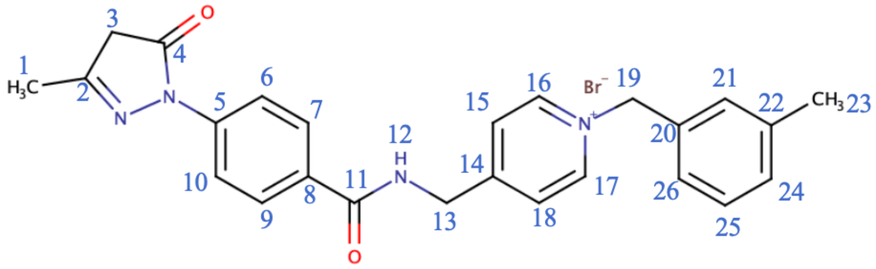

Compound **5k**

Spectrum 48: ^{1}H NMR Compound 5k

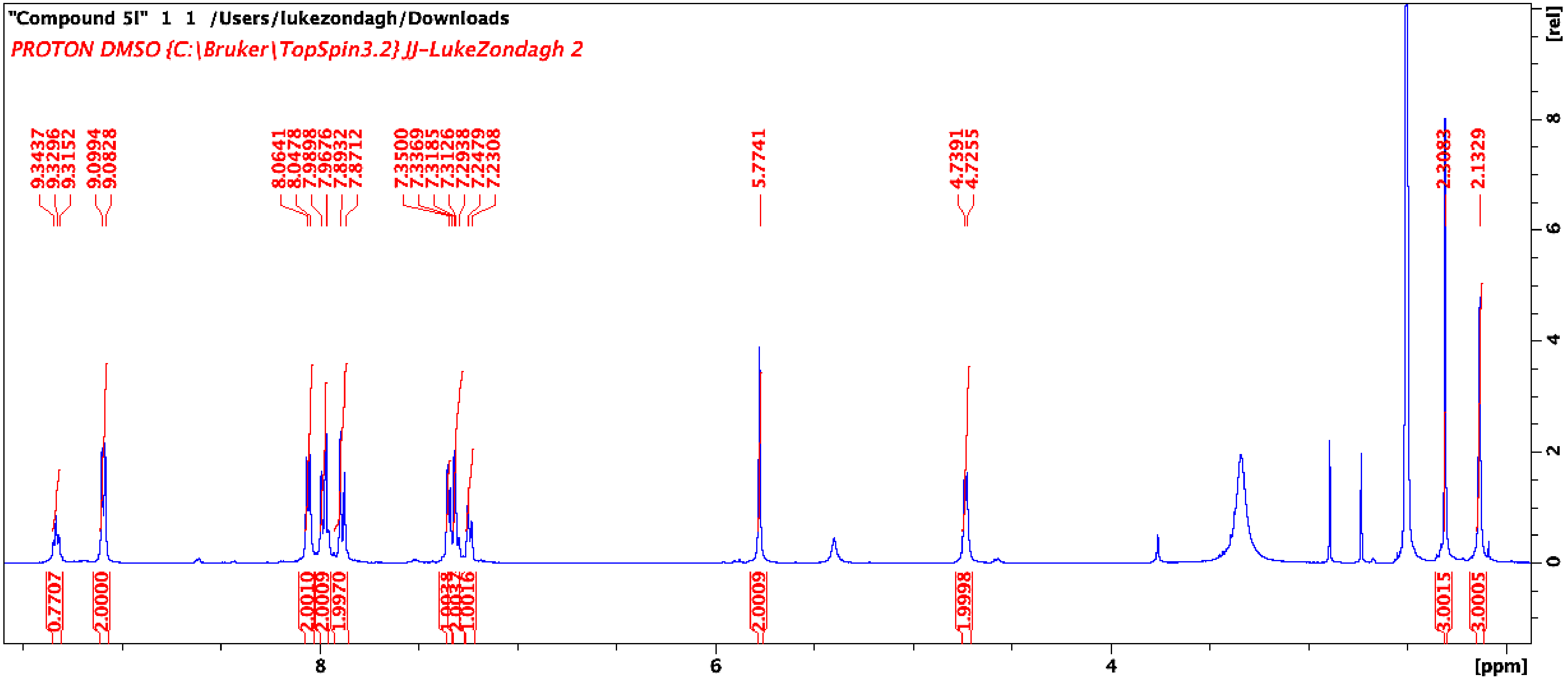

Spectrum 49: ^{13}C NMR Compound 5k

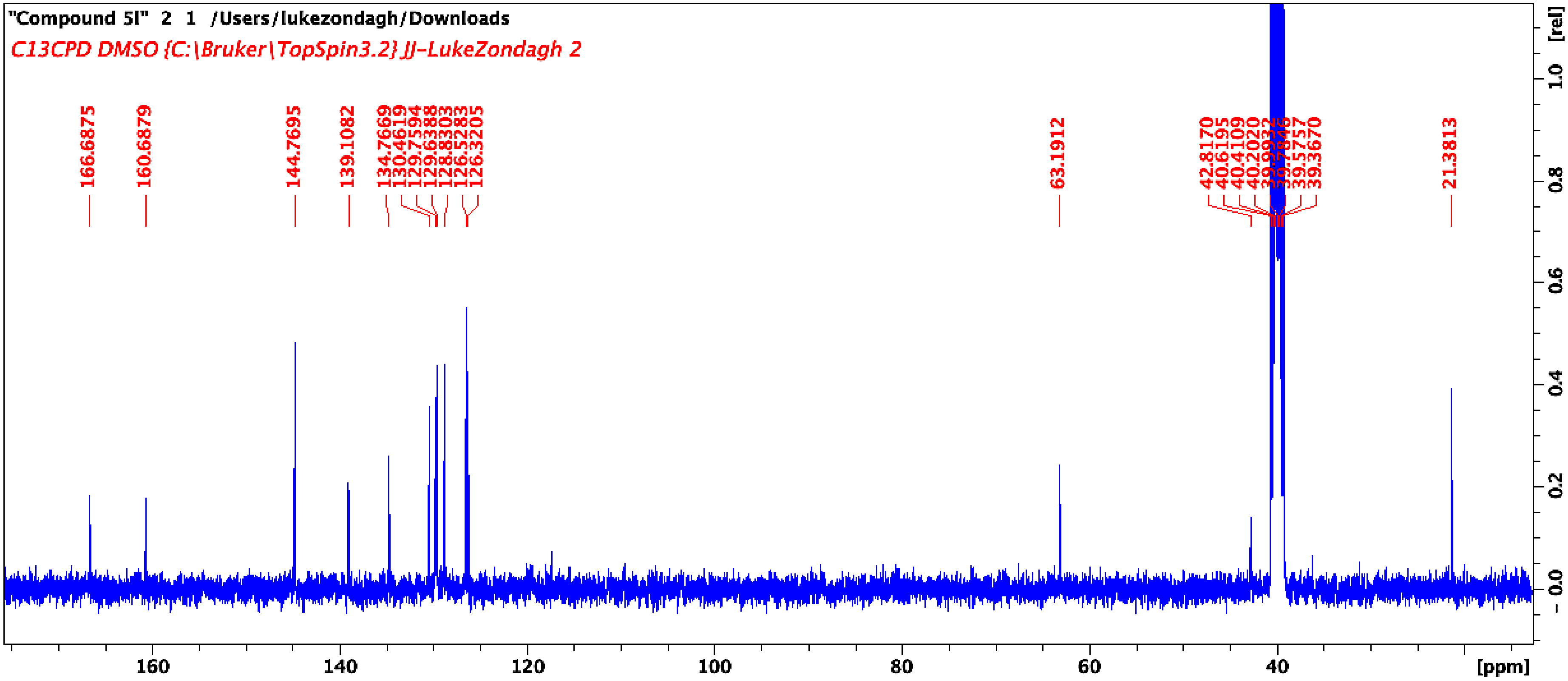

Spectrum 50: HSQC NMR compound 5k

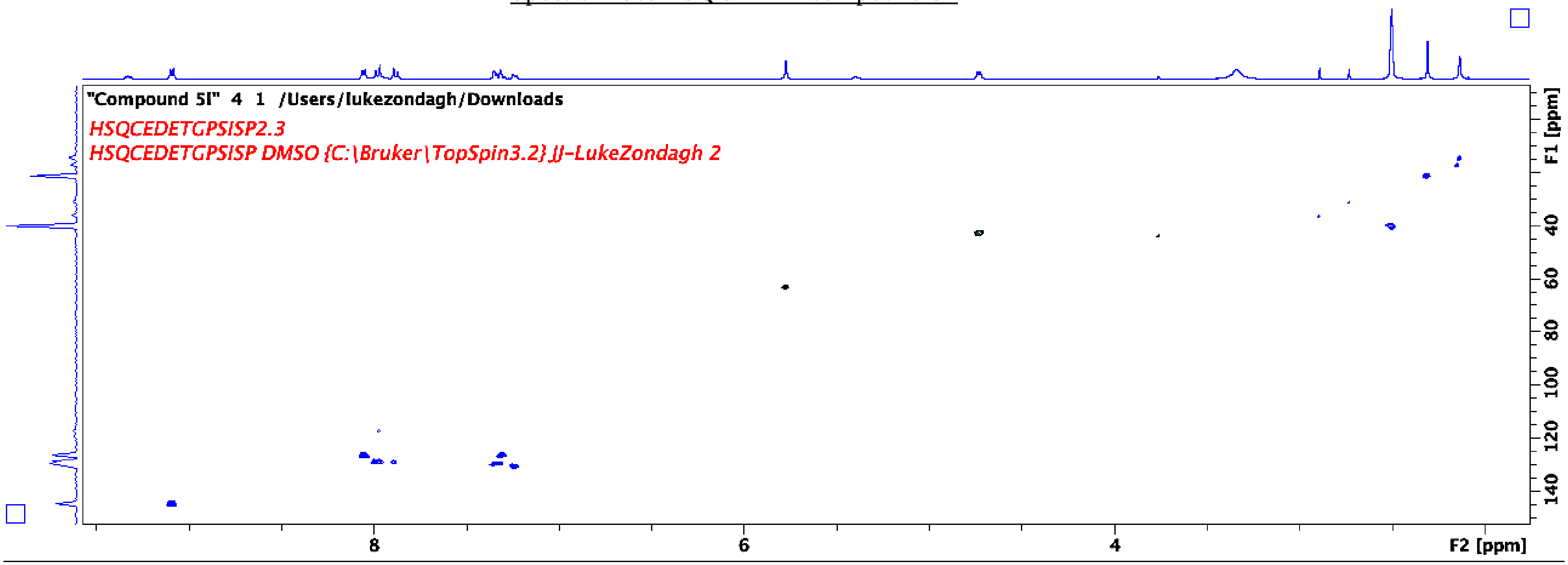

Spectrum 51: IR Compound 5k

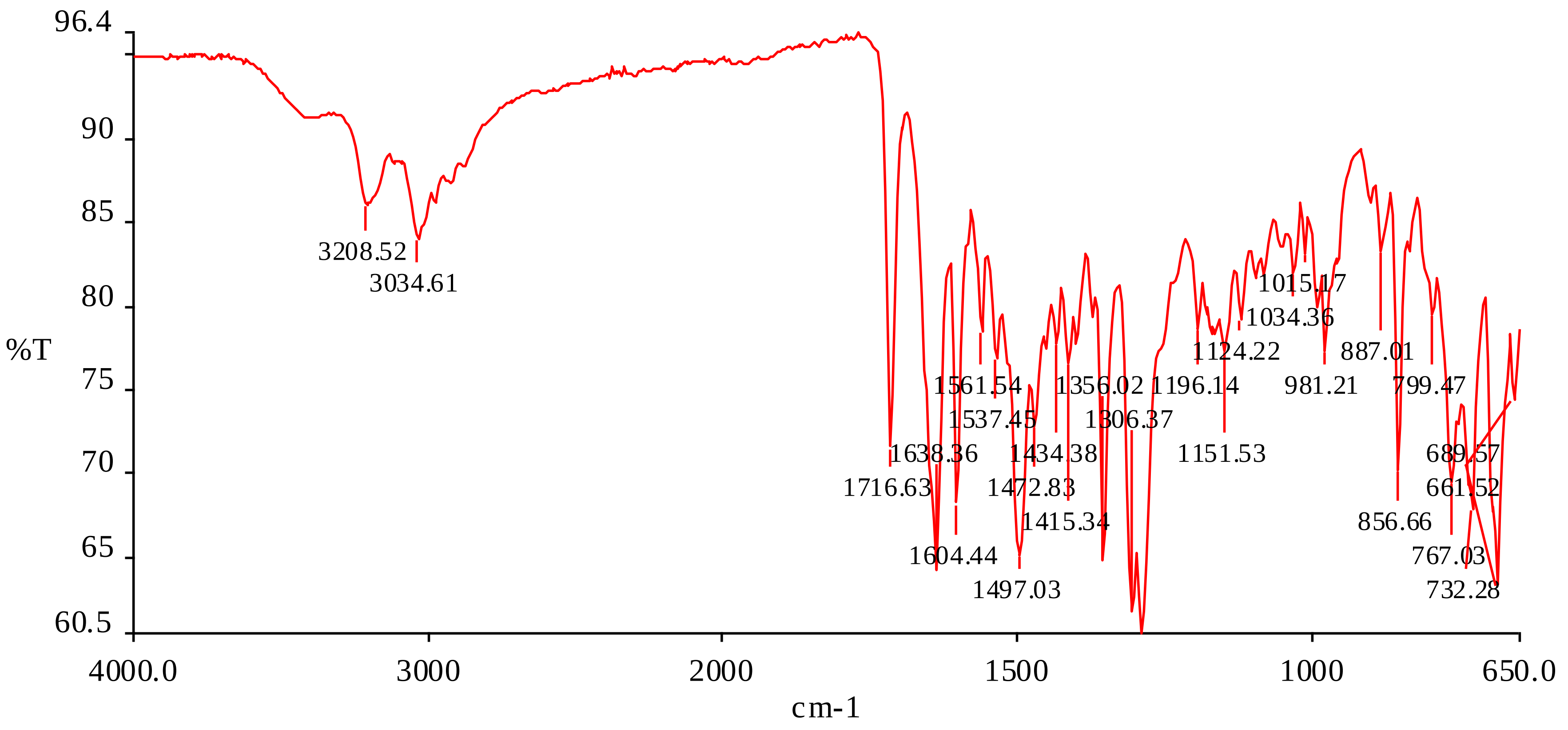

Spectrum 52: MS Compound 5k

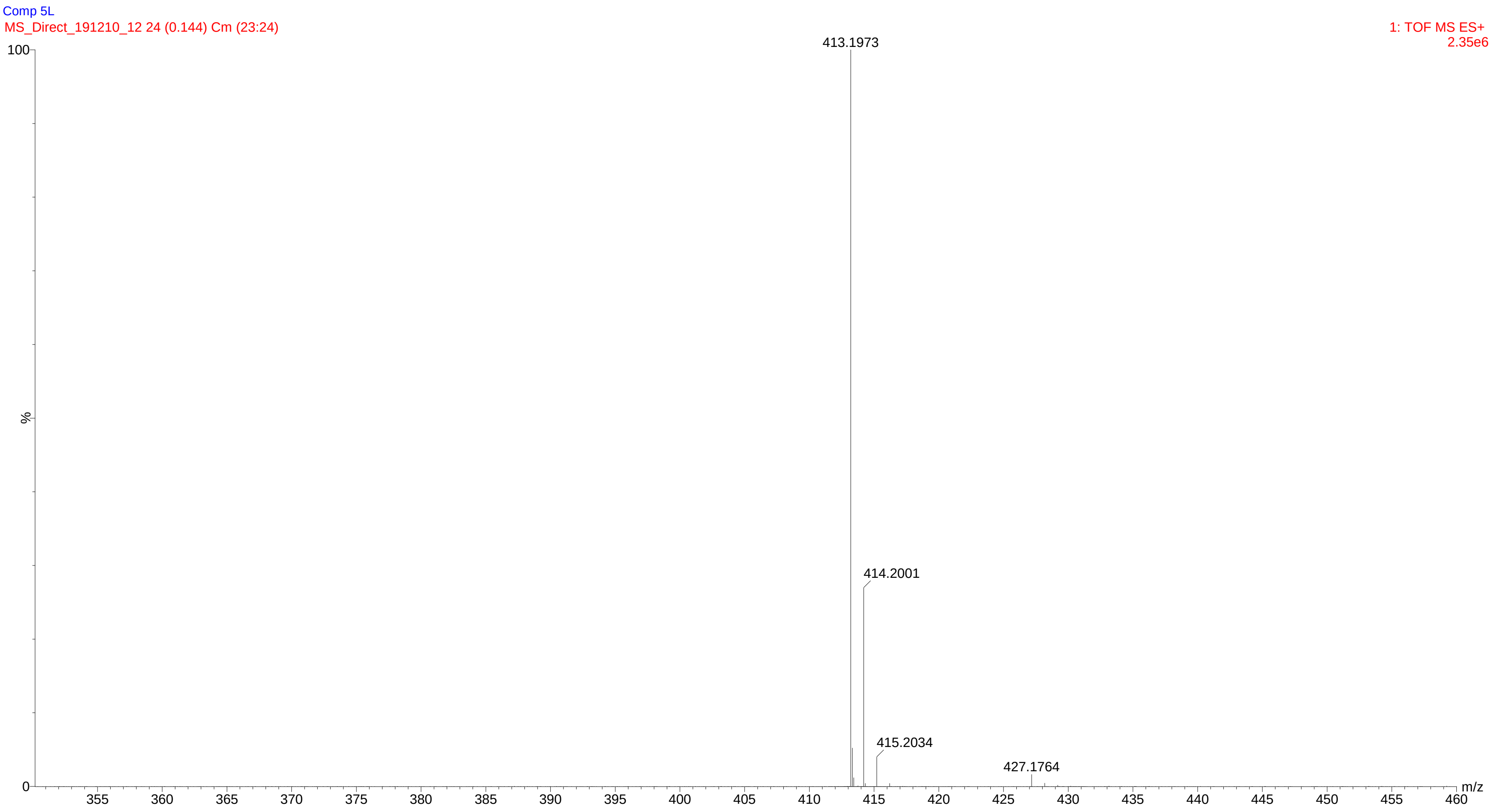

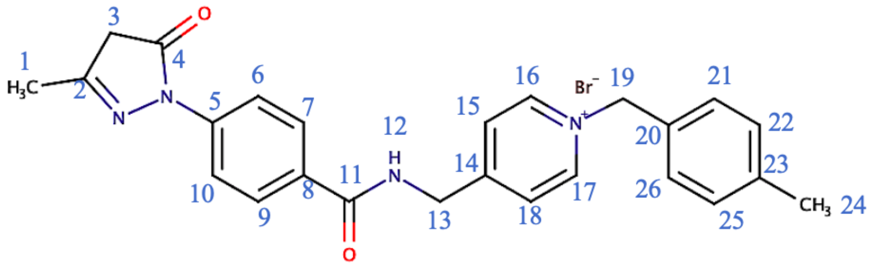

Compound **5l**

Spectrum 53: ^{1}H NMR Compound 5l

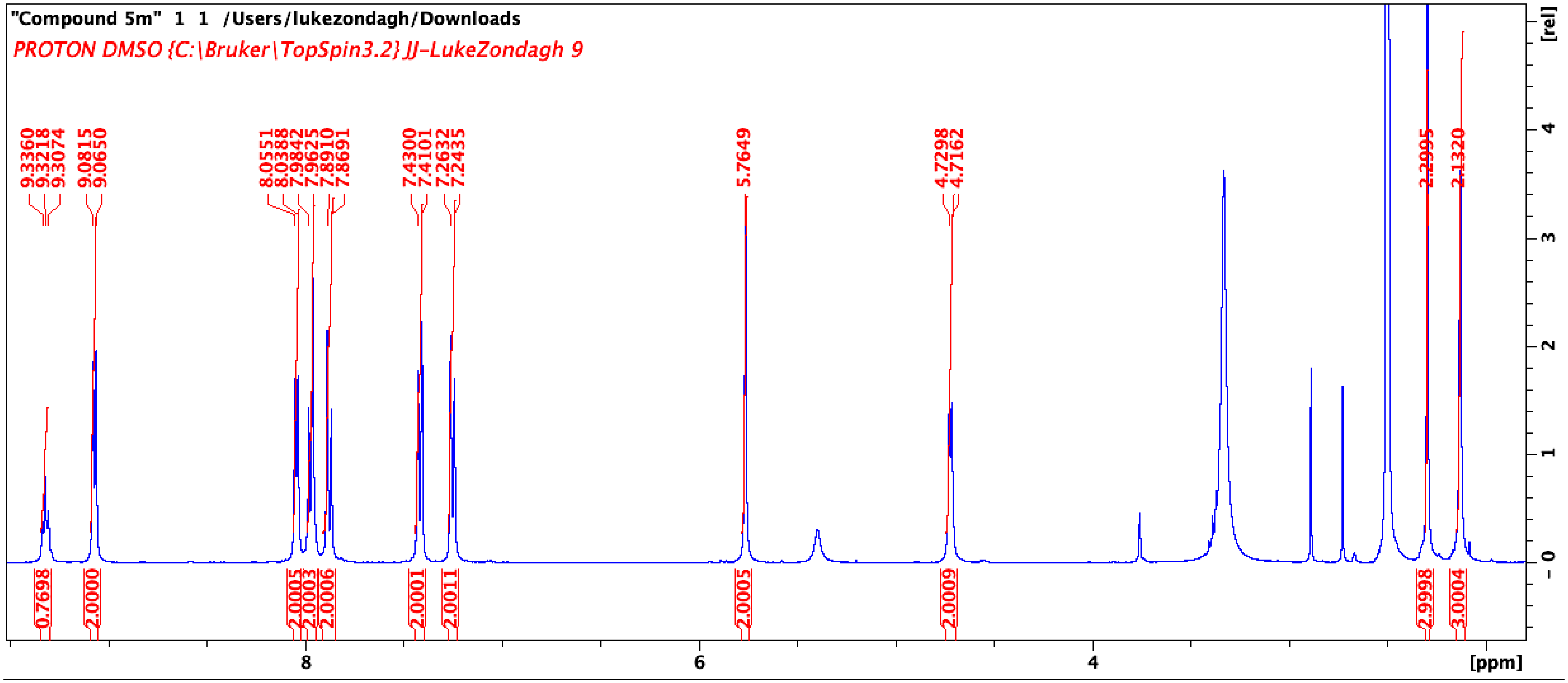

Spectrum 54: ^{13}C NMR Compound 5l

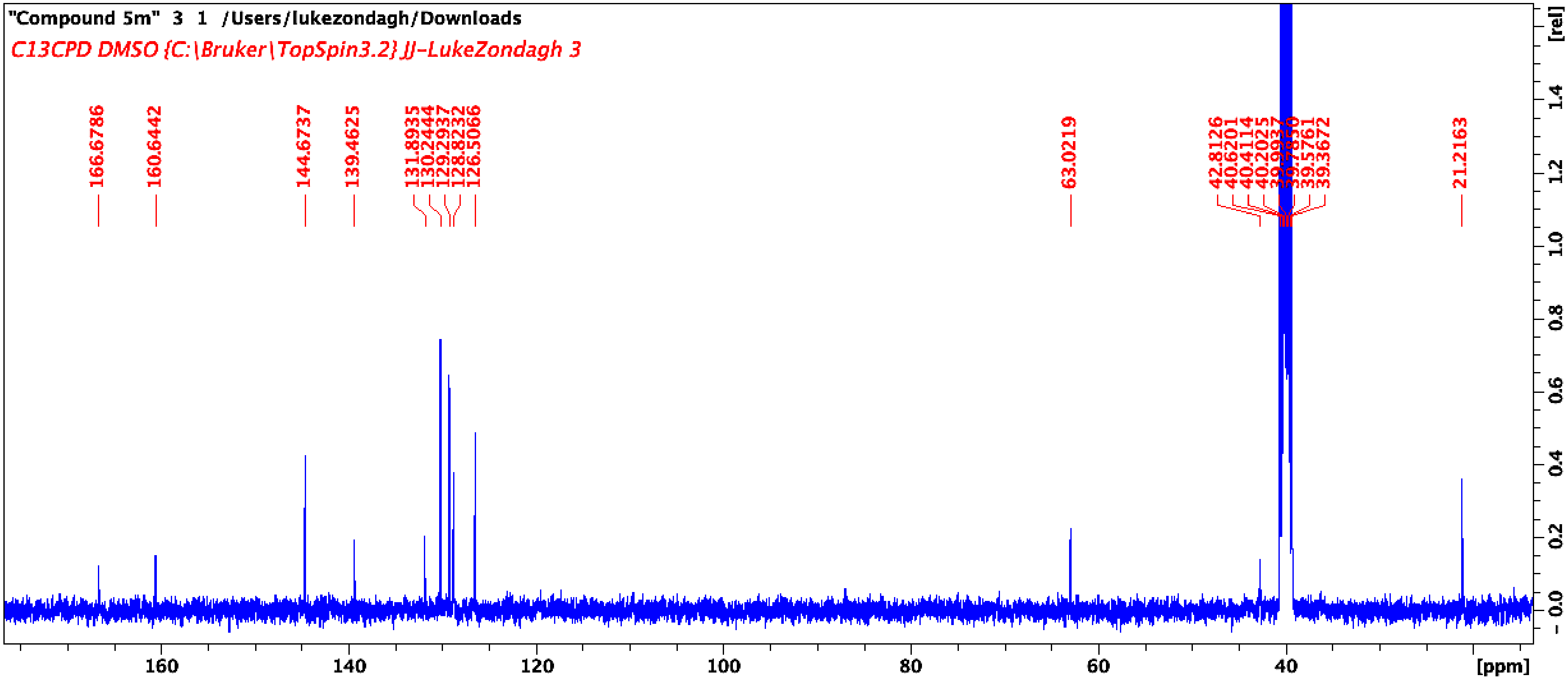

Spectrum 55: HSQC NMR Compound 5l

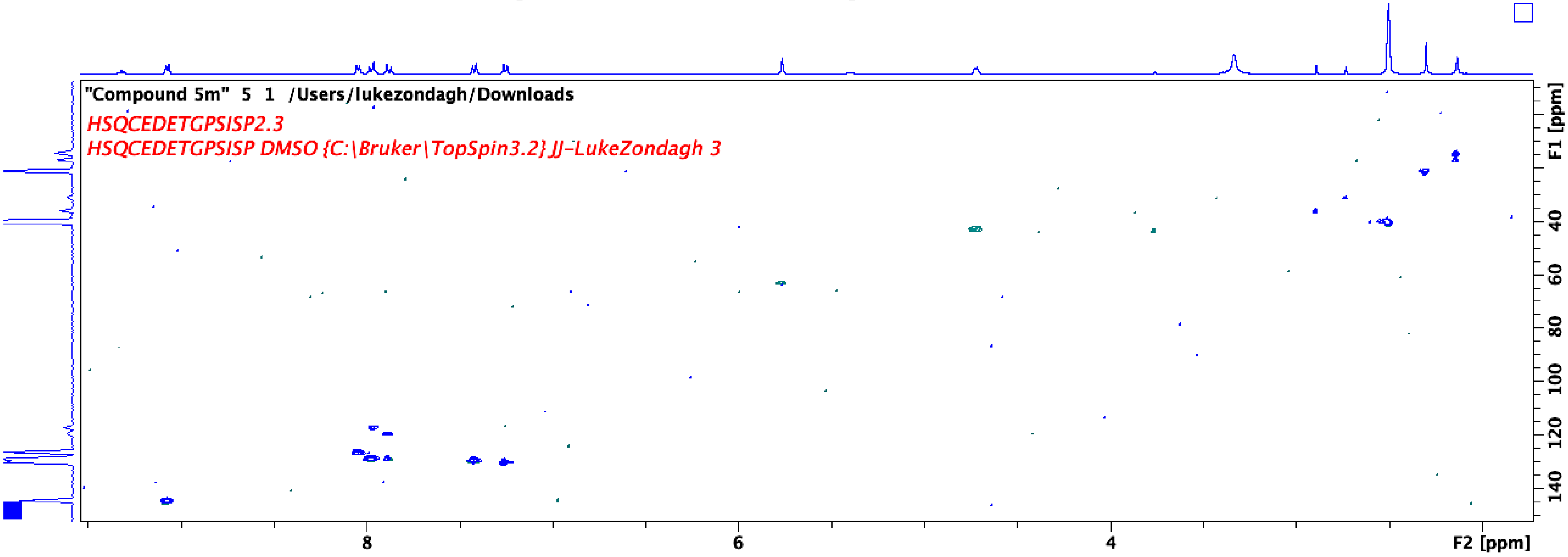

Spectrum 56: IR Compound 5l

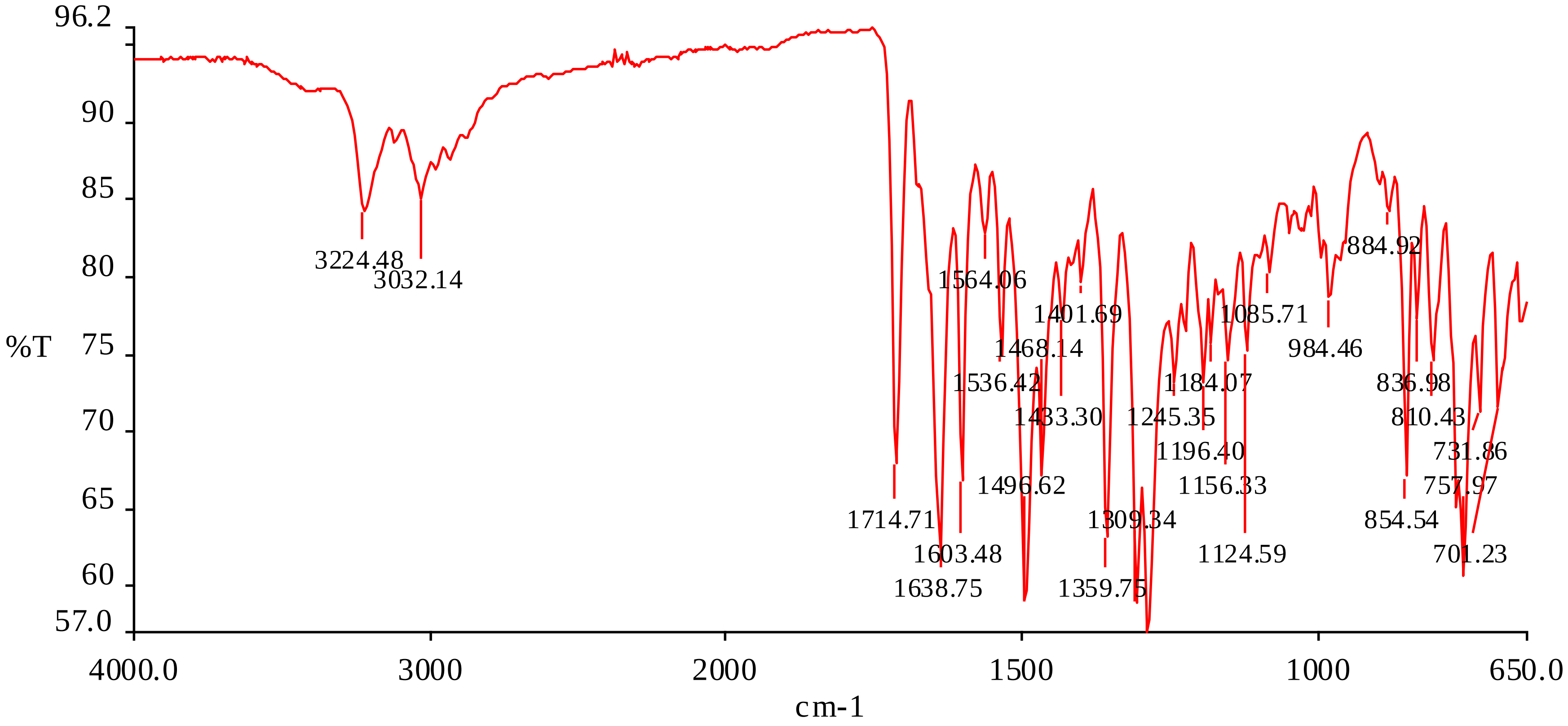

Spectrum 57:MS Compound 5l

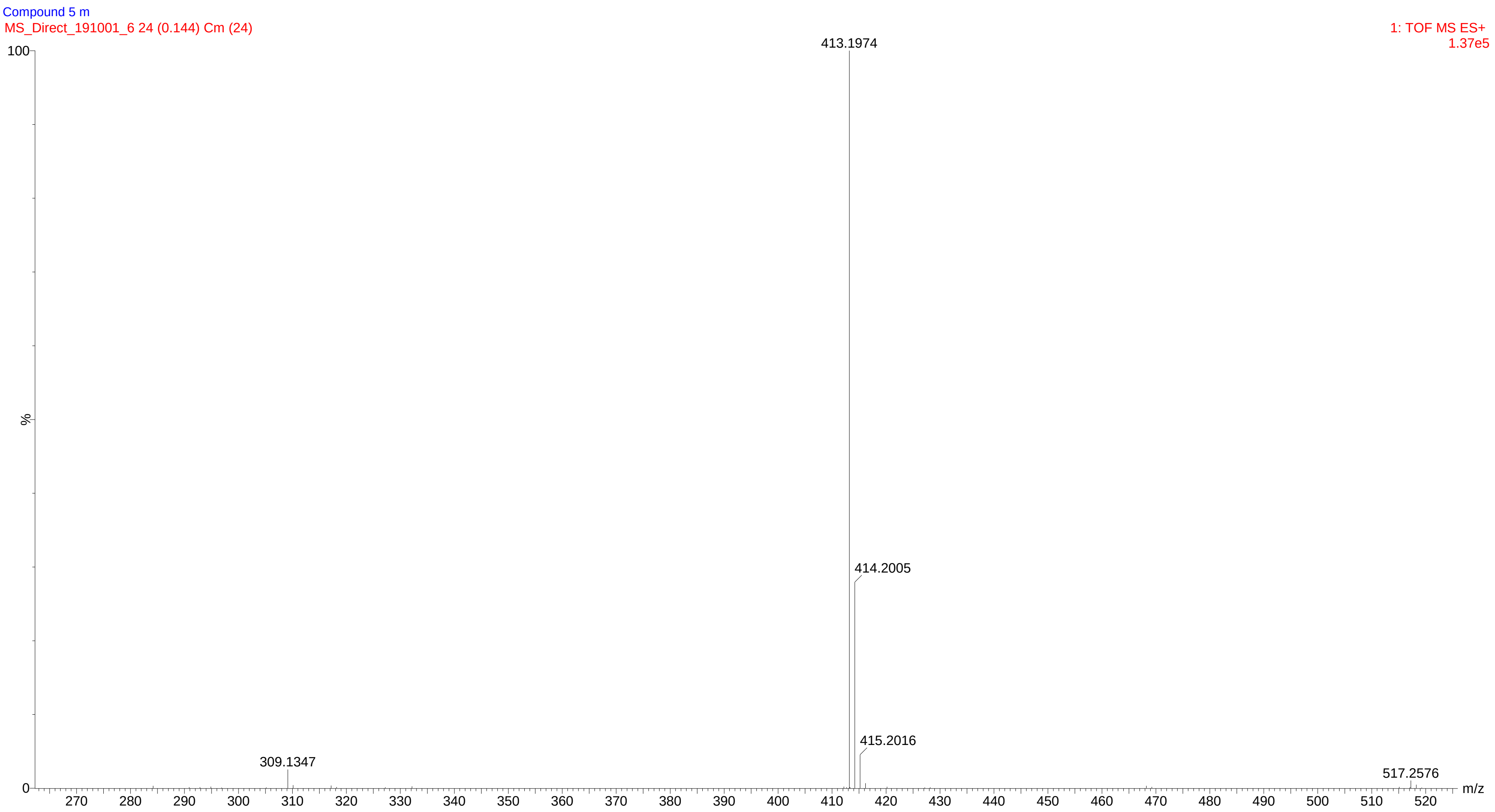

Supplement: Supplemental Material [file IENZ_A_1801673_SM9341.pdf]
